# Bright luminescent lithium and magnesium carbene complexes†‡

**DOI:** 10.1039/d1sc00846c

**Published:** 2021-04-15

**Authors:** Piermaria Pinter, Christoph M. Schüßlbauer, Fabian A. Watt, Nicole Dickmann, Regine Herbst-Irmer, Bernd Morgenstern, Annette Grünwald, Tobias Ullrich, Michael Zimmer, Stephan Hohloch, Dirk M. Guldi, Dominik Munz

**Affiliations:** Department of Chemistry and Pharmacy, Friedrich-Alexander University Erlangen-Nürnberg Egerlandstr. 1-3 D-91058 Erlangen Germany; Interdisciplinary Center for Molecular Materials (ICMM), Friedrich-Alexander University Erlangen-Nürnberg Egerlandstr. 3 D-91058 Erlangen Germany; Department of Chemistry, Inorganic Chemistry, Paderborn University Warburger Straße 100 D-33098 Paderborn Germany; University of Göttingen, Institute of Inorganic Chemistry Tammannstraße 4 D-37077 Göttingen Germany; Inorganic Solid State Chemistry, Saarland University Campus C4.1 D-66123 Saarbrücken Germany; Inorganic Chemistry: Coordination Chemistry, Saarland University Campus C4.1 D-66123 Saarbrücken Germany dominik.munz@uni-saarland.de; Inorganic and General Chemistry, Saarland University Campus C4.1 D-66123 Saarbrücken Germany; Institute of General, Inorganic and Theoretical Chemistry, University of Innsbruck Innrain 80-82 A-6020 Innsbruck Austria

## Abstract

We report on the convenient synthesis of a CNC pincer ligand composed of carbazole and two mesoionic carbenes, as well as the corresponding lithium- and magnesium complexes. Mono-deprotonation affords a rare “naked” amide anion. In contrast to the proligand and its mono-deprotonated form, tri-deprotonated *s*-block complexes show bright luminescence, and their photophysical properties were therefore investigated by absorption- and luminescence spectroscopy. They reveal a quantum yield of 16% in solution at ambient temperature. Detailed quantum-chemical calculations assist in rationalizing the emissive properties based on an Intra-Ligand-Charge-Transfer (ILCT) between the carbazolido- and mesoionic carbene ligands. (Earth-)alkali metals prevent the distortion of the ligand following excitation and, thus, by avoiding non-radiative deactivation support bright luminescence.

## Abstract 2

Lithium- and magnesium complexes of a CNC pincer ligand composed of carbazole and two mesoionic carbenes are bright luminescent, because the metals prevent molecular distortions upon excitation.

## Graphical abstract

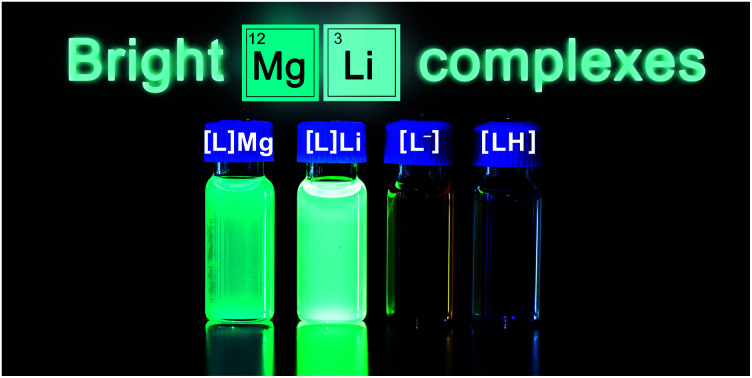

## Introduction

**Fig. 1:**
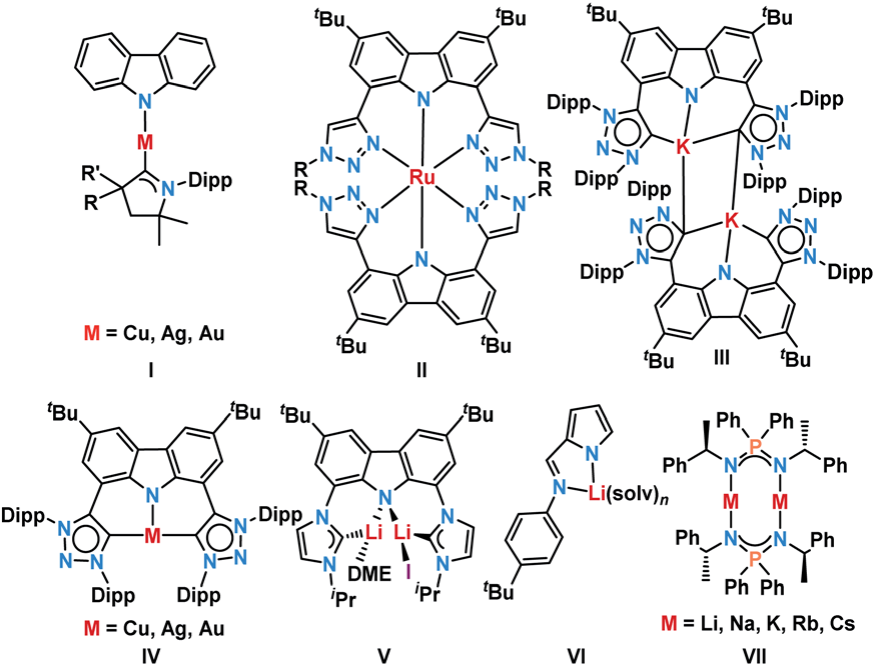
Previously studied related complexes.

Carbazole-based dyes have a rich history as photo-sensitizers,^1–3^ photo-initiators and -catalysts,^4–7^ host materials for Organic Light-Emitting Diodes (OLEDs),^8,9^ triplet emitters,^10,11^ and Thermally Activated Delayed Fluorescence (TADF)^12–18^ materials. Recent remarkable achievements comprise the emissive properties of two-coordinate coinage metal complexes.^19–32^ There, embedding copper(i) in a push–pull electronic environment provided by a π-acidic carbene^33^ and a π-donating carbazolido ligand (Fig. 1, **I**), resulted in a 100% quantum yield of the 474 nm luminescence and an excited state lifetime of 2.8 μs.^22^ The surprising efficiency of these “carbene-metal-amido” complexes relies on a prompt Reverse Inter System Crossing (RISC) mechanism.^34–36^ Furthermore, the donor–bridge–acceptor substitution pattern, as it is known from organic dyes, enhances the transition probability, and thus favors fluorescence over non-radiative decay channels.^37^ We became interested in carbazolyl bridged pincer-type NHC ligands^38–41^ as promising candidates to stabilize multiple bonded late transition metal complexes.^42–44^ Similar ligands, which had been studied mainly as NNN pincers (Fig. 1, **II**),^45–57^ have been pioneered by Bezuidenhout^58–62^ (CNC pincer, C: mesoionic carbene MIC; **III** and **IV**) and Kunz^63–68^ (CNC pincer, C: *N*-heterocyclic carbene NHC; **V**). Mesoionic carbenes,^69–75^ in general, and 1,2,3-triazolinylidenes, in particular, excel through their donating properties^76,77^ and, as such, are expected to stabilize high-valent transition metals.

Despite the fact that they are much less popular than conventional NHCs^42^ and less explored in photochemistry, they increasingly attract attention.^52,78–89^ During our initial coordination experiments with the 1,2,3-triazolinylidene decorated carbazolide obtained by deprotonation of **3**, we noticed strong luminescence upon deprotonation (*vide infra*). Intriguingly, Kunz had already noticed luminescence for lithium carbazolyl bridged dicarbenes, however, these systems have not been studied spectroscopically yet (Fig. 1, **III** and **V**).^59,63,64,90^ Bezuidenhout and co-workers reported the photochemical properties of T-shaped and linear coinage metal complexes (Fig. 1, **IV**).^62^ They found that the luminescence wavelength is tunable by the judicious choice of the metal. Thereby, the copper(i) complex emitted in the blue- (quantum yield *Φ*^em^ = 0.8%), the silver(i) in the orange- (*Φ*^em^ = 2.4%), and the gold(i) complex in the green- (*Φ*^em^ = 0.6%) regions of the spectrum. The protonated proligand showed emissive properties as well (green, *Φ*^em^ = 2.0%). Heinze *et al.*^91^ described, for example, “alkali-blue” emissive pyrrolates (Fig. 1, **VI**). A quantum yield of 1% was achieved through an efficient ILCT (Intra-Ligand-Charge-Transfer) thanks to the templating effect of the alkali metals.^92^ Blue luminescence was also observed by Roesky *et al.* in the case of iminophosphonamide alkali metal complexes (Fig. 1, **VII**) with a solid-state quantum yield of up to 36%.^93^ Agapie and co-workers introduced lithium bridged dipyridyl dipentacene pyrrolates as efficient singlet fission molecules.^94^

Inspired by these reports and the surge of interest in photochemistry with complexes of earth-abundant metals,^95^ we report herein a carbazolide bridged mesoionic biscarbene pincer ligand and a detailed investigation on the luminescent properties of its (earth-)alkali complexes. These complexes show excellent quantum yields of up to 16% at ambient temperatures in solution. Using quantum chemical calculations, we elucidate the effects of rigidity and planarity on the luminescence quantum yield and the excitation/luminescence wavelengths.

## Results and discussion

### Proligand synthesis

Searching for an alternate route to design tridentate carbazole-triazolylidene ligands, avoiding the use of potentially hazardous and explosive *tert*-butylhypochloride as suggested by Bezuidenhout and co-workers,^59^ we initially examined the alkylation of classical triazoles, as has been reported by Limberg, Hecht and Brooker.^45,51^ This strategy proved also successful in case of carbenaporphyrins.^90^ In the present case, neither the use of methyl iodide nor Meerwein's salt (triethyloxonium tetrafluoroborate) or methyl triflate yielded the desired carbazole-bistriazolium salts in reasonable yields. Instead, mixtures with predominantly *N*-carbazole-methylation were observed.

**Scheme 1:**
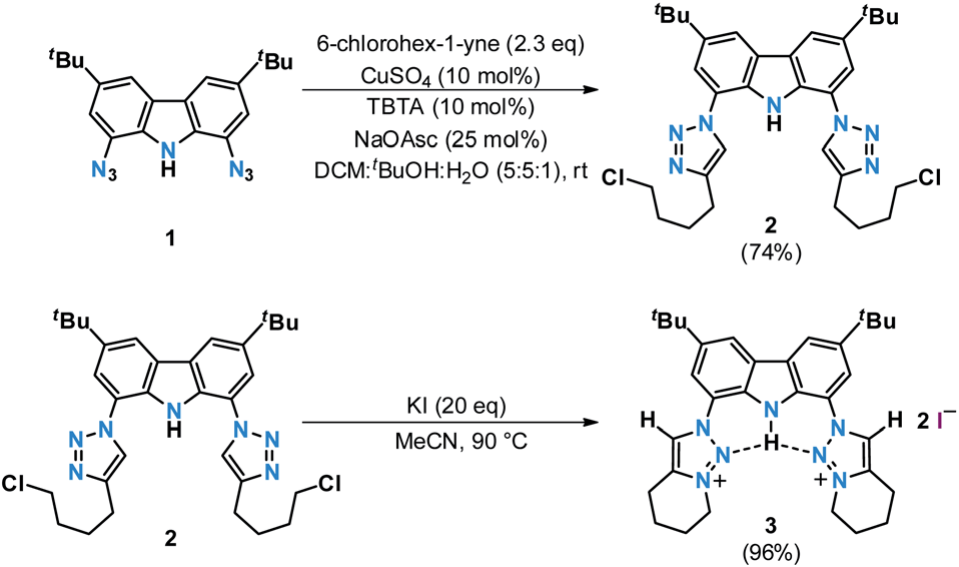
Proligand synthesis *via* intramolecular cyclization. TBTA = tris[(1-benzyl-1*H*-1,2,3-triazol-4-yl)methyl]amine, NaOAsc = sodium ascorbate.

**Fig. 2:**
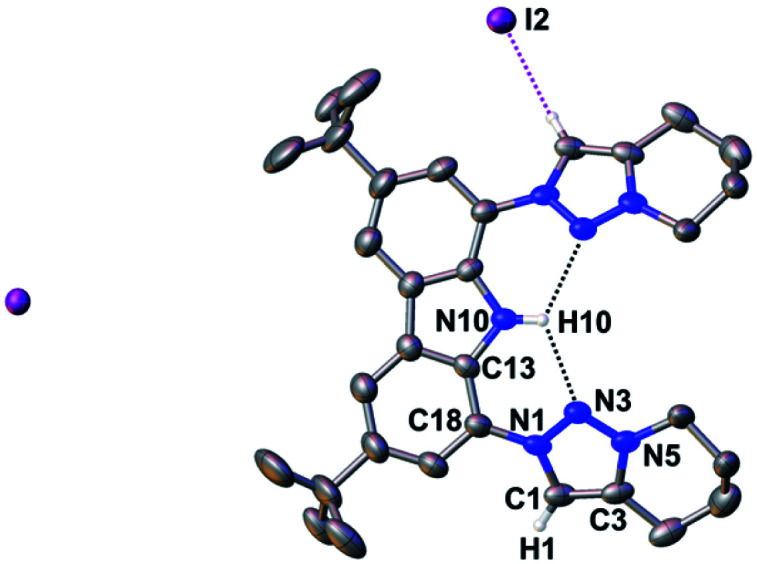
Compound **3** forms intramolecular hydrogen bonds in the solid-state. Thermal ellipsoids are shown at the 50% probability level; hydrogen atoms (except the ones bonded to the carbazole's nitrogen atom and the triazolium heterocycles) are omitted for clarity. Selected bond lengths [Å], angles [°], and dihedral angle [°]: N10–H10 0.88(2), N10–C13 1.391(6), C18–N1 1.433(7), N1–N3 1.326(6), N3–N5 1.311(6), C1–C3 1.353(8), N3–H10 2.351(19), C13–N10–C13 107.0(6), N1–C1–C3 105.8(5), C13–C18–N1–N3 29.2(7).

To avoid the undesired *N*-carbazole alkylation, we consequently adopted an intramolecular alkylation strategy (Scheme 1).^96^ The synthesis of **1** was achieved following nitration, reduction, and azotation of commercially available 3,6-di-*tert*-butylcarbazole.^51^ Using standard CuAAC (Cu-catalyzed Azide–Alkyne Cycloaddition) conditions with 6-chlorohex-1-yne led to clean **2**. The formation of the product was apparent from the disappearance of the characteristic azide stretching resonance at 2099 cm^−1^ (Fig. S1†) and by the characteristic low-field ^1^H NMR resonance at *δ* = 7.98 ppm indicative for a triazole heterocycle (Fig. S2†). Adding excess of potassium iodide and heating the mixture in acetonitrile to reflux for two days gave the *N*-fused triazolium salt **3** in essentially quantitative yields. The cyclization was evident by several features in the NMR spectra, namely (i) the low-field shift of the triazolium-5*H* resonance in the ^1^H NMR spectrum from *δ* = 7.98 ppm (**2**) to *δ* = 8.91 ppm (**3**), (ii) the low-field shift of the methylene protons' resonance of the former –C*H*_2_Cl group from *δ* = 3.64 ppm (**2**) to *δ* = 4.79 ppm (**3**), and (iii) the coupling of this methylene group to one of the triazolin nitrogen atoms according to two-dimensional ^1^H–^15^N HMBC spectra (Fig. S6 and S11†). X-ray quality crystals^97^ of **3** were obtained by slow evaporation of a saturated chloroform solution (Fig. 2). The triazolium salt **3** crystallized with four strongly disordered molecules of chloroform in the lattice in the orthorhombic space group *Pbcm* with half a molecule of **3** in the asymmetric unit.

The structural metrics of the dication in **3** (Table S3†) resemble those of previously reported triazolium salts.^98,99^ In the solid-state structure of **3**, strong hydrogen bonding interactions between the central triazolium nitrogen atoms N3 and the carbazole proton H10 were observed. Due to steric bulk, these interactions are not feasible for the other reported MIC-CNC pincer ligands (Fig. 1**III** and **IV**), but have been observed in a bis(pyrazolyl)carbazole derivative.^100^ Additionally, weak hydrogen bonding between H10–I2 and H1–I2 were observed in the solid-state structure of **3** (Fig. S29†).

### Complex synthesis

**Scheme 2:**
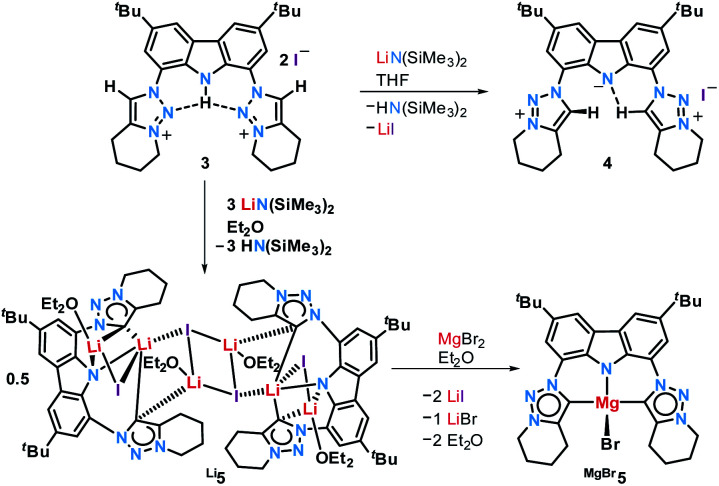
Deprotonation of **3** to **4** and **Li5** and subsequent transmetalation to **MgBr5**.

**Fig. 3:**
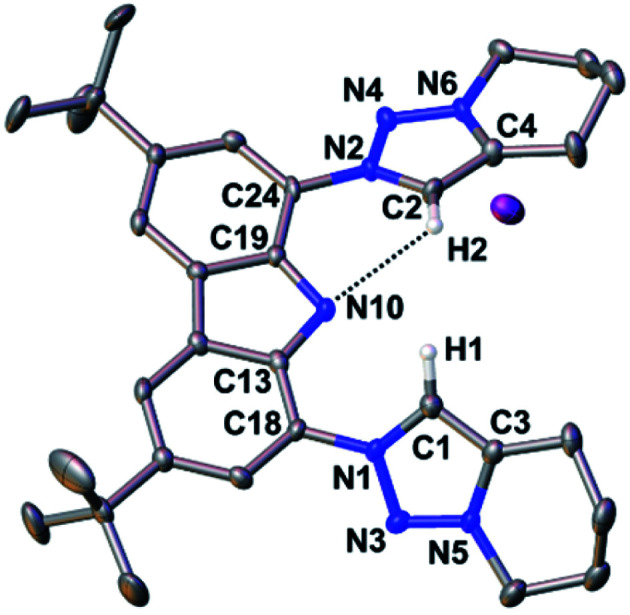
Compound **4** is devoid of N-coordinated lithium in the solid-state. Thermal ellipsoids are shown at the 50% probability level; hydrogen atoms (except the ones bonded to the triazolium heterocycles) are omitted for clarity. Selected bond lengths [Å], angles [°], and dihedral angles [°]: N10–C13 1.373(4), N10–C19 1.368(6), C24–N2 1.434(4), N2–C2 1.351(4), C2–C4 1.361(5), C4–N6 1.354(4), N6–N4 1.322(4), N4–N2 1.335(4), N10–H2 2.397(2), N10–H1 2.909(4), C18–N1 1.437(4), N1–N3 1.324(4), N5–N3 1.324(4), N5–C3 1.351(5), C3–C1 1.361(5), C13–N10–C19 103.1(3), N2–C2–C4 106.0(3), N1–C1–C3 105.7(3), C13–C18–N1–N3 −137.7(4), C19–C24–N2–N4 162.9(4).

**Fig. 4:**
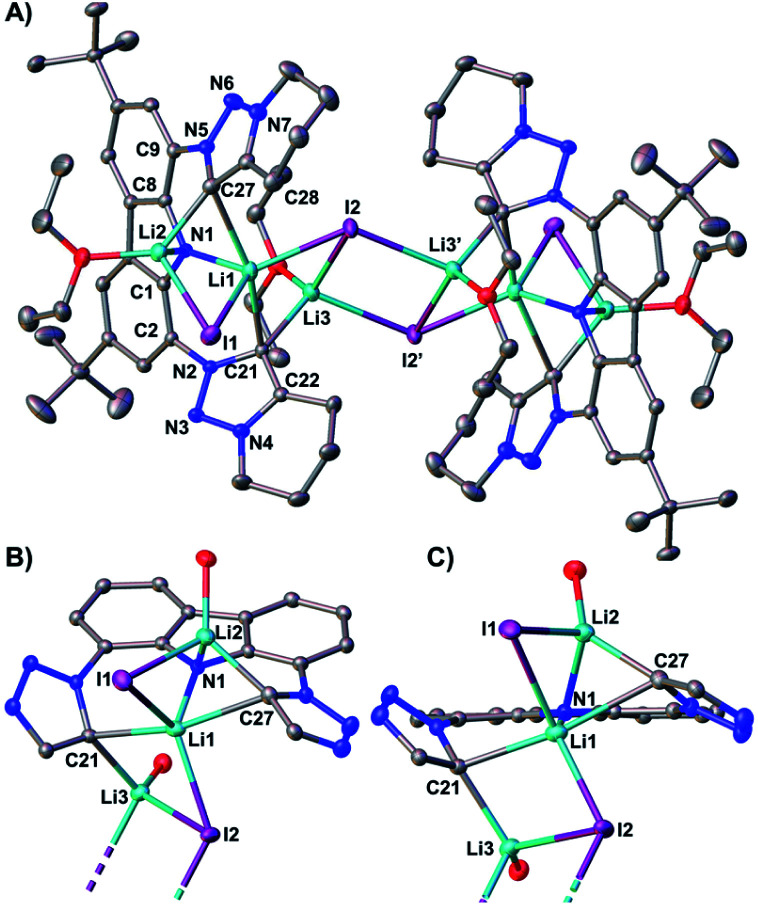
(A) The molecular structure of dimeric **Li5** contains six lithium atoms and four bridging iodo ligands. Thermal ellipsoids are shown at the 50% probability level; hydrogen atoms are omitted for clarity. The monomers are shown from different views in (B) and (C), for which the annulated six-membered rings, ^*t*^Bu groups, ethyl fragments of the diethyl ether and all hydrogen atoms have been omitted for clarity. Selected bond lengths [Å], angles [°], and dihedral angles [°]: N1–C1 1.3837(19), N1–C8 1.3899(19), C9–N5 1.4302(19), N5–N6 1.3396(18), N6–N7 1.3251(19), N7–C28 1.356(2), C28–C27 1.392(2), C27–N5 1.373(2), C27–Li2 2.136(3), Li2–I1 2.776(3), I1–Li1 2.911(3), Li1–I2 2.748(3), Li1–I1 2.911(3), C27–Li1 2.633(3), Li3–I2′ 2.850(3), Li3–I2 2.970(3), C21–Li1 2.391(3), C21–Li3 2.216(3), C21–N2 1.3771(19), N2–N3 1.3414(17), N3–N4 1.3249(18), N4–C22 1.357(2), C22–C21 1.398(2), C2–N2 1.4348(19), N1–Li1 2.052(3), N1–Li2 2.188(3), C1–N1–C8 103.18(12), N2–C21–C22 100.22(12), N5–C27–C28 100.25(13), C1–C2–N2–N3 137.4(1), C8–C9–N5–N6 151.3(1).

The reaction of proligand **3** with one equivalent of lithium hexamethyl disilazide [LiHMDS; LiN(SiMe_3_)_2_] led to deprotonation of the carbazole (**4**, Scheme 2). Evidence for the latter came from the disappearance of the resonance for the carbazole N–*H* group in the ^1^H NMR spectrum (Fig. S12†). X-ray quality crystals of deep-orange and air-stable **4**, which crystallized in the *P*1̄ space group, were obtained by diffusion of hexane into a THF/benzene solution (Fig. 3). Notably, the carbazolide in **4** does not coordinate a lithium cation. Instead, the latter precipitated from the reaction mixture in the form of lithium iodide. Compound **4**, therefore, contains a rare “naked” amide anion, as was also corroborated by calculations (Fig. S31†).^101–103^ The structure in the solid-state reveals a weak hydrogen bond [2.397(2) Å] between N10 and H2, which might be the reason for the surprising stability of **4** towards moisture. Proligand **3** was also deprotonated thrice by three equivalents of LiHMDS, as was confirmed by the ^1^H NMR spectroscopic analysis of **Li5** (Fig. S14†). The ^7^Li NMR of **Li5** showed a signal at *δ* = −1.43 ppm, which corroborates the presence of lithium cations (Fig. S16†). Immediately upon deprotonation of **3**, strong blue luminescence was observed (*vide infra*) even in dim light. X-ray quality crystals of **Li5** could be obtained by slow diffusion of pentane into a diethyl ether solution of the complex (Fig. 4A). **Li5** crystallized in the monoclinic space group *P*2_1_/*n*. It has a dimeric structure in the solid-state with bridging *μ*-iodo ligands and includes lithium iodide adducts. Thereby, the Li2–I1 and Li3–I2 units could be understood as lithium iodide molecules.

The Li2–I1 bond length [2.776(3) Å] is comparable with that observed in lithium iodide adducts (2.70–2.80 Å). Also the larger Li3–I2 distance [2.970(3) Å] is in the range of previously reported lithium iodide clusters (2.98 Å).^104–108^ The N1 nitrogen atom of the carbazolido- (Fig. 4B and C), as well as the MIC-ligands, thus coordinate one lithium atom (Li1) and formally another molecule of lithium iodide (Li2–I1 and Li3–I2, respectively). Overall, this arrangement locks the central Li1 atom in place. Eventually, the lability of the lithium iodide was corroborated by elemental analysis (ESI†). Repeated re-crystallization lowered the equivalents of lithium iodide from two, as present in the solid-state structure shown in Fig. 4, to 0.3 equivalents.

Complex **MgBr5** was prepared by transmetalation of **Li5** with MgBr_2_, but may be as well prepared directly from **3** and the Grignard reagent MeMgBr.^109^ We were not able to obtain single crystals of sufficient quality for elucidation of the structure in the solid-state.^110^ However, a diffusion NMR experiment (DOSY) revealed that solutions of **4** and **MgBr5** are mononuclear in deuterated benzene, whereas **Li5** remains a dimer (page S17). Like blue-green luminescent **Li5** (and in contrast to non-emissive **3** and weakly emissive **4**) complex **MgBr5** showed intense, lime-green luminescence in solution, whereas all investigated compounds were essentially non-luminescent in the solid state.

### Luminescent properties

**Fig. 5:**
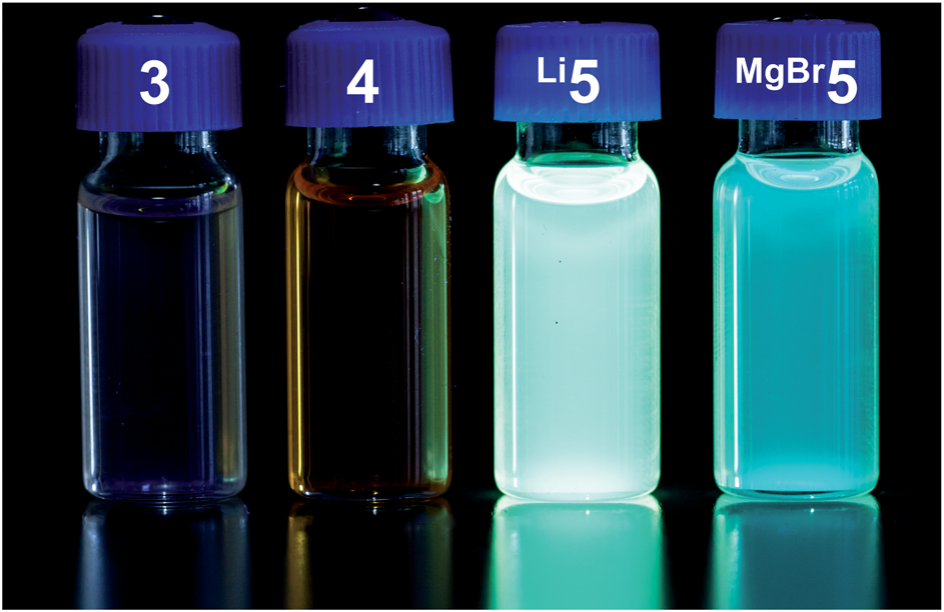
**Li5** and **MgBr5** are luminescent, whereas **3** and **4** are undetectable to low luminescent. The picture was obtained upon irradiation of 1 mM benzene solutions with a common laboratory UV lamp (360 nm).

**Fig. 6:**
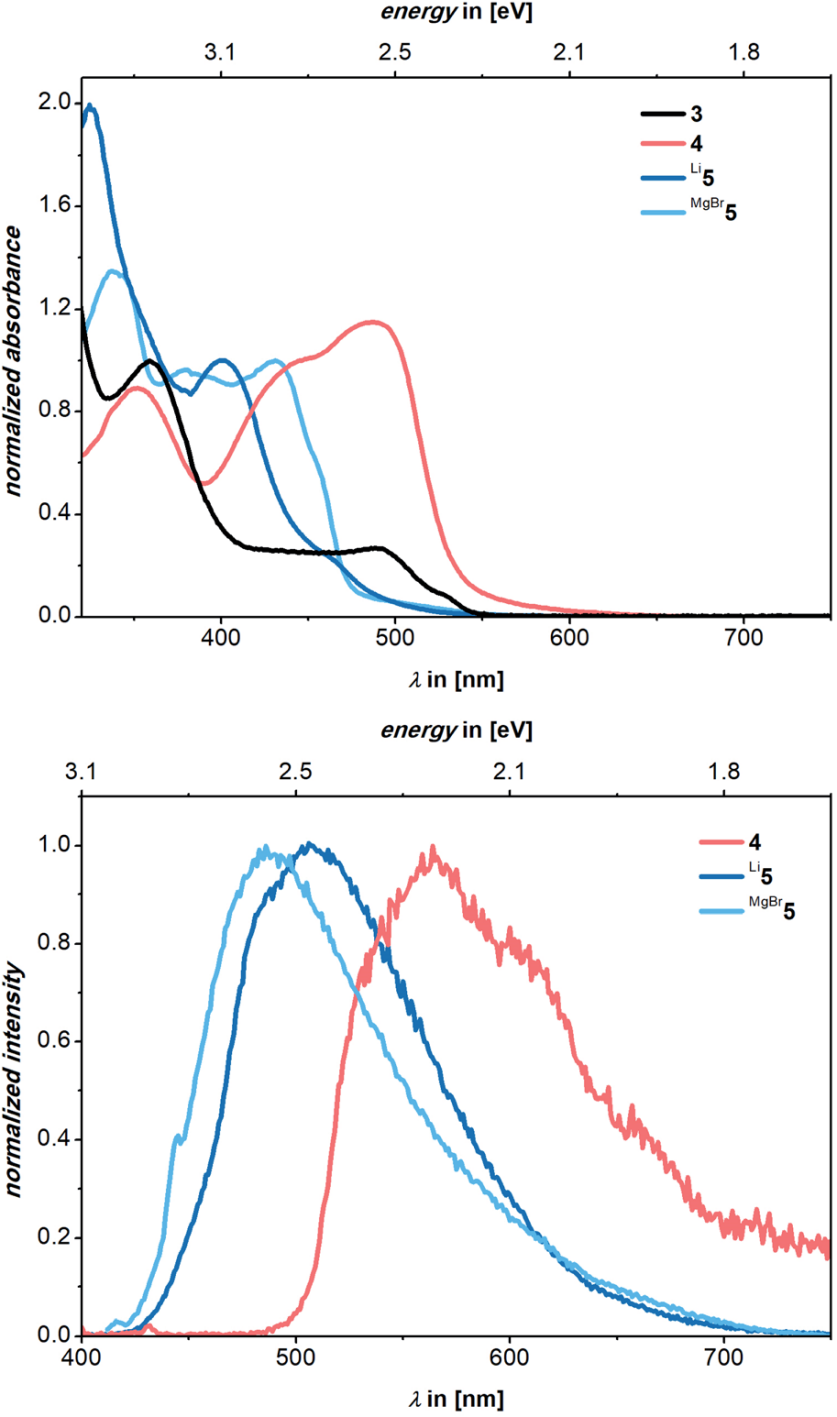
Normalized absorption (top) and luminescence (bottom) spectra of **3** (black), **4** (red), **Li5** (dark blue), and **MgBr5** (light blue) in benzene. The luminescence was recorded upon photoexcitation at 390 nm. Absorbance spectra are normalized at their 360, 445, 402, and 431 nm maxima, respectively, and luminescence spectra to the highest feature.

The bright luminescence motivated more detailed photophysical studies of all compounds. In benzene solutions, very strong luminescence is seen for **Li5**, strong luminescence for **MgBr5**, and undetectable to low luminescence for **3** and **4**Fig. 5. Pertinent spectroscopic features are summarized in Table 1, normalized steady-state absorption and luminescence spectra are shown in Fig. 6.

**Table 1:** Key spectroscopic data of **3**, **4**, **Li5**, and **MgBr5**^a^

Compound	*λ* ^abs,max^/nm	*λ* ^em^/nm	*Φ* ^em^/%	*E* ^Stokes^/eV (cm^−1^)
**3**	360, (492)^b^	—	—	—
**4**	352, 445, 487	565	2	0.35 (2835)
**Li5**	325, 402, 465	506	16	0.23 (2016)
**MgBr5**	339, 380, 431	482	14	0.30 (2455)

a Spectroscopic data were obtained at room-temperature for 1 × 10^−5^ M benzene solutions of **3**, **4**, **Li5**, and **MgBr5**. The emission wavelengths and quantum yields were obtained after excitation at 390 nm.

b The plateau is assigned to vibronic transitions as discussed below.

Although the biscationic proligand **3** is poorly soluble in organic solvents, maxima evolved at 297 and 360 nm. It features a plateau-like shoulder peaking at 492 nm and reaching up to approximately 570 nm. Upon photoexcitation in benzene, **3** is found to be non-luminescent. In contrast, in the absorption spectra of **4**, we find maxima at 352, 445, and 487 nm. Photoexcitation of **4** at, for example, 390 nm leads to a broad and undefined luminescence with a maximum at 565 nm and a 2% *Φ*^em^ (Fig. 6). Turning to the absorption spectrum of **Li5**, two maxima are discernable at 325 and 402 nm. A tail is superimposed onto the latter all the way to 600 nm including a minor shoulder centered at around 465 nm. Excitation spectra for **Li5** and **MgBr5** reveal that the lowest absorption wavelength which leads to luminescence is 465 nm for **Li5** and 434 nm for **MgBr5**. In both cases, this is in line with their absorption maxima (Fig. S21†). We determined Stokes shifts of 0.35, 0.30, and 0.23 eV for **4**, **MgBr5** and **Li5**, respectively.

Excitation of **Li5** at 390 nm gives a luminescence maximum at 506 nm and a luminescence quantum yield of 16%. The luminescence spectrum of **Li5** also shows a tail up to 725 nm. Turning finally to **MgBr5**, we note absorption maxima at 339, 380, and 431 nm. Once again, the latter features a tail up to approximately 560 nm.

Photoexcitation of **MgBr5** at 390 nm leads to a luminescence that reaches a maximum at 487 nm with a quantum yield of 14%. Again, a tail up to 730 nm is noted. Upon exposure of solutions of **Li5** and **MgBr5** to air, the spectroscopic signatures of mono-deprotonated **4** were regenerated (Fig. S21 and S22†). Time-Correlated Single-Photon Counting (TCSPC) experiments on **Li5** and **MgBr5** revealed a luminescence decay with lifetimes of (11.8 ± 1.6 × 10^−2^) and (10.7 ± 4.8 × 10^−2^) ns, respectively (Fig. S25 and S26†). By virtue of lifetime components which are in the typical range for singlet excited states, we rule out the involvement of triplet excited state species as is the case for TADF or phosphorescence. In other words, the luminescent deactivation is fluorescence.

### Computational analysis

**Fig. 7:**
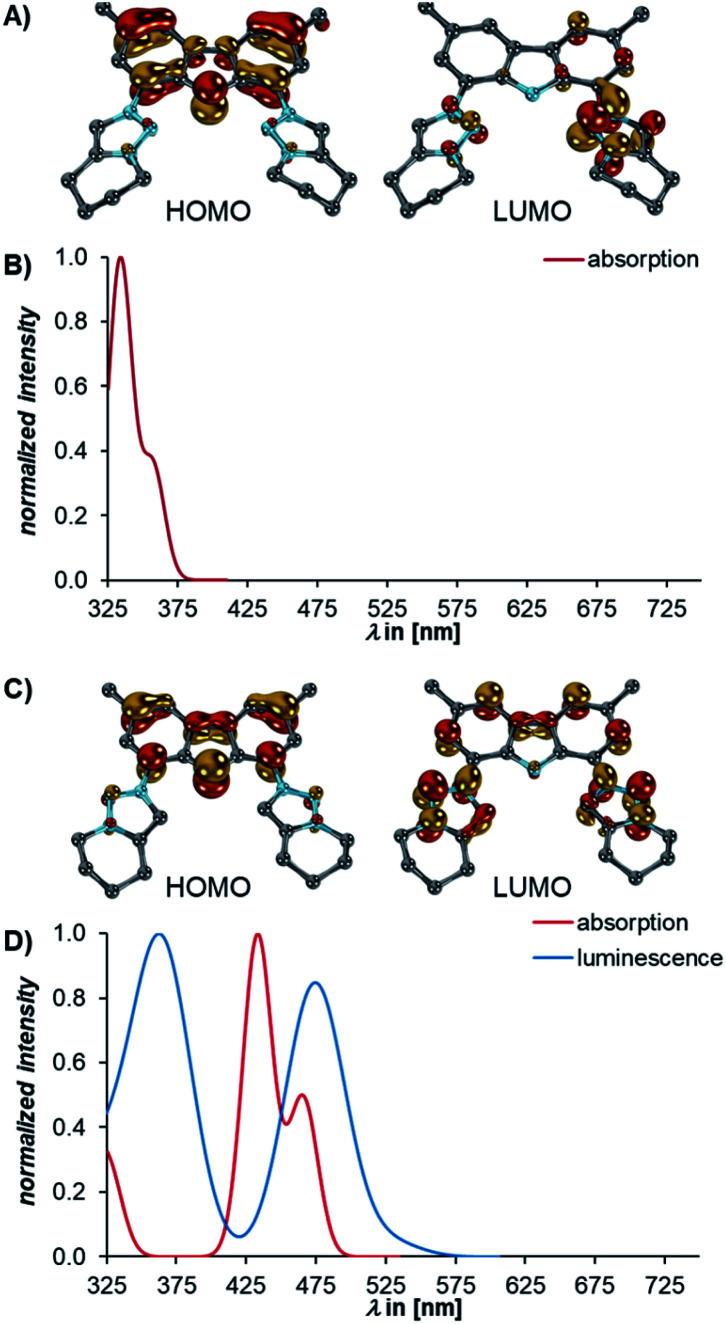
The dominant absorptions and concomitant excitations to the S_1_ states of **3** and **4** belong to the HOMO → LUMO transitions (B and D, red). For both **3** and **4**, the HOMOs (left) are located at the carbazole framework, whereas the LUMOs (right) are mainly localized on one of the triazolium groups (**3**, A) or both MIC units (**4**, C). While **3** is non-luminescent, **4** shows a weak luminescence from the S_1_ state (D, blue). Orbitals were obtained at the TD-DFT(SMD=C_6_H_6_)/def2-TZVPP//B3LYP-D3(BJ)/def2-SVP level of theory, whereas the luminescence spectra were obtained at the STEOM-CCSD(SMD=C_6_H_6_)/def2-TZVPP//B3LYP-D3(BJ)/def2-SVP level of theory. Hydrogen atoms are omitted for clarity.

Puzzled by the optical properties, we investigated the electronic and structural properties of all compounds in their excited states. Exploratory TD-DFT calculations revealed that the transitions to the S_1_ state of **3** and **4** are ILCT (approximated HOMO→LUMO, Fig. 7A and C) processes.

The HOMO is located at the carbazolido ligand, whereas the LUMO is associated with the MICs (*cf.* Fig. S32†). For an accurate description of the charge-transfer states, which is challenging for TD-DFT^111–113^ (Fig. S33–S35†), absorption spectra were computed with the more suitable *ab initio* method “Similarly Transformed Equation of Motion Coupled Cluster Singles and Doubles” (STEOM-CCSD)^114–116^ using the “Domain-based Local Pair Natural Orbital (DLPNO)” approximation.^117,118^ Indeed, this method reproduced the absorption spectra including the transitions to the S_1_ states best (**3**, *f*^osc^ = 0.13, ^calcd^*λ*^abs^ = 357 nm, ^exp^*λ*^abs^ = 360 nm, Fig. 7B; **4**, *f*^osc^ = 0.19, ^calcd^*λ*^abs^ = 466 nm, ^exp^*λ*^abs^ = 487 nm, Fig. 7D).

The luminescence from the S_1_ state of **4** is predicted to be weak (*f*^osc^ = 0.01, ^calcd^*λ*^em^ = 528 nm, ^exp^*λ*^em^ = 565 nm, Fig. 7D) which is in agreement with the observation that **4** is weakly luminescent with a 2% quantum yield. For **3**, neither TD-DFT nor STEOM-CCSD reproduced the plateau between 425 and 525 nm. However, the calculation of the vibronically resolved absorption spectrum using excited state dynamics (Fig. S36†) shows that this shoulder arises from molecular vibrations.

Subsequently, we investigated the electronic structures of the complexes **5**. The calculations revealed that the dimeric structure of **Li5** could be well modelled by calculating the electronic structure of the truncated monomer, thereby omitting the diethyl ether molecules as well (Fig. S32†).

**Fig. 8:**
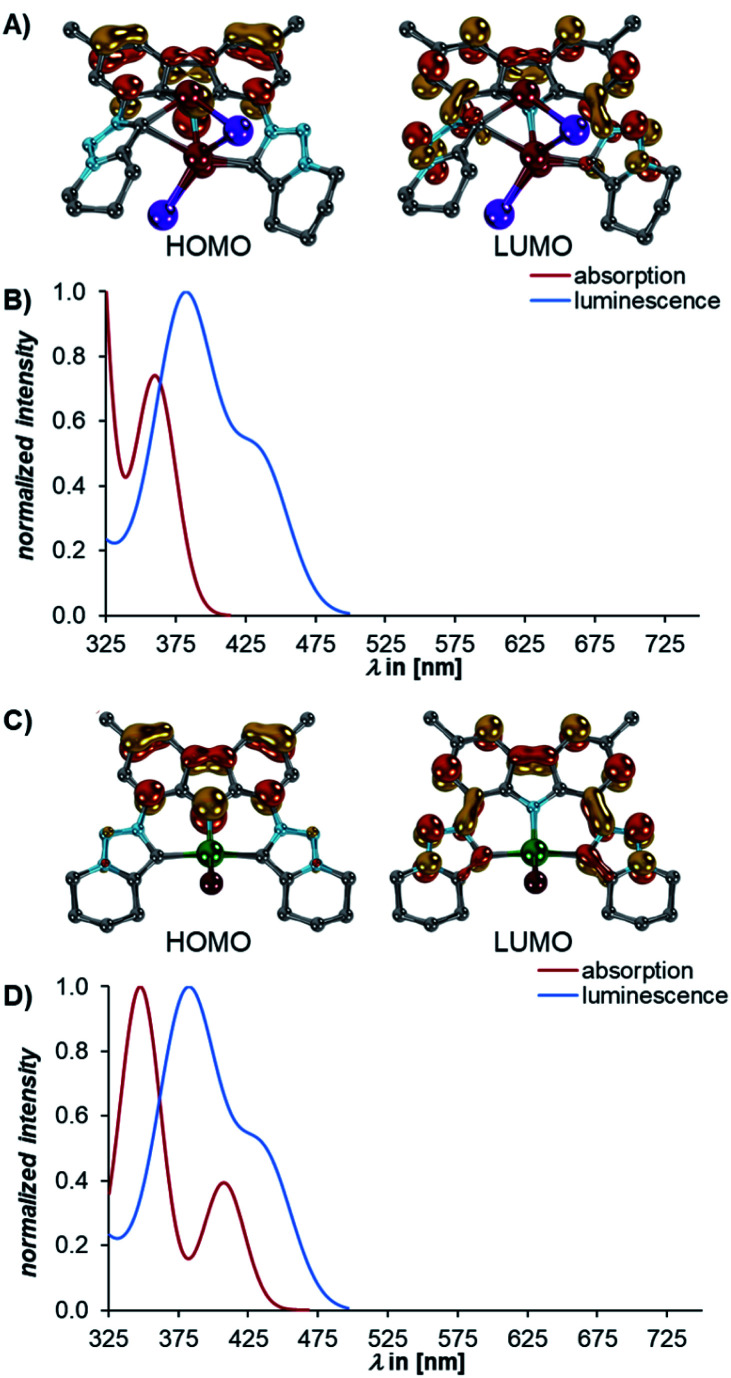
The dominant absorptions and concomitant excitations to the S_1_ states of **Li5** and **MgBr5** belong to the HOMO → LUMO transitions (B and D, red). For both **Li5** and **MgBr5**, the HOMOs (left) are located at the carbazole, whereas the LUMOs (right) are mainly localized on both MIC units (A and C). For both **Li5** and **MgBr5**, the luminescence from the S_1_ states is bright (**Li5**, B and **MgBr5**, D, blue). Orbitals were obtained at the TD-DFT(SMD=C_6_H_6_)/def2-TZVPP//B3LYP-D3(BJ)/def2-SVP level of theory, whereas the luminescence spectra were obtained at the STEOM-CCSD(SMD=C_6_H_6_)/def2-TZVPP//B3LYP-D3(BJ)/def2-SVP level of theory. Hydrogen atoms are omitted for clarity.

A bright transition to the S_1_ state **Li5truncated** (*f*^osc^ = 0.17, ^calcd^*λ*^abs^ = 338 nm, ^exp^*λ*^abs^ = 402 nm) was also predicted for **Li5** (Fig. 8B). This transition originates also from the ILCT from the carbazolido- to the MIC ligand (Fig. 8). The same is true for **MgBr5** (*f*^osc^ = 0.17, ^calcd^*λ*^abs^ = 407 nm, ^exp^*λ*^abs^ = 431 nm (Fig. 8D). Luminescence from the S_1_ state of the complexes **5** is bright (**Li5**, *f*^osc^ = 0.10, ^calcd^*λ*^em^ = 408 nm, ^exp^*λ*^em^ = 506 nm, **MgBr5***f*^osc^ = 0.20, ^calcd^*λ*^em^ = 435 nm, ^exp^*λ*^em^ = 482 nm, Fig. 8B and D, respectively) and in agreement with **5** being luminescent with quantum yields of 16% for **Li5** and 14% for **MgBr5**. The STEOM-CCSD calculations also reproduce the experimental Stokes shifts (**4calcd** = 0.31, **4exp** = 0.35; **Li5truncated calcd** = 0.40, **Li5exp** = 0.25; and **MgBr5calcd** = 0.20, **MgBr5exp** = 0.30 eV).

**Fig. 9:**
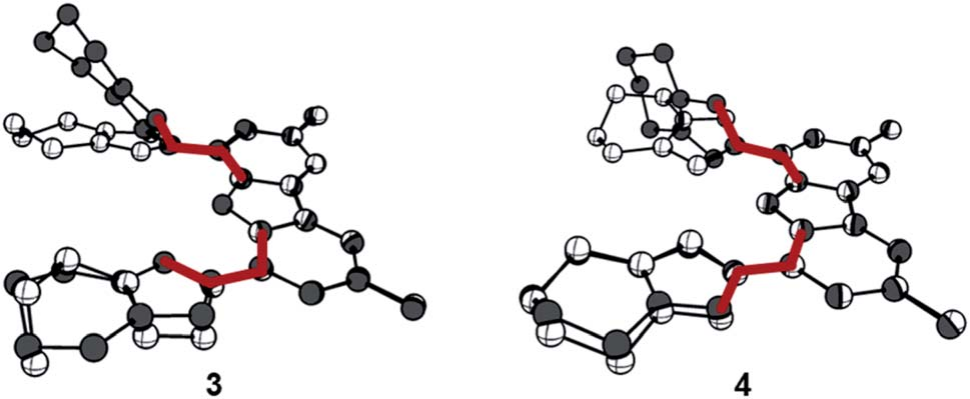
The distortions in the excited states of the proligand **3** (left) and **4** (right) visualized by superposition of the S_0_ (white) and S_1_ (grey) states. ^*t*^Bu groups are truncated to methyl groups, and hydrogen atoms are omitted for clarity. Dihedral angles (∠_C–C–N–N_) are highlighted in red.

Considering the vastly differing luminescent properties of **3**, **4**, and complexes **5**, we evaluated the geometries of the first S_1_ excited states. The proligand **3** is distorted in the relaxed S_1_ state, in which a triazolium group bends out of plane (Fig. 9, left). This means that the excitation is followed by a change of the mean value of the C–C–N–N dihedral angles before and after excitation (Δ∠_C–C–N–N_) between the carbazole and the triazolium unit of 90° (S_0_: ∠_C–C–N–N_ = 10° and S_1_: ∠_C–C–N–N_ = 100°). A comparable degree of distortion was also derived from the root-mean-square deviation for the change of the positions of all atoms (RMSD_S0→S1_; Fig. S38†).

**Fig. 10:**
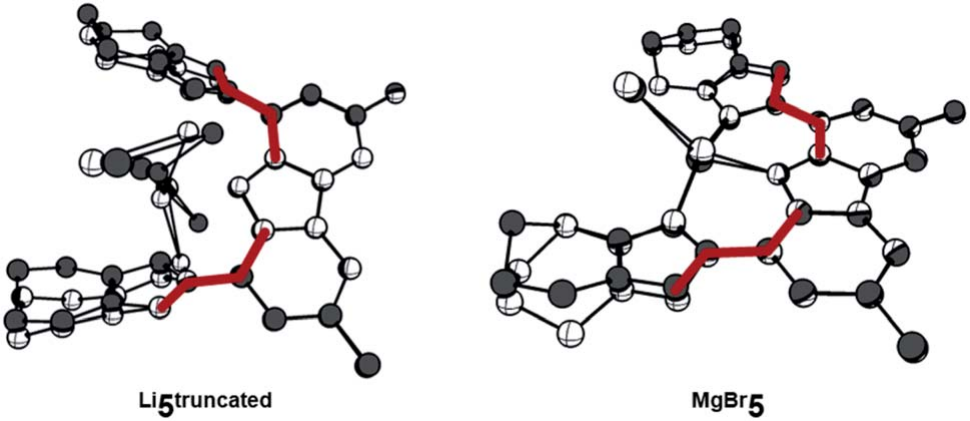
The metal prevents the distortions due to excitation in case of **Li5truncated** (left) and **MgBr5** (right), as visualized by the superposition of the S_0_ (white) and S_1_ (grey) states. ^*t*^Bu groups are truncated to methyl groups, and hydrogen atoms are omitted for clarity. Dihedral angles (∠_C–C–N–N_) are highlighted in red.

Upon the photoexcitation of **4**, a MIC group bends out of plane as well (Fig. 9, right) with Δ∠_C–C–N–N_ = 42° (S_0_: ∠_C–C–N–N_ = 177° and S_1_: ∠_C–C–N–N_ = 135°). **Li5** essentially retains the ground-state geometry in the relaxed S_1_ state, due to the fact that the coordinated metal ions lock the MIC ligands in place (Fig. 10, left). Throughout the excitation, the Δ∠_C–C–N–N_ changes only by 7° (S_0_: ∠_C–C–N–N_ = 139° and S_1_: ∠_C–C–N–N_ = 146°).

**Fig. 11:**
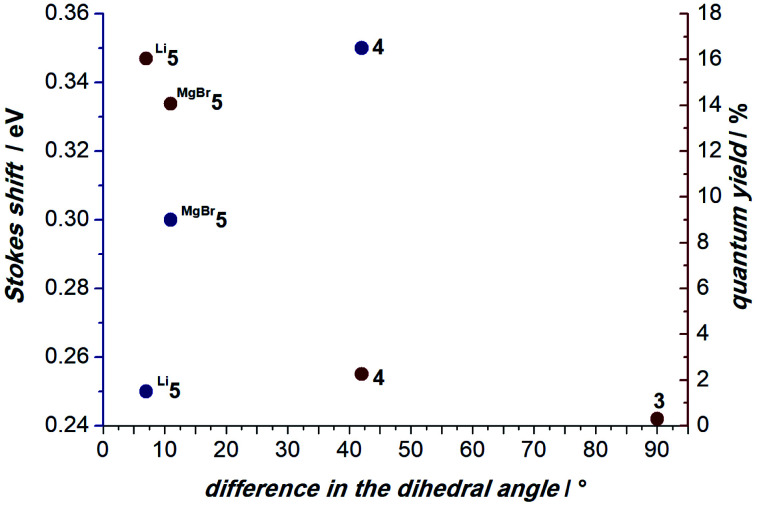
The excited state distortions, as expressed by the change of the mean value of the Δ∠_C–C–N–N_ dihedral angles between the carbazole and the MICs, correlate with the Stokes shifts (blue circles) and quantum yields (red circles) of compounds **3**, **4**, **Li5** and **MgBr5**. No Stokes shift is available for non-emissive **3**.

The anchoring effect of the metal is also evident for **MgBr5**, which retains a pseudo-square-planar coordination around the magnesium ion (Fig. 10, right). Here, the Δ∠_C–C–N–N_ is 11° (S_0_: ∠_C–C–N–N_ = 177° and S_1_ ∠_C–C–N–N_ = 166°). Intriguingly, the computationally determined degree of distortion in the excited S_1_ state correlates well with the experimental Stokes shifts and quantum yields (Fig. 11). For instance, **4** shows a large Stokes shift of Δ*E* = 0.35 eV and Δ∠_C–C–N–N_ = 42°, while **Li5** exhibits a Stokes shift of Δ*E* = 0.25 eV and Δ∠_C–C–N–N_ = 7°.

Berlman suggested that chromophores, in which the ground and first excited states are planar, show high quantum yields,^119,120^ because molecular distortions and rotations in the relaxed excited state often lead to non-radiative relaxation.^121–123^ This rigidification or Restriction of Intramolecular Motions (RIM) principle is of course crucial for organic fluorophores including ubiquitous BODIPY.^124,125^ It has also been exploited in sensing and coordination chemistry as the Chelation-Enhanced Fluorescence (CHEF) effect, in which the ground and excited geometries of a molecule are locked into a planar conformation by coordinating metals.^126–132^

Accordingly, complexes with comparably small structural distortions in the excited state are more efficient emitters (**Li5** Δ∠_C–C–N–N_ = 7°, *Φ*^em^ = 16%; **MgBr5** Δ∠_C–C–N–N_ = 11°, *Φ*^em^ = 14%; **4** Δ∠_C–C–N–N_ = 42°, *Φ*^em^ = 2%; **3** Δ∠_C–C–N–N_ = 90°, non-emissive). Therefore, we conclude that the structural relaxation of the excited S_1_ state quenches the luminescence. In contrast, the alkali- and earth-alkali metals lock the conformation and, hence, allow for bright luminescence with comparably small Stokes shifts.

## Conclusions

We report on the synthesis of an *N*-fused CNC pincer proligand composed of carbazole and two triazolium units. The synthetic approach, scalable to multigram quantities, avoids the use of hazardous *tert*-butylhypochloride, which had found use for related ligand systems. The proligand undergoes single deprotonation to afford a rare “naked” amide, which is air-stable due to intramolecular hydrogen-type bonding interactions. Triple deprotonation by a lithium base affords a chelated, binuclear lithium complex, which undergoes transmetalation with magnesium. Photophysical investigations show that the *s*-block complexes excel with luminescence quantum yields of up to 16% at ambient temperature and in solution, whereas the pro- and mono-deprotonated ligands are essentially non-luminescent. Detailed quantum-chemical calculations helped to rationalize the luminescent properties with an Intra-Ligand- Charge-Transfer (ILCT) from the carbazolide to the mesoionic carbenes. (Earth-)alkali metals prevent the distortion of the ligand following excitation and, in turn, enable bright luminescence in the blue to green region of the spectrum.

## Author contributions

PP and SH prepared the compound **4**, PP prepared as well complexes **Li5**, **MgBr5** and ran the computations, CMS supported by TU performed the photochemical studies, FAW and ND synthesized the compounds **1**, **2** and **3**, RHI performed the XRD analysis of **3**, SH performed the XRD analysis of **4**, BM performed the XRD analysis of **Li5**, AG contributed to the synthesis of **Li5** and **MgBr5**, MZ performed the DOSY experiments, DMG, SH and DM coordinated the project, DM conceived the idea. The manuscript was proof-read and approved by all authors.

## Conflicts of interest

There are no conflicts to declare.

## Supplementary Material

SC-012-D1SC00846C-s001

SC-012-D1SC00846C-s002

## References

[cit1] Promarak V., Ichikawa M., Sudyoadsuk T., Saengsuwan S., Jungsuttiwong S., Keawin T. (2008). Thin Solid Films.

[cit2] Wang Z. S., Koumura N., Cui Y., Takahashi M., Sekiguchi H., Mori A., Kubo T., Furube A., Hara K. (2008). Chem. Mater..

[cit3] Tang J., Hua J. L., Wu W. J., Li J., Jin Z. G., Long Y. T., Tian H. (2010). Energy Environ. Sci..

[cit4] Luo J., Zhang J. (2016). ACS Catal..

[cit5] Al Mousawi A., Dumur F., Garra P., Toufaily J., Hamieh T., Graff B., Gigmes D., Fouassier J. P., Lalevée J. (2017). Macromolecules.

[cit6] Huang Z., Gu Y., Liu X., Zhang L., Cheng Z., Zhu X. (2017). Macromol. Rapid Commun..

[cit7] Dumur F. (2020). Eur. Polym. J..

[cit8] Brunner K., van Dijken A., Börner H., Bastiaansen J. J. A. M., Kiggen N. M. M., Langeveld B. M. W. (2004). J. Am. Chem. Soc..

[cit9] Wex B., Kaafarani B. R. (2017). J. Mater. Chem..

[cit10] Fleetham T., Li G., Wen L., Li J. (2014). Adv. Mater..

[cit11] Fleetham T., Li G., Li J. (2017). Adv. Mater..

[cit12] Uoyama H., Goushi K., Shizu K., Nomura H., Adachi C. (2012). Nature.

[cit13] Kotchapradist P., Prachumrak N., Tarsang R., Jungsuttiwong S., Keawin T., Sudyoadsuk T., Promarak V. (2013). J. Mater. Chem..

[cit14] Gao Z., Wang Z. M., Shan T., Liu Y. L., Shen F. Z., Pan Y. Y., Zhang H. H., He X., Lu P., Yang B., Ma Y. G. (2014). Org. Electron..

[cit15] Albrecht K., Matsuoka K., Fujita K., Yamamoto K. (2015). Angew. Chem., Int. Ed..

[cit16] Zhang Q., Li J., Shizu K., Huang S., Hirata S., Miyazaki H., Adachi C. (2012). J. Am. Chem. Soc..

[cit17] Furukawa T., Nakanotani H., Inoue M., Adachi C. (2015). Sci. Rep..

[cit18] Kaji H., Suzuki H., Fukushima T., Shizu K., Suzuki K., Kubo S., Komino T., Oiwa H., Suzuki F., Wakamiya A., Murata Y., Adachi C. (2015). Nat. Commun..

[cit19] Di D., Romanov A. S., Yang L., Richter J. M., Rivett J. P., Jones S., Thomas T. H., Abdi Jalebi M., Friend R. H., Linnolahti M., Bochmann M., Credgington D. (2017). Science.

[cit20] Lee Y. H., Park S., Oh J., Woo S. J., Kumar A., Kim J. J., Jung J., Yoo S., Lee M. H. (2018). Adv. Opt. Mater..

[cit21] Liu Y. C., Li C. S., Ren Z. J., Yan S. K., Bryce M. R. (2018). Nat. Rev. Mater..

[cit22] Hamze R., Peltier J. L., Sylvinson D., Jung M., Cardenas J., Haiges R., Soleilhavoup M., Jazzar R., Djurovich P. I., Bertrand G., Thompson M. E. (2019). Science.

[cit23] Hamze R., Shi S., Kapper S. C., Muthiah Ravinson D. S., Estergreen L., Jung M. C., Tadle A. C., Haiges R., Djurovich P. I., Peltier J. L., Jazzar R., Bertrand G., Bradforth S. E., Thompson M. E. (2019). J. Am. Chem. Soc..

[cit24] Shi S., Jung M. C., Coburn C., Tadle A., Sylvinson M. R. D., Djurovich P. I., Forrest S. R., Thompson M. E. (2019). J. Am. Chem. Soc..

[cit25] Conaghan P. J., Matthews C. S. B., Chotard F., Jones S. T. E., Greenham N. C., Bochmann M., Credgington D., Romanov A. S. (2020). Nat. Commun..

[cit26] Feng H. T., Zeng J., Yin P. A., Wang X. D., Peng Q., Zhao Z., Lam J. W. Y., Tang B. Z. (2020). Nat. Commun..

[cit27] Romanov A. S., Chotard F., Rashid J., Bochmann M. (2019). Dalton Trans..

[cit28] Feng J., Taffet E. J., Reponen A. P. M., Romanov A. S., Olivier Y., Lemaur V., Yang L., Linnolahti M., Bochmann M., Beljonne D., Credgington D. (2020). Chem. Mater..

[cit29] Hall C. R., Romanov A. S., Bochmann M., Meech S. R. (2018). J. Phys. Chem. Lett..

[cit30] Romanov A. S., Yang L., Jones S. T. E., Di D., Morley O. J., Drummond B. H., Reponen A. P. M., Linnolahti M., Credgington D., Bochmann M. (2019). Chem. Mater..

[cit31] Romanov A. S., Jones S. T. E., Yang L., Conaghan P., Di D. W., Linnolahti M., Credgington D., Bochmann M. (2018). Adv. Opt. Mater..

[cit32] Gernert M., Balles-Wolf L., Kerner F., Müller U., Schmiedel A., Holzapfel M., Marian C. M., Pflaum J., Lambert C., Steffen A. (2020). J. Am. Chem. Soc..

[cit33] (c) Nolan S. P., N-Heterocyclic Carbenes: Effective Tools for Organometallic Synthesis, Wiley-VCH, Weinheim, 2014

[cit34] Foller J., Marian C. M. (2017). J. Phys. Chem. Lett..

[cit35] Taffet E. J., Olivier Y., Lam F., Beljonne D., Scholes G. D. (2018). J. Phys. Chem. Lett..

[cit36] Thompson S., Eng J., Penfold T. J. (2018). J. Chem. Phys..

[cit37] Gomez-Bombarelli R., Aguilera-Iparraguirre J., Hirzel T. D., Duvenaud D., Maclaurin D., Blood-Forsythe M. A., Chae H. S., Einzinger M., Ha D. G., Wu T., Markopoulos G., Jeon S., Kang H., Miyazaki H., Numata M., Kim S., Huang W., Hong S. I., Baldo M., Adams R. P., Aspuru-Guzik A. (2016). Nat. Mater..

[cit38] Peris E. (2018). Chem. Rev..

[cit39] Poyatos M., Mata J. A., Peris E. (2009). Chem. Rev..

[cit40] Peris E., Crabtree R. H. (2004). Coord. Chem. Rev..

[cit41] Röther A., Kretschmer R. (2020). J. Organomet. Chem..

[cit42] Munz D. (2018). Chem. Sci..

[cit43] Grünwald A., Munz D. (2018). J. Organomet. Chem..

[cit44] Grünwald A., Orth N., Scheurer A., Heinemann F. W., Pothig A., Munz D. (2018). Angew. Chem., Int. Ed..

[cit45] Bennington M. S., Feltham H. L., Buxton Z. J., White N. G., Brooker S. (2017). Dalton Trans..

[cit46] Gee H. C., Lee C. H., Jeong Y. H., Jang W. D. (2011). Chem. Commun..

[cit47] Aihara H., Jaquinod L., Nurco D. J., Smith K. M. (2001). Angew. Chem., Int. Ed..

[cit48] Inoue M., Nakada M. (2007). Heterocycles.

[cit49] Arnold L., Norouzi-Arasi H., Wagner M., Enkelmann V., Mullen K. (2011). Chem. Commun..

[cit50] Nath M., Huffman J. C., Zaleski J. M. (2003). Chem. Commun..

[cit51] Pryjomska-Ray I., Zornik D., Patzel M., Krause K. B., Grubert L., Braun-Cula B., Hecht S., Limberg C. (2018). Chem.–Eur. J..

[cit52] Schulze B., Friebe C., Jäger M., Görls H., Birckner E., Winter A., Schubert U. S. (2017). Organometallics.

[cit53] Zhang X., Zhang L. Y., Wang J. Y., Dai F. R., Chen Z. N. (2020). J. Mater. Chem..

[cit54] Inoue M., Suzuki T., Nakada M. (2003). J. Am. Chem. Soc..

[cit55] Gaunt J. A., Gibson V. C., Haynes A., Spitzmesser S. K., White A. J. P., Williams D. J. (2004). Organometallics.

[cit56] Britovsek G. J. P., Gibson V. C., Hoarau O. D., Spitzmesser S. K., White A. J. P., Williams D. J. (2003). Inorg. Chem..

[cit57] Gibson V. C., Spitzmesser S. K., White A. J. P., Williams D. J. (2003). Dalton Trans..

[cit58] Kleinhans G., Hansmann M. M., Guisado-Barrios G., Liles D. C., Bertrand G., Bezuidenhout D. I. (2016). J. Am. Chem. Soc..

[cit59] Bezuidenhout D. I., Kleinhans G., Guisado-Barrios G., Liles D. C., Ung G., Bertrand G. (2014). Chem. Commun..

[cit60] Kleinhans G., Guisado-Barrios G., Peris E., Bezuidenhout D. I. (2018). Polyhedron.

[cit61] Jerabek P., Vondung L., Schwerdtfeger P. (2018). Chem.–Eur. J..

[cit62] Kleinhans G., Chan A. K. W., Leung M. Y., Liles D. C., Fernandes M. A., Yam V. W. W., Fernández I., Bezuidenhout D. I. (2020). Chem.–Eur. J..

[cit63] Moser M., Wucher B., Kunz D., Rominger F. (2007). Organometallics.

[cit64] Jürgens E., Buys K. N., Schmidt A. T., Furfari S. K., Cole M. L., Moser M., Rominger F., Kunz D. (2016). New J. Chem..

[cit65] Seyboldt A., Wucher B., Hohnstein S., Eichele K., Rominger F., Tornroos K. W., Kunz D. (2015). Organometallics.

[cit66] Maulbetsch T., Jürgens E., Kunz D. (2020). Chem.–Eur. J..

[cit67] Jürgens E., Back O., Mayer J. J., Heinze K., Kunz D. (2016). Z. Naturforsch., B: J. Chem. Sci..

[cit68] Tian Y. Y., Jürgens E., Mill K., Jordan R., Maulbetsch T., Kunz D. (2019). ChemCatChem.

[cit69] Sau S. C., Hota P. K., Mandal S. K., Soleilhavoup M., Bertrand G. (2020). Chem. Soc. Rev..

[cit70] Vivancos Á., Segarra C., Albrecht M. (2018). Chem. Rev..

[cit71] Schuster O., Yang L., Raubenheimer H. G., Albrecht M. (2009). Chem. Rev..

[cit72] Ghadwal R. S. (2016). Dalton Trans..

[cit73] Huynh H. V., in The Organometallic Chemistry of N-heterocyclic Carbenes, 2017, p. 293

[cit74] Schweinfurth D., Hettmanczyk L., Suntrup L., Sarkar B. (2017). Z. Anorg. Allg. Chem..

[cit75] Guisado-Barrios G., Soleilhavoup M., Bertrand G. (2018). Acc. Chem. Res..

[cit76] Huynh H. V. (2018). Chem. Rev..

[cit77] Nelson D. J., Nolan S. P. (2013). Chem. Soc. Rev..

[cit78] Leigh V., Ghattas W., Lalrempuia R., Müller-Bunz H., Pryce M. T., Albrecht M. (2013). Inorg. Chem..

[cit79] Baschieri A., Monti F., Matteucci E., Mazzanti A., Barbieri A., Armaroli N., Sambri L. (2016). Inorg. Chem..

[cit80] Soellner J., Tenne M., Wagenblast G., Strassner T. (2016). Chem.–Eur. J..

[cit81] Sarkar B., Suntrup L. (2017). Angew. Chem., Int. Ed..

[cit82] Matteucci E., Monti F., Mazzoni R., Baschieri A., Bizzarri C., Sambri L. (2018). Inorg. Chem..

[cit83] Suntrup L., Stein F., Hermann G., Kleoff M., Kuss-Petermann M., Klein J., Wenger O. S., Tremblay J. C., Sarkar B. (2018). Inorg. Chem..

[cit84] Soellner J., Strassner T. (2018). Chem.–Eur. J..

[cit85] Soellner J., Strassner T. (2019). ChemPhotoChem.

[cit86] Brown D. G., Sanguantrakun N., Schulze B., Schubert U. S., Berlinguette C. P. (2012). J. Am. Chem. Soc..

[cit87] Bens T., Boden P., Di Martino-Fumo P., Beerhues J., Albold U., Sobottka S., Neuman N. I., Gerhards M., Sarkar B. (2020). Inorg. Chem..

[cit88] Messelberger J., Grünwald A., Pinter P., Hansmann M. M., Munz D. (2018). Chem. Sci..

[cit89] Pinter P., Munz D. (2020). J. Phys. Chem. A.

[cit90] Maulbetsch T., Kunz D. (2021). Angew. Chem., Int. Ed..

[cit91] Back O., Förster C., Basché T., Heinze K. (2019). Chem.–Eur. J..

[cit92] Liu Z., Salata O. V., Male N. (2002). Synth. Met..

[cit93] Feuerstein T. J., Goswami B., Rauthe P., Köppe R., Lebedkin S., Kappes M. M., Roesky P. W. (2019). Chem. Sci..

[cit94] Ribson R. D., Choi G., Hadt R. G., Agapie T. (2020). ACS Cent. Sci..

[cit95] Förster C., Heinze K. (2020). Chem. Soc. Rev..

[cit96] Tseng M. C., Cheng H. T., Shen M. J., Chu Y. H. (2011). Org. Lett..

[cit97] Krause L., Herbst-Irmer R., Sheldrick G. M., Stalke D. (2015). J. Appl. Crystallogr..

[cit98] Baltrun M., Watt F. A., Schoch R., Wölper C., Neuba A. G., Hohloch S. (2019). Dalton Trans..

[cit99] Hohloch S., Suntrup L., Sarkar B. (2016). Inorg. Chem. Front..

[cit100] Johnson K. R. D., Kamenz B. L., Hayes P. G. (2014). Organometallics.

[cit101] Esbak H., Behrens U. (2005). Z. Anorg. Allg. Chem..

[cit102] Lambert C., Hampel F., von Raguė Schleyer P. (1992). Angew. Chem., Int. Ed..

[cit103] Reetz M. T., Hutte S., Goddard R., Minet U. (1995). J. Chem. Soc., Chem. Commun..

[cit104] Ünal A., Ayın Ö. (2021). J. Cluster Sci..

[cit105] Raston C. L., Whitaker C. R., White A. H. (1988). J. Chem. Soc., Dalton Trans..

[cit106] Komagawa S., Usui S., Haywood J., Harford P. J., Wheatley A. E., Matsumoto Y., Hirano K., Takita R., Uchiyama M. (2012). Angew. Chem., Int. Ed..

[cit107] Fei Z., Scopelliti R., Dyson P. J. (2003). Inorg. Chem..

[cit108] Dias H. V. R., Jin W. C. (1997). J. Chem. Crystallogr..

[cit109] See the ESI† for synthetic details

[cit110] XRD data for a Mg complex with a much related ancillary ligand further suggests that **MgBr5** is a monomer

[cit111] Jacquemin D., Mennucci B., Adamo C. (2011). Phys. Chem. Chem. Phys..

[cit112] Adamo C., Jacquemin D. (2013). Chem. Soc. Rev..

[cit113] Laurent A. D., Jacquemin D. (2013). Int. J. Quantum Chem..

[cit114] Nooijen M., Bartlett R. J. (1997). J. Chem. Phys..

[cit115] Berraud-Pache R., Neese F., Bistoni G., Izsák R. (2020). J. Chem. Theory Comput..

[cit116] Izsák R. (2019). Wiley Interdiscip. Rev.: Comput. Mol. Sci..

[cit117] Riplinger C., Neese F. (2013). J. Chem. Phys..

[cit118] Riplinger C., Sandhoefer B., Hansen A., Neese F. (2013). J. Chem. Phys..

[cit119] Berlman I. B. (1970). J. Phys. Chem. A.

[cit120] Nijegorodov N. I., Downey W. S. (1994). J. Phys. Chem. A.

[cit121] Baryshnikov G., Minaev B., Agren H. (2017). Chem. Rev..

[cit122] Escudero D., Jacquemin D. (2015). Dalton Trans..

[cit123] Mei J., Leung N. L. C., Kwok R. T. K., Lam J. W. Y., Tang B. Z. (2015). Chem. Rev..

[cit124] Loudet A., Burgess K. (2007). Chem. Rev..

[cit125] Ulrich G., Ziessel R., Harriman A. (2008). Angew. Chem., Int. Ed..

[cit126] Huston M. E., Haider K. W., Czarnik A. W. (1988). J. Am. Chem. Soc..

[cit127] Akkaya E. U., Huston M. E., Czarnik A. W. (1990). J. Am. Chem. Soc..

[cit128] Vance D. H., Czarnik A. W. (1994). J. Am. Chem. Soc..

[cit129] Shults M. D., Pearce D. A., Imperiali B. (2003). J. Am. Chem. Soc..

[cit130] Kwon J. Y., Jang Y. J., Lee Y. J., Kim K. M., Seo M. S., Nam W., Yoon J. (2005). J. Am. Chem. Soc..

[cit131] Lee H. Y., Bae D. R., Park J. C., Song H., Han W. S., Jung J. H. (2009). Angew. Chem., Int. Ed..

[cit132] Sinha N., Stegemann L., Tan T. T. Y., Doltsinis N. L., Strassert C. A., Hahn F. E. (2017). Angew. Chem., Int. Ed..

