# Supplementary material for: Bright luminescent lithium and magnesium carbene complexes
Source: Chem Sci. 2021 Apr 15;12(21):7401–10. doi: 10.1039/d1sc00846c (PMC8171342; doi:10.1039/d1sc00846c)
Supplement: SC-012-D1SC00846C-s001 [file SC-012-D1SC00846C-s001.pdf]

Electronic Supplementary Material (ESI) for Chemical Science.
This journal is © The Royal Society of Chemistry 2021

Supplementary Information for

Bright Luminescent Lithium and Magnesium Carbene Complexes

Piermaria Pinter,[a,§] Christoph M. Schüßlbauer,[b,§] Fabian A. Watt,[c,§] Nicole Dickmann,[c] Regine Herbst-Irmer,[d] Bernd Morgenstern,[e] Annette Grünwald,[a,f] Tobias Ullrich,[b] Michael Zimmer,[g] Dirk M. Guldi,*[b] Stephan Hohloch,*[h] Dominik Munz*[a,f]

[a] *Piermaria Pinter, Prof. Dr. Dominik Munz
Department of Chemistry and Pharmacy, Inorganic and General Chemistry Friedrich-Alexander University Erlangen-Nürnberg Egerlandstr. 1, D-91058 Erlangen, Germany.*
[b] *Christoph M. Schüßlbauer, Tobias Ullrich, Prof. Dr. Dr. h. c. Dirk M. Guldi Department of Chemistry and Pharmacy Interdisciplinary Center of Molecular Materials (ICMM) Friedrich-Alexander University Erlangen-Nürnberg Egerlandstraße 3, D-91058 Erlangen, Germany*
[c] *Fabian A. Watt, Nicole Dickmann
Department of Chemistry, Inorganic Chemistry, Paderborn University
Warburger Straße 100, D-33098 Paderborn, Germany*
[d] *Dr. Regine Herbst-Irmer
University of Göttingen, Institute of Inorganic Chemistry, Tammannstraße 4, D-37077 Göttingen, Germany*
[e] *Dr. Bernd Morgenstern, Dr. Michael Zimmer
Inorganic Solid State Chemistry Saarland University, Campus C4.1, D-66123 Saarbrücken, Germany.*
[f] *Annette Grünwald, Prof. Dr. Dominik Munz
Inorganic Chemistry: Coordination Chemistry, Saarland University, Campus C4.1, D-66123 Saarbrücken, Germany.*
[g] *Dr. M. Zimmer
Inorganic and General Chemistry, Saarland University, Campus C4.1, D-66123 Saarbrücken, Germany.*
[h] *Prof. Dr. Stephan Hohloch
Institute of General, Inorganic and Theoretical Chemistry, University of Innsbruck, Innrain 80-82, A-6020 Innsbruck, Austria*
[§] Authors contributed equally.

Table of Contents

1. Synthetical Methods – General S2-S13
2. Photospectroscopic Investigations S14-S15
3. Diffusion NMR (DOSY) S17
4. X-ray Single Crystal Structure Determinations S19-S21
5. Computational Details – General S22-S28
6. XYZ Coordinates S29-S35
7. Supplementary References S36

1. Synthetical Methods – General

All synthetic work (except synthesis of the proligand **3**) was performed in a nitrogen-filled glovebox. ^{1}H, ^{7}Li and ^{13}C NMR spectra were recorded on Bruker AV-500, Ascent 700 and JEOL ECX 400 instruments. The chemical shifts δ are calculated in ppm; the solvent residual signals of incompletely deuterated solvent molecules were used as an internal reference for the ^{1}H NMR spectra and the carbon solvent signals for ^{13}C NMR spectral data or external standards (^{7}Li NMR was referenced to 1M LiCl in D_2O and ^{15}N NMR spectra to ammonia). Solvents were purified using a two-column solid-state purification system (Glass Contour System, Irvine, CA), transferred to the glovebox without exposure to air and stored over a mirror of potassium or activated molecular sieves, respectively. NMR solvents were obtained dry and packaged under argon and as well stored over a mirror of potassium or activated molecular sieves. 3,6-di-*tert*-butyl-carbazole-1,8-diazide and TBTA were synthesized according to the literature.[1, 2] IR spectra were recorded at room temperature under inert conditions using a Bruker Vertex 70 with ATR equipment. Elemental analyses were performed using Elementar vario microcube instruments (University of Paderborn and University of Saarland). Compound **1** was prepared following the procedure reported by Limberg, Hecht and co-workers.[3]

2: A solution of copper sulfate (40 mg, 0.25 mmol, 0.1 eq.) in water (2 mL) was added to a mixture of 3,6-di-*tert*-butyl-carbazole-1,8-diazide (867 mg, 2.4 mmol, 1 eq.), 6-chloro-hex-1-yne (641 mg, 0.67 mL, 5.5 mmol, 2.3 eq.), sodium ascorbate (125 mg, 0.6 mmol, 0.25 eq.) and tris[(1-benzyl-1*H*-1,2,3-triazol-4-yl)methyl]amine (TBTA, 133 mg, 0.25 mmol, 0.1 eq) in a mixture of dichloromethane (10 mL), *tert*-butanol (10 mL) and water (2 mL). The reaction mixture was stirred at room temperature under an atmosphere of argon for 16 h. The beige-colored suspension was filtered, and the precipitate washed with *n*-pentane (2 × 10 mL). The solid was extracted with dichloromethane (2 × 25 mL) and filtered. All volatiles were removed under reduced pressure and the off-white-colored solid was further dried *in vacuo* to yield the desired product in 74% (1.78 mmol, 1.06 g). **^{1}H NMR** ($CDCl_3$, 298 K, 700 MHz): 10.78 (s, 1H, carbazole-N*H*), 8.16 (d, *J* = 1.3 Hz, 2H, Aryl-*H*), 7.98 (s, 2H, triazole-*5H*), 7.59 (d, *J* = 1.3 Hz, 2H, Aryl-*H*), 3.64 (t, *J* = 6.3 Hz, 4H, -CH_2Cl), 2.92 (t, *J* = 7.4 Hz, 4H, CH_2), 1.96 (m, 8H, CH_2), 1.51 (s, 18H, C(CH_3)$_3$) ppm; **^{13}C NMR** ($CDCl_3$, 298 K, 175 MHz): 147.8 (Aryl-C), 143.4 (Aryl-C), 130.3 (Aryl-C), 129.4 (Aryl-C), 128.3 (Aryl-C), 125.8 (Aryl-C), 121.5 (Aryl-C), 119,0 (triazole-5C), 116.7 (Aryl-CH), 114.6 (Aryl-CH), 45.0 (-CH_2Cl), 35.1 ($C(CH_3)_3$), 32.1 (CH_2) 32.1 ($C(CH_3)_3$), 26.7 (CH_2), 25.1 (CH_2) ppm.

3: The synthetic protocol for the internal cyclisation was adapted from a literature known protocol to design 1,2,3-triazolium derived ionic liquids[4]: A mixture of **2** (975 mg, 1.64 mmol, 1 eq.) and potassium iodide (5.46 g, 32.9 mmol, 20 eq.) was heated in acetonitrile (50 mL) at 90 °C for 44 h. All volatiles were then removed under reduced pressure. The remaining solid was extracted with dichloromethane (3 x 50 mL) and filtered. The filtrate was concentrated to 20 mL and added dropwise to a stirred solution of diethylether (300 mL). The precipitate was filtered off and washed with diethylether (100 mL) and *n*-pentane (30 mL). The beige-colored solid was dried *in vacuo* to yield the desired product in 96% (1.57 mmol, 1.22 g). **^{1}H NMR** (CD_2Cl_2, 298 K, 300 MHz): 11.00 (s, 1H, carbazole-N*H*), 8.91 (s, 2H, triazole-*5H*), 8.43 (d, *J* = 1.3 Hz, 2H, Aryl-*H*), 7.78 (d, *J* = 1.3 Hz, 2H, Aryl-*H*), 4.79 (t, *J* = 6.1 Hz, 4H, N-

C*H*$_2$), 3.24 (t, *J* = 6.4 Hz, 4H, C*H*$_2$), 2.36 (pent, *J* = 6.0 Hz, 4H, C*H*$_2$), 2.15 (dt, *J* = 11.5, 6.0 Hz, 4H, C*H*$_2$), 1.50 (s, 18H, C(C*H*$_3$)$_3$) ppm. **^{13}C NMR** (CD_2Cl_2, 298 K, 125 MHz): 145.1 (Aryl-*C*), 142.6 (Aryl-*C*), 133.2 (Aryl-*C*), 129.8 (triazolium-*5C*), 126.4 (Aryl-*C*), 121.3 (Aryl-*C*H), 121.3 (Aryl-*C*H), 119.6 (Aryl-*C*), 50.7 (N-*C*H$_2$), 35.6 (*C*(CH$_3$)$_3$), 32.1 (C(*C*H$_3$)$_3$), 21.9 (*C*H$_2$), 21.4 (*C*H$_2$), 18.5 (*C*H$_2$) ppm; **^{15}N NMR** (DMSO$_{d6}$, 298 K, 125 MHz): 112.6 (NH), 151.9 (N-Aryl), 247.6 (N-CH$_2$), 335.1 (N$_2$). **HR-MS** (ESI+) calcd. for $[C_{32}H_{41}N_7]^{2+}$ 261.6712; found 261.6722. **Elemental analysis** calcd. for $C_{32}H_{41}N_7I_2$ C 49.43, H 5.32, N 12.61; found C 49.82, H. 5.30, N 12.88. **MP** 358 °C.

4: In a vial, 100 mg of **3** (0.13 mmol, 1 eq.) were suspended in 4 mL of THF and. LiHMDS (22 mg, 0.13 µmol, 1 eq.) was added. Immediately, the solution turned intense orange. The reaction mixture was stirred at room temperature for 2 hours and then filtered. The residue was washed with THF (2x 5 mL) then pentane (2x 20 mL) and then the orange solid was dried *in vacuo* to yield the desired product in 90% (0.13 mmol, 75 mg). **^{1}H NMR** (DMSO$_{d6}$, 298 K, 400 MHz): δ = 10.37 (s, 2H, CH$_{triazolium}$), 8.46 (s, 2H, ArH), 7.95 (m, 2H, ArH),4.71 (m, 4H, CH$_2$), 3.15 (m, 4H, CH$_2$), 2.21 (m, 4H, CH$_2$), 2.00 (m, 4H, CH$_2$), 1.47 (s, 18H, C(CH$_3$)$_3$) ppm. **^{13}C NMR** (DMSO$_{d6}$, 298 K, 101 MHz): δ = 142.6 (ArC), 139.5 (ArC), 136.5 (ArC), 128.3 (ArC), 127.5 (ArCH), 120.1 (ArC), 119.4 (ArCH), 112.8 (ArCH) 48.6 (N-CH$_2$), 34.5 (C(CH$_3$)$_3$), 32.0 (C(CH$_3$)$_3$), 21.2 (CH$_2$), (CH$_2$), 20.2 (CH$_2$), 17.8 (CH$_2$) ppm. **MP** 341 °C (decomposition). **Elemental analysis** calcd. for $C_{32}H_{41}N_7I_2$ 1.35LiI C 46.29, H 4.86, N 11.81; found C 46.13, H. 5.62, N 11.24.

^{Li}5: In a vial, 100 mg of **3** (0.13 mmol, 1 eq.) were suspended in 2 mL of Et_2O and LiHMDS (71 mg, 0.42 µmol, 3.2 eq.) was added. The reaction mixture was stirred at room temperature for 20 hours and then filtered. The product was precipitated by addition of pentane, washed with cold pentane (2x 20 mL) and the yellow-lime solid was dried *in vacuo* to yield the desired product in 39% (0.02 mmol, 47 mg). **^{1}H NMR** (DMSO$_{d6}$, 298 K, 400 MHz): δ = 8.10 (m, 2H, ArH), 8.00 (m, 2H, ArH), 4.51 (m, 4H, CH$_2$), 3.06 (m, 4H, CH$_2$), 2.10 (m, 4H, CH$_2$), 1.94 (m, 4H, CH$_2$), 1.46 (s, 18H, C(CH$_3$)$_3$) ppm. **^{13}C NMR** (DMSO$_{d6}$, 298 K, 101 MHz): δ = 187.1 (C$_{carbene}$), 143.4 (ArC), 142.7 (ArC), 135.6 (ArC), 127.1 (ArC), 126.9 (ArC), 115.4 (ArC), 112.2 (ArC), 65.0 (N-CH$_2$), 34.4 (C(CH$_3$)$_3$), 32.4 (C(CH$_3$)$_3$), 22.3 (CH$_2$), 20.0 (CH$_2$), 15.2 (CH$_2$) ppm. **^{7}Li NMR** (DMSO$_{d6}$, 298 K, 155.51 MHz): δ = -1.43 ppm. **MP** 223 °C (decomposition). **Elemental analysis** calcd. for $C_{32}H_{41}N_7I_2$ 5.22LiI C 31.14, H 3.12, N 8.00; found C 30.31, H. 4.18, N 7.07; after several recrystallizations calcd. for $C_{32}H_{41}N_7I_2$ 0.33LiI C 66.90, H 6.67, N 17.07; found C 67.08, H. 7.15, N 17.13.

^{MgBr}5: In a vial, 100 mg of **3** (64 µmol, 1 eq.) were suspended in 2 mL of Et_2O. LiHMDS (71 mg, 0.4 mmol, 3.3 eq.) was added and the reaction mixture was stirred for 6 hours at room temperature. Afterwards, the solution was filtered and all the volatiles were removed under vacuum. The residue was washed with pentane (2x 20 mL). Next, the solid was dissolved in 1 mL of Et_2O and 24 mg of dry $MgBr_2$ (71 µmol, 1.1 eq.) were added. The reaction was stirred for 4 h at room temperature, during which a yellow solid formed. The solid was separated, washed with Et_2O 2x 2 mL, pentane 2x 5 mL and dried *in vacuo* to yield the desired product in 66% (0.09 mmol, 53 mg). **^{1}H NMR** (DMSO$_{d6}$ 298 K, 400 MHz): δ = 8.26 (m, 2H, ArH), 8.17 (m, 2H, ArH), 4.57 (m, 4H, CH$_2$), 3.13 (m, 4H, CH$_2$), 2.14 (m, 4H, CH$_2$), 1.94

(m, 4H, CH_2), 1.47 (s, 18H, $C(CH_3)_3$) ppm; **^{13}C NMR** ($DMSO_{d6}$, 298 K,101 MHz): δ = 174.9 (s, $C_{carbene}$), 144.7 (ArC), 140.7 (ArC), 137.1 (ArC), 127.8 (ArC), 125.3 (ArC), 116.1 (ArC), 112.8 (ArC), 67.0 (CH_2), 46.8 (CH_2),34.4 ($C(CH_3)_3$), 32.1 ($C(CH_3)_3$), 23.0 (CH_2), 22.2 (CH_2), 19.8 (CH_2) ppm. **HR-MS** (ESI+) m/z: [M + H]+ calcd. for $C_{32}H_{41}BrN_7$ 602.26013; found 602.25701 (hydrolysis product). **MP** 189 °C (decomposition). **Elemental analysis** Despite of repeated attempts and our best efforts, the compound hydrolyzed during the elemental analysis. The NMR spectroscopic analysis (*cf* Fig S17) and the UV spectra confirm the absence of water in the sample. Calcd. for $C_{32}H_{38}BrMgN_7$ *1.76 H_2O 0.4 *Et_2O C 58.22, H 6.78, N 14.49; found C 58.55, H. 7.11, N 14.16.

Synthesis of ^{MgBr}5 with MeMgBr

Ligand **3** (30 mg, 0.04 mmol, 1.0 eq.) was suspended in 1 mL of diethylether and 1M MeMgBr in diethylether (13.5 µL, 0.12 mmol, 3.0 eq.) was added. The suspension turned immediately yellow. The solvent was removed *in vacuo* to give a yellow residue. The ^{1}H NMR spectroscopic analysis in DMSO-d_6 corroborated the clean and quantitative conversion to ^{MgBr}**5**. Note, no signals for a methyl complex (between 0 and –50 ppm) was found, whereas the *in-situ* reaction in deuterated benzene (Fig S19) revealed a singlet at 0.16 ppm, which can be assigned to free methane.

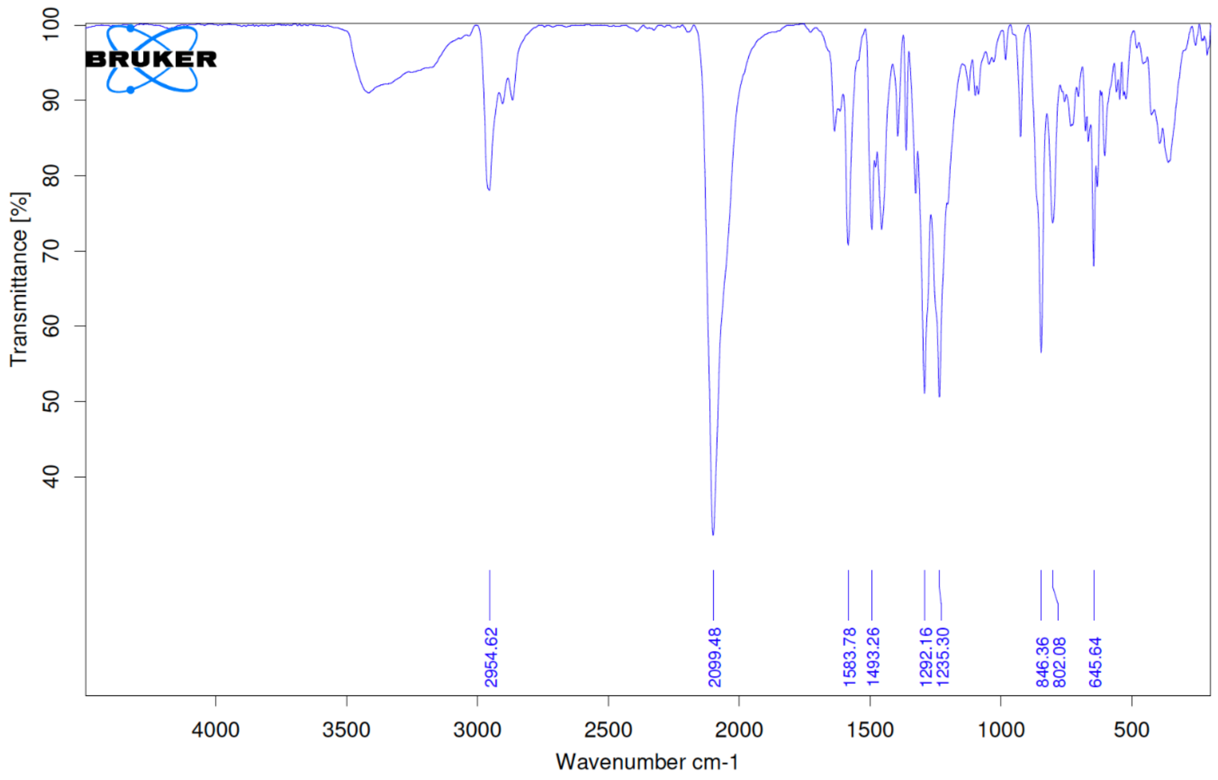

Fig. S1 IR spectrum of compound **1**.

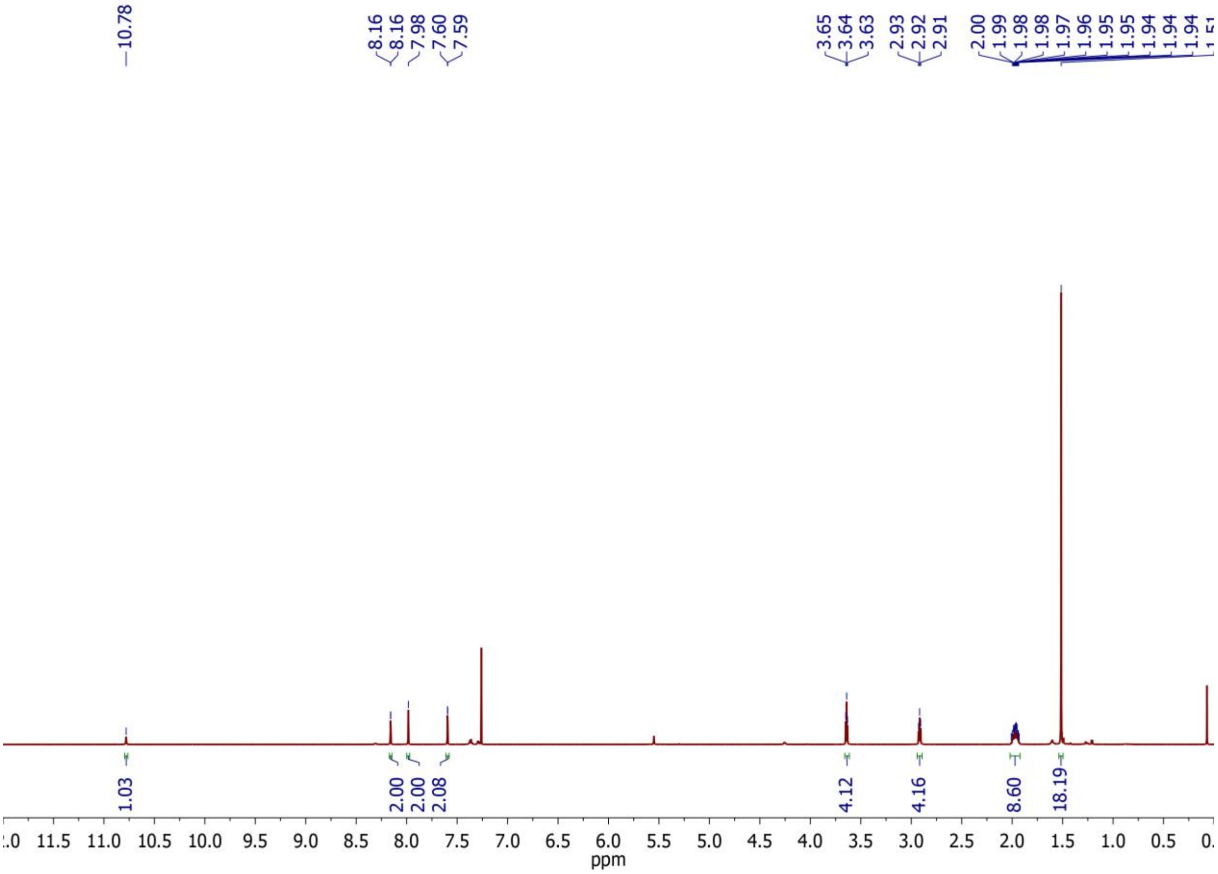

Fig. S2 ^{1}H NMR spectrum of compound **2**, measured in $CDCl_3$.

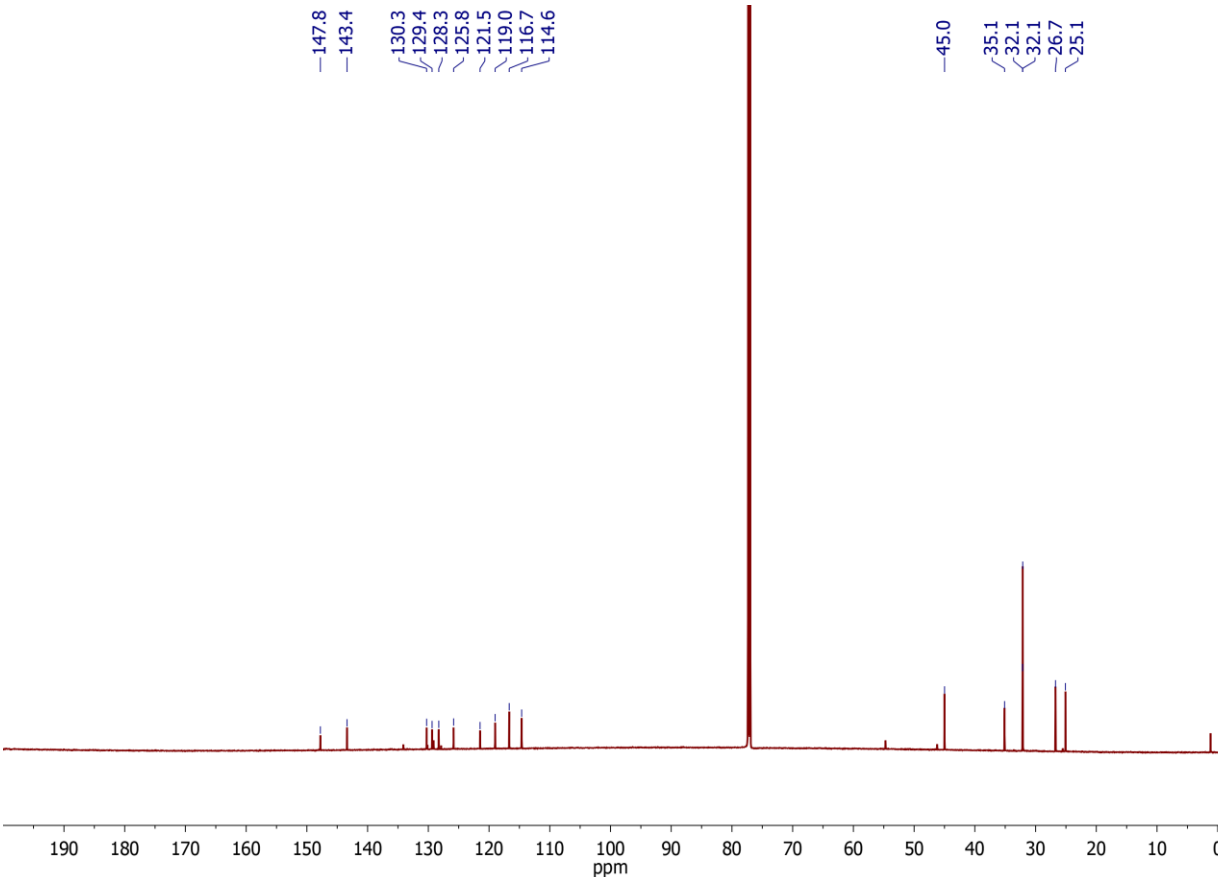

Fig. S3 ^{13}C NMR spectrum of compound **2**, measured in $CDCl_3$.

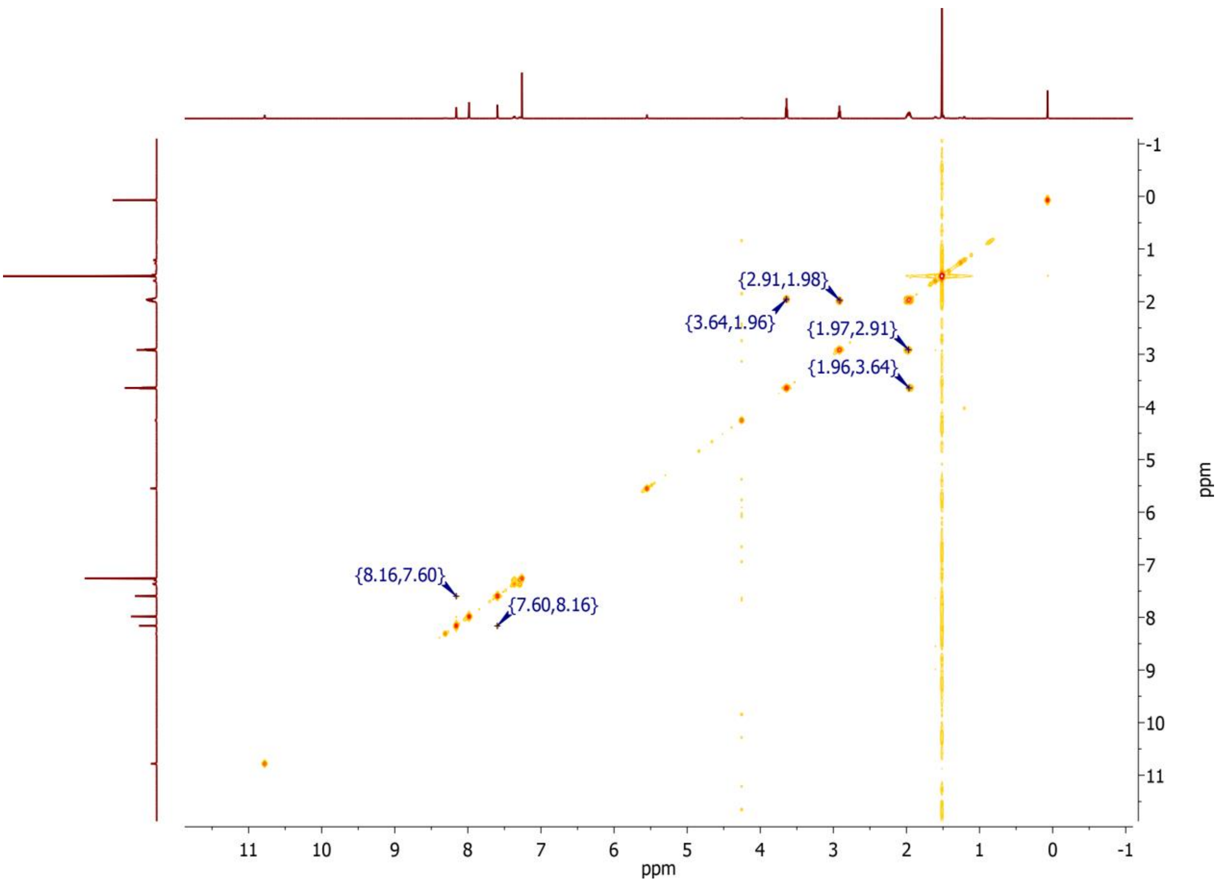

Fig S4 ^{1}H–^{1}H COSY spectrum of compound **2**, measured in $CDCl_3$.

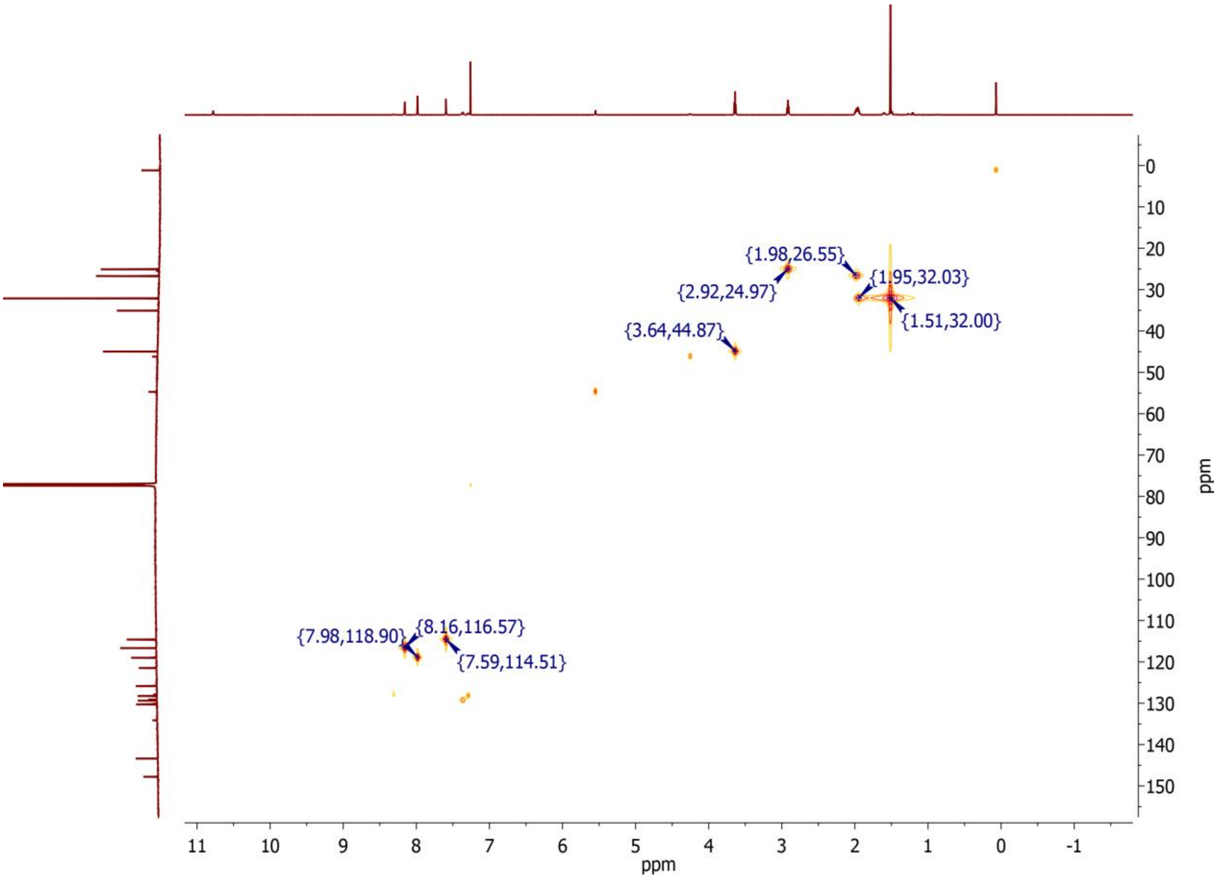

Fig. S5 ^{1}H–^{13}C HSQC spectrum of compound **2**, measured in $CDCl_3$.

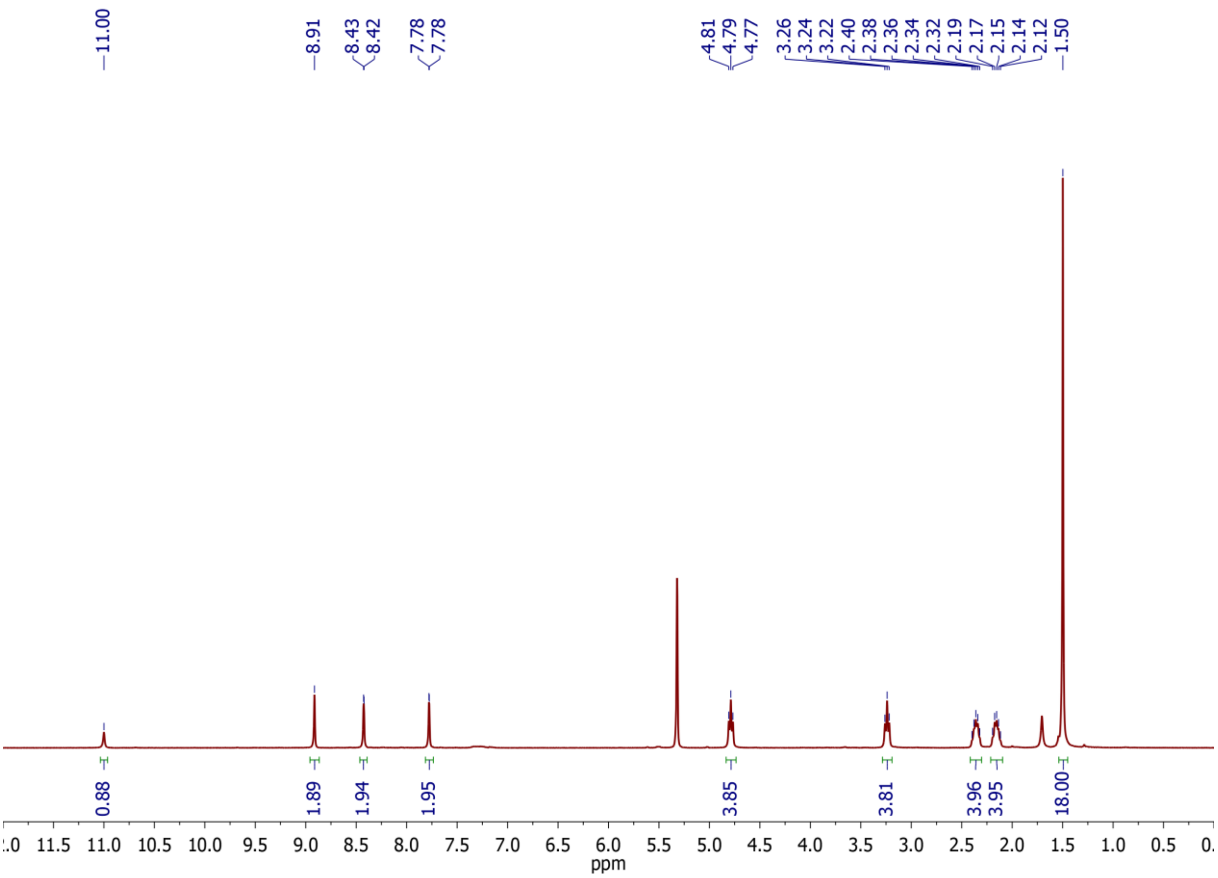

Fig. S6 ^{1}H NMR spectrum of compound **3**, measured in CD_2Cl_2.

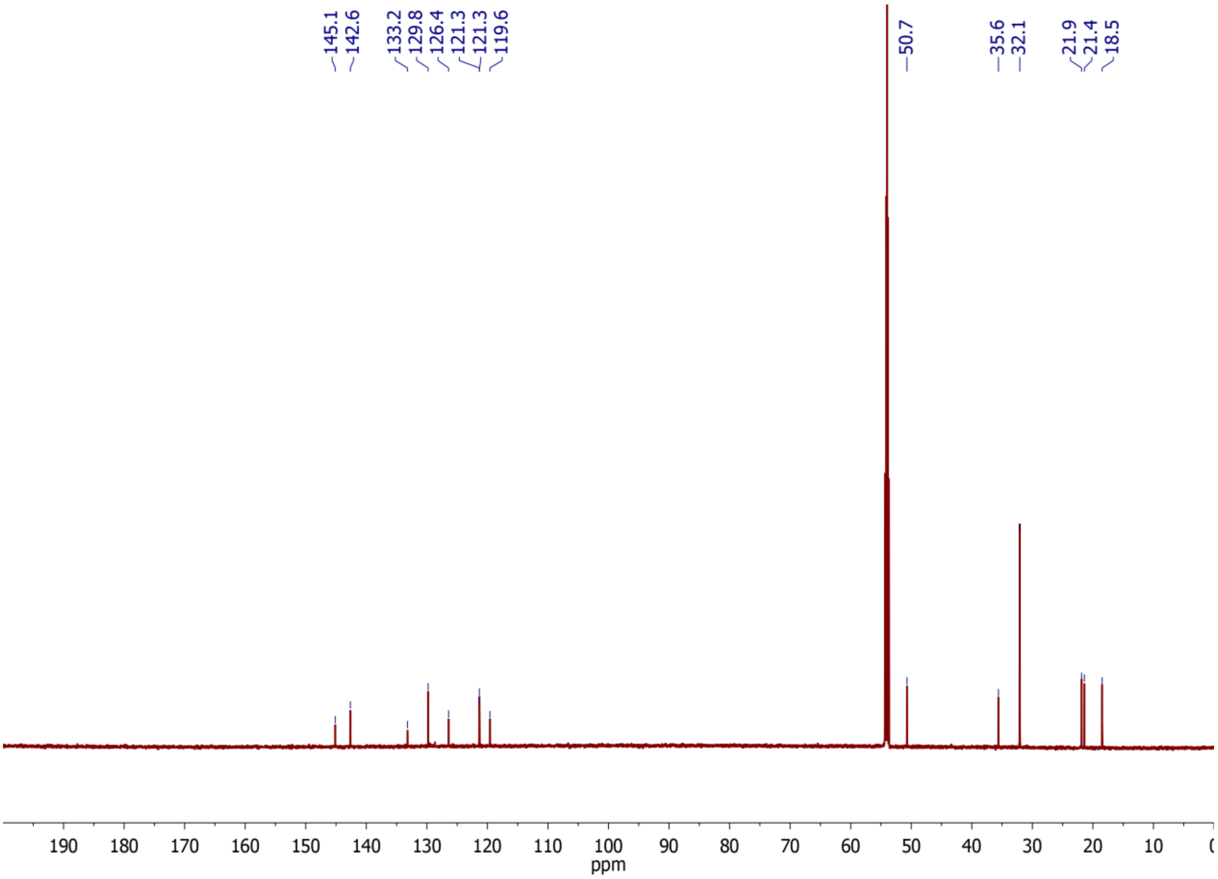

Fig. S7 ^{13}C NMR spectrum of compound **3**, measured in CD_2Cl_2.

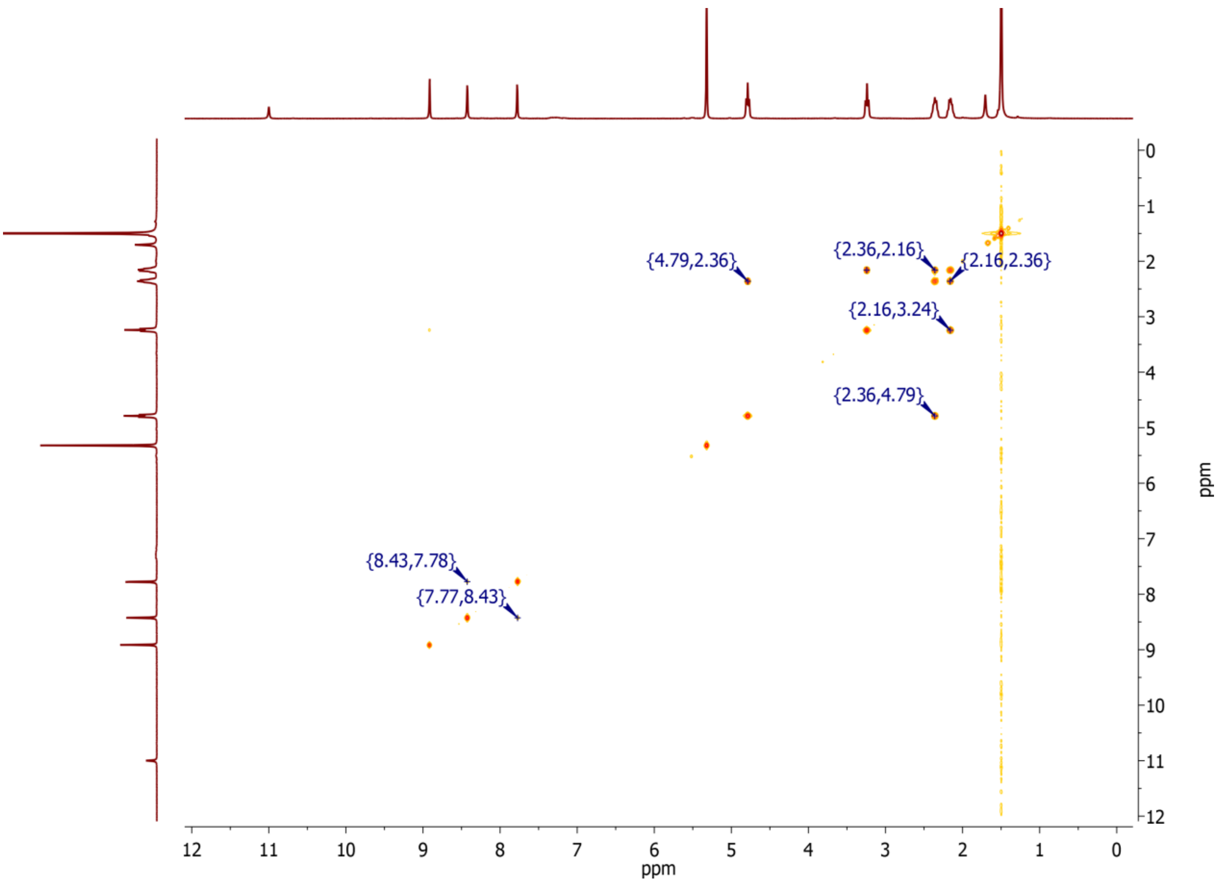

Fig. S8 ^{1}H–^{1}H COSY spectrum of compound **3**, measured in CD_2Cl_2.

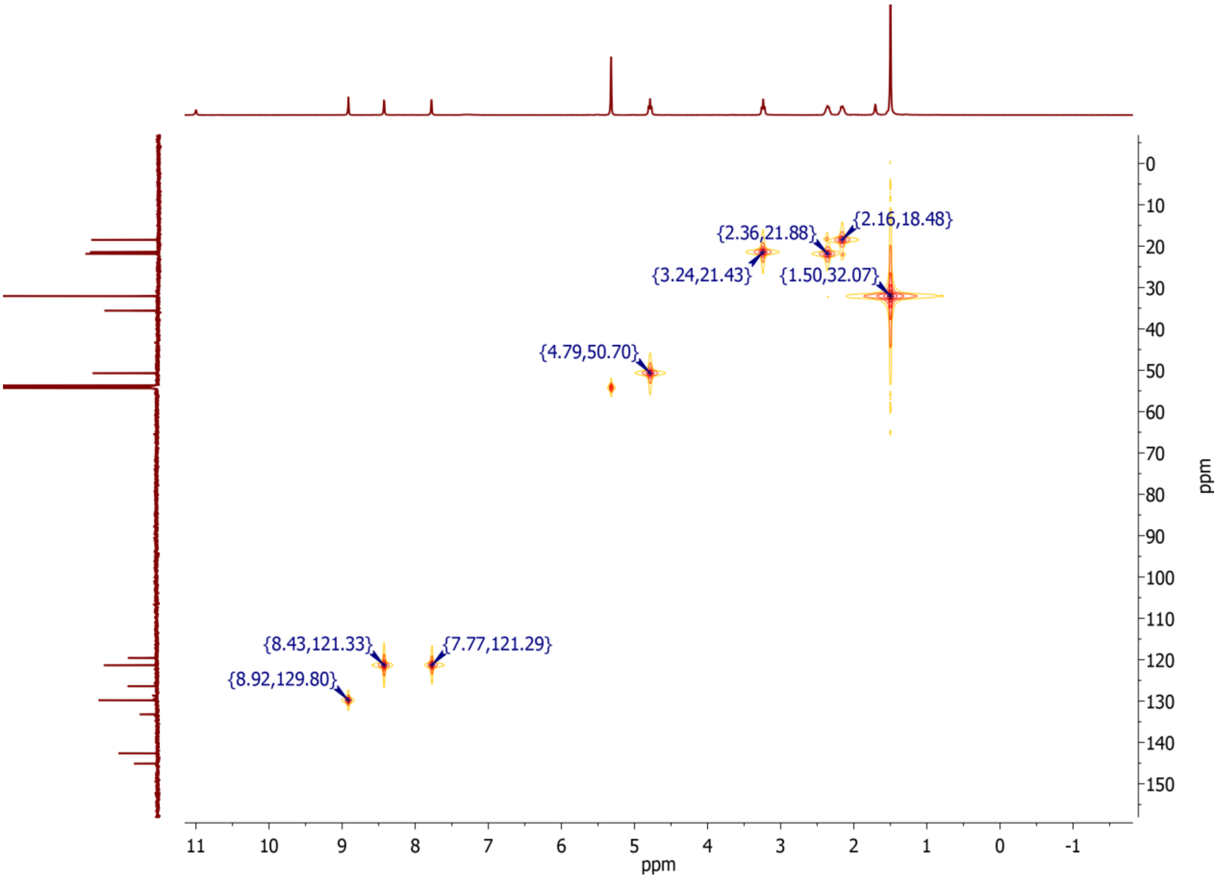

Fig. S9 ^{1}H–^{13}C HSQC spectrum of compound **3**, measured in CD_2Cl_2.

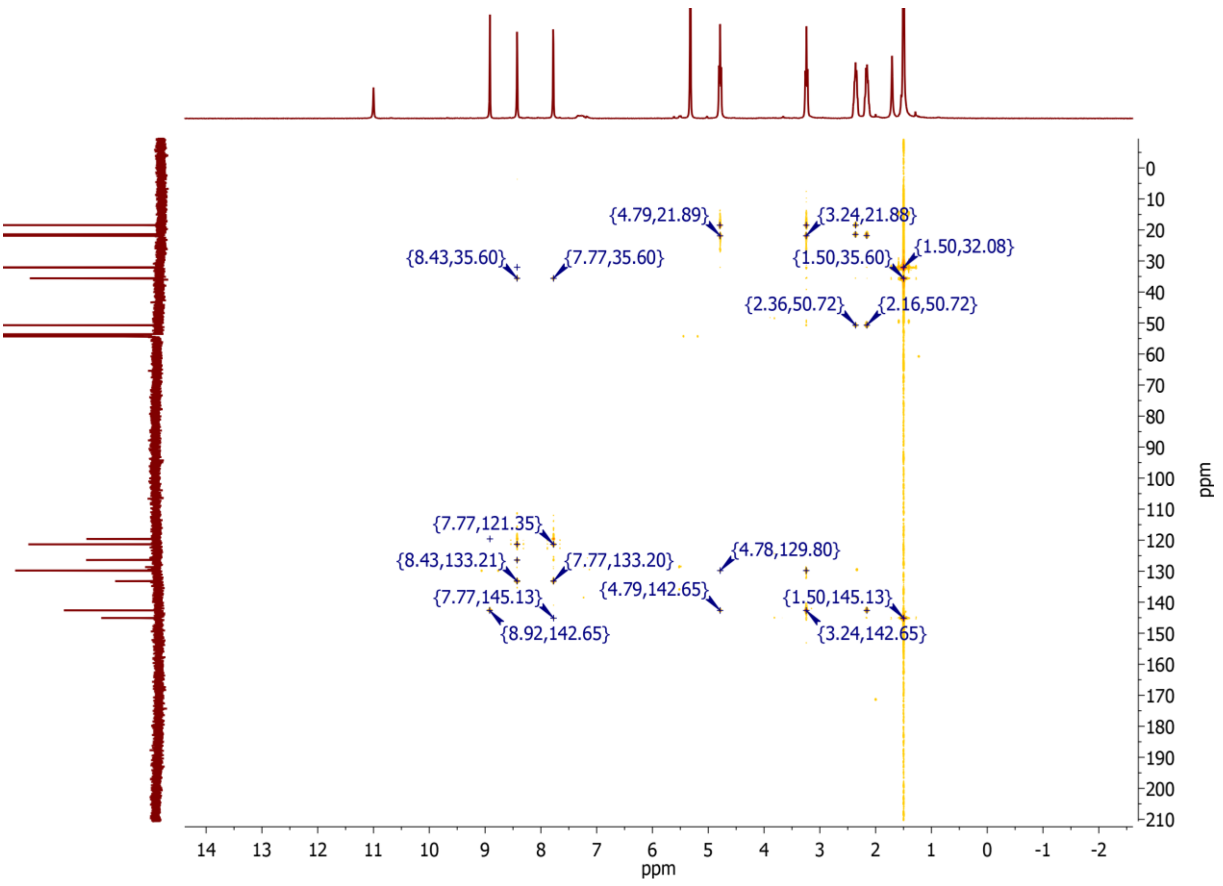

Fig. S10 ^{1}H–^{13}C HMBC spectrum of compound **3**, measured in CD_2Cl_2.

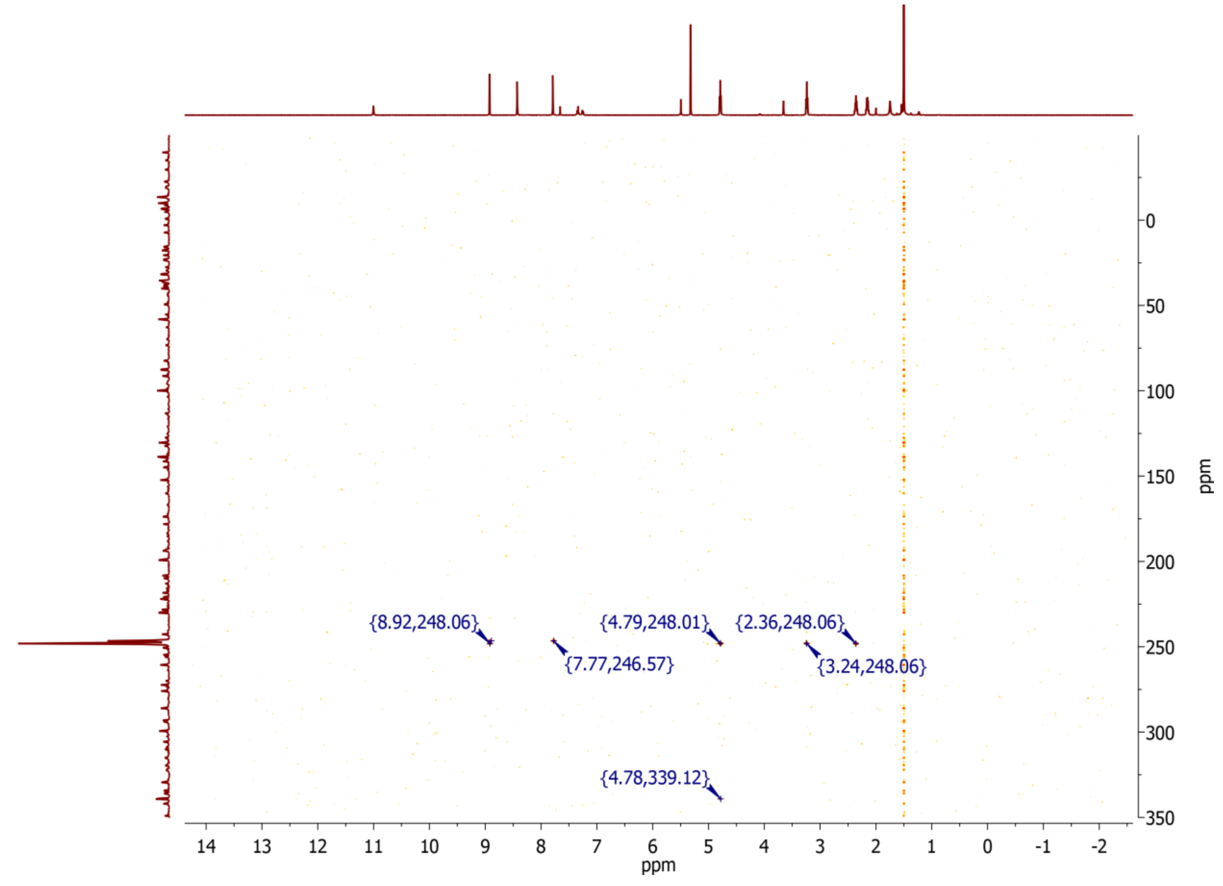

Fig. S11 ^{1}H–^{15}N HMBC spectrum of compound **3**, measured in CD_2Cl_2.

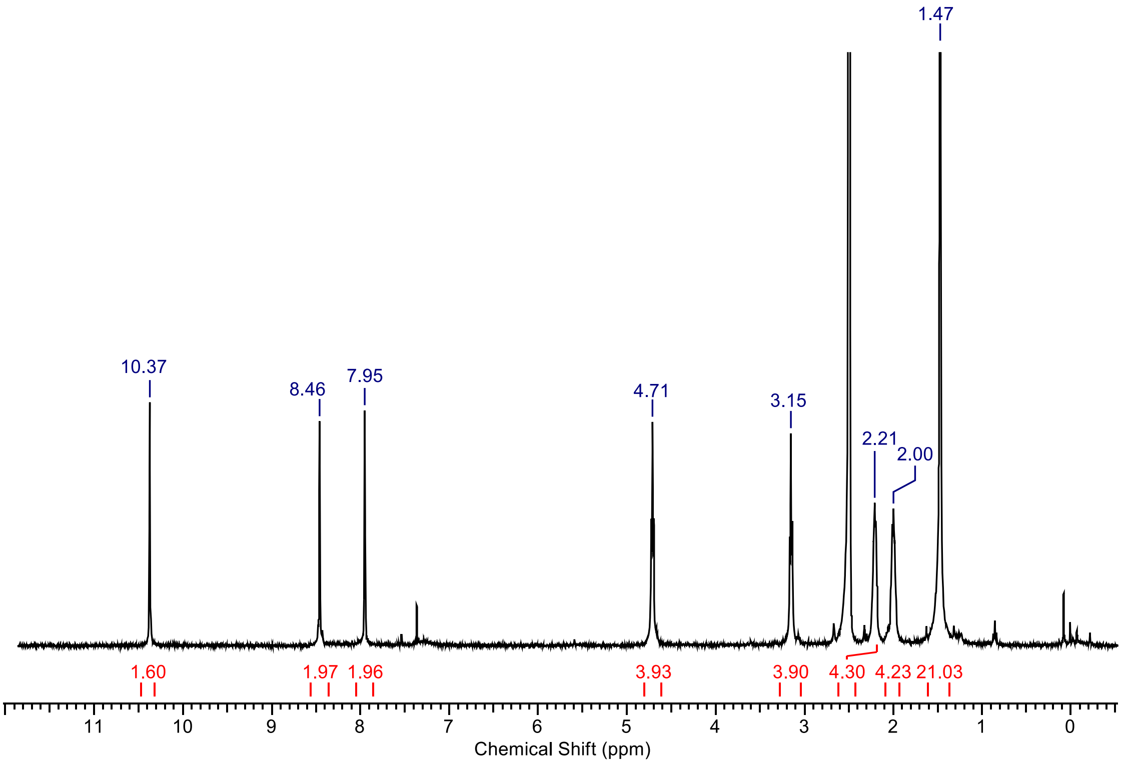

Fig. S12 ^{1}H NMR spectrum of compound **4**, measured in DMSO$_{d6}$.

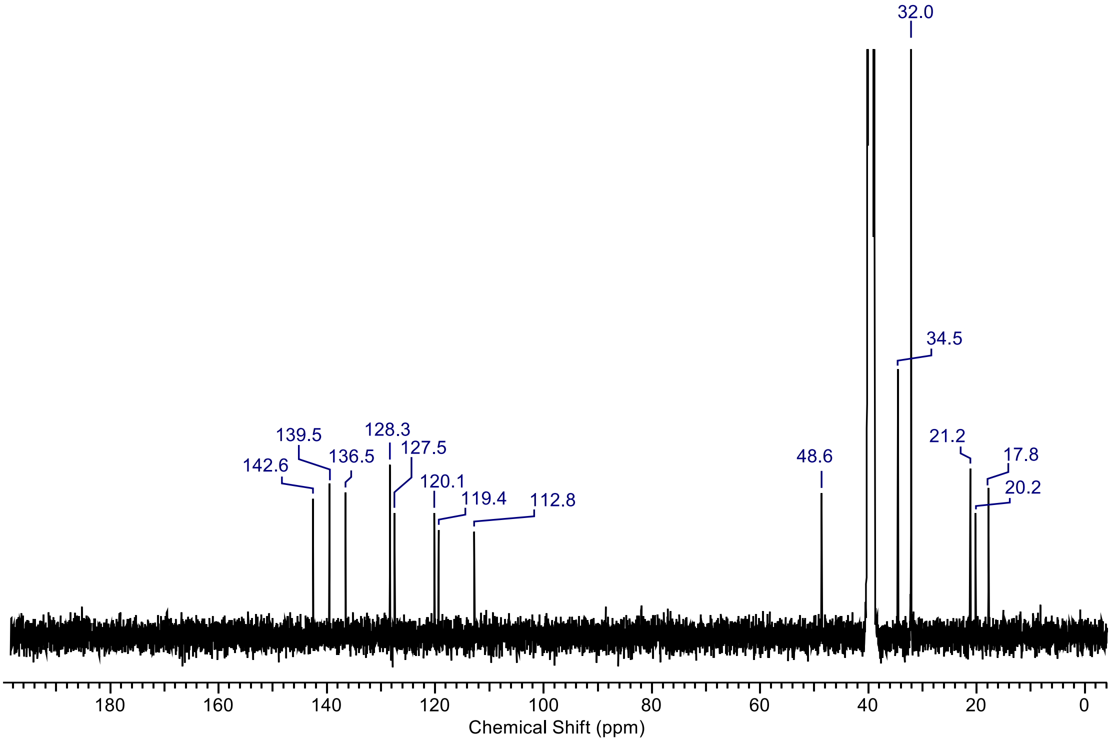

Fig. S13 ^{13}C NMR spectrum of compound **4**, measured in DMSO$_{d6}$.

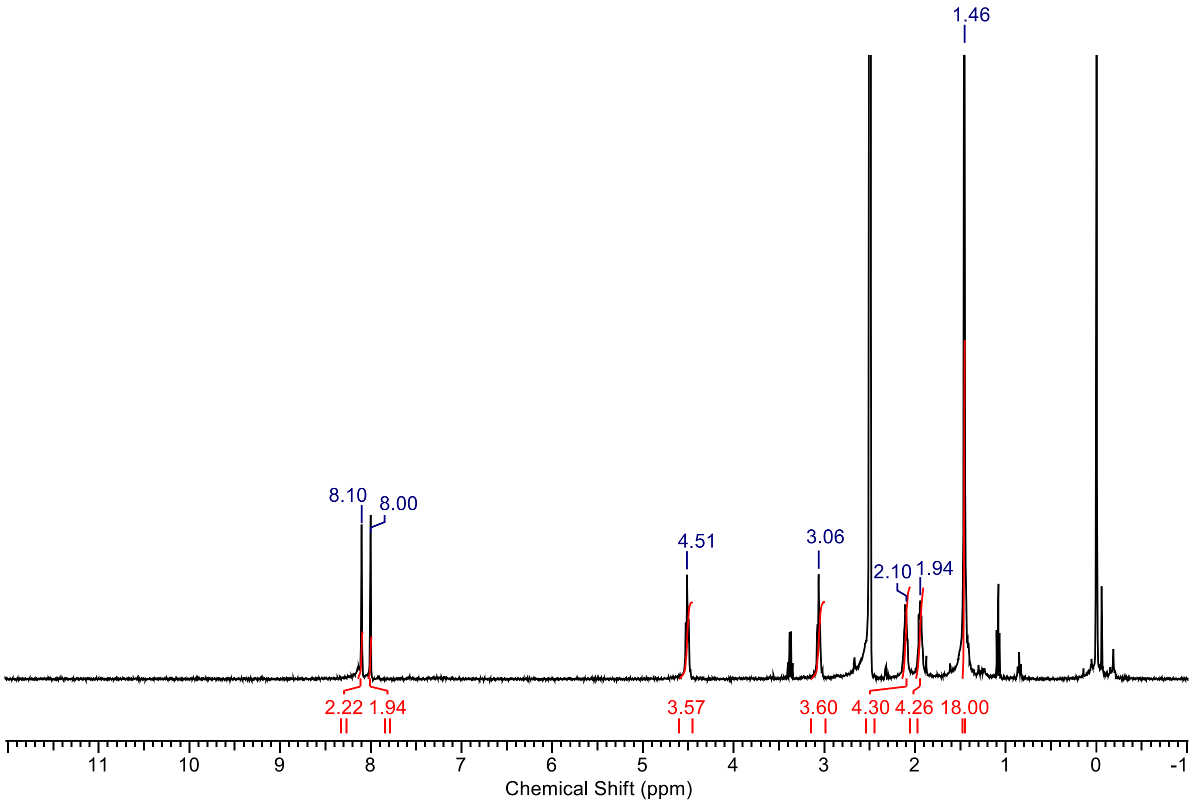

Fig. S14 ^{1}H NMR spectrum of compound ^{Li}**5**, measured in $DMSO_{d6}$.

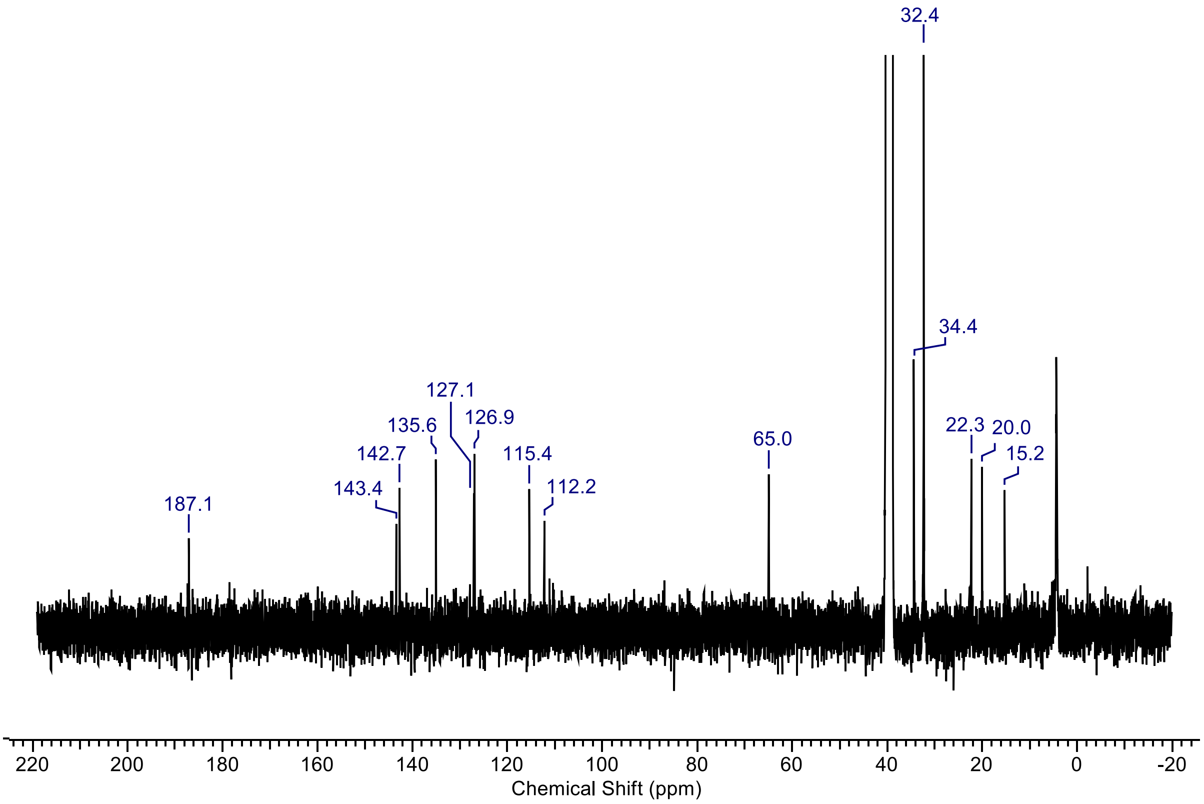

Fig. S15 ^{13}C NMR spectrum of compound ^{Li}**5**, measured in $DMSO_{d6}$.

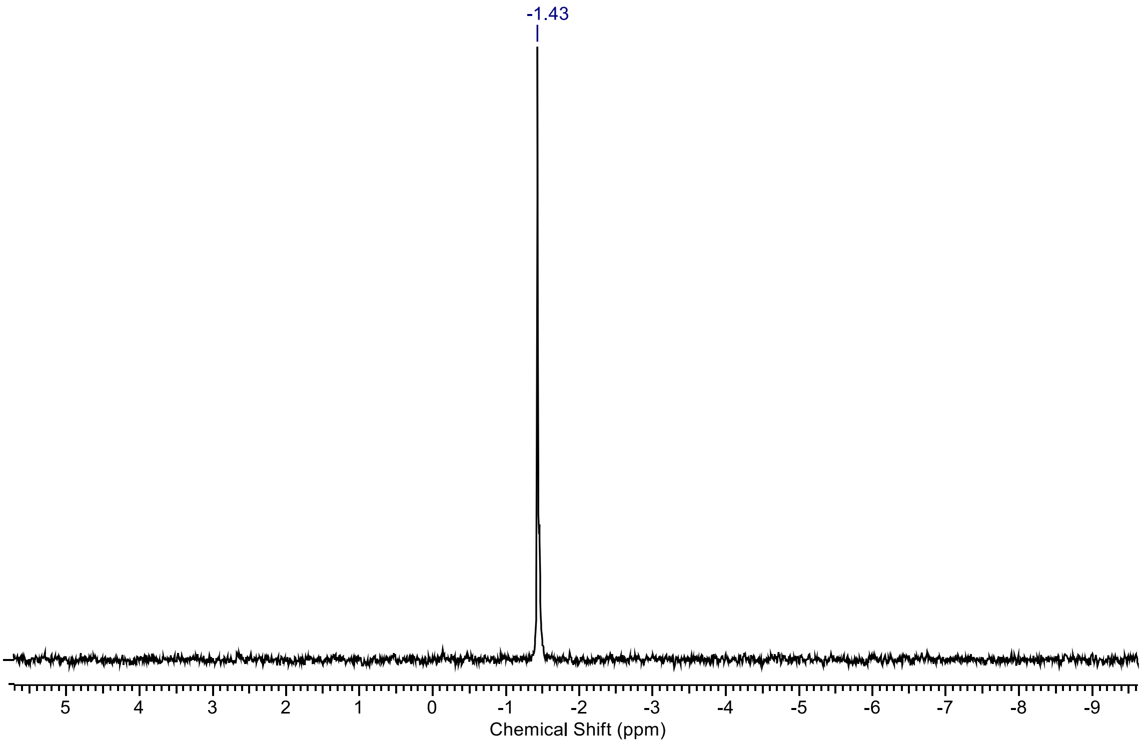

Fig. S16 ^{7}Li NMR spectrum of compound **[Li]5**, measured in DMSO[d6].

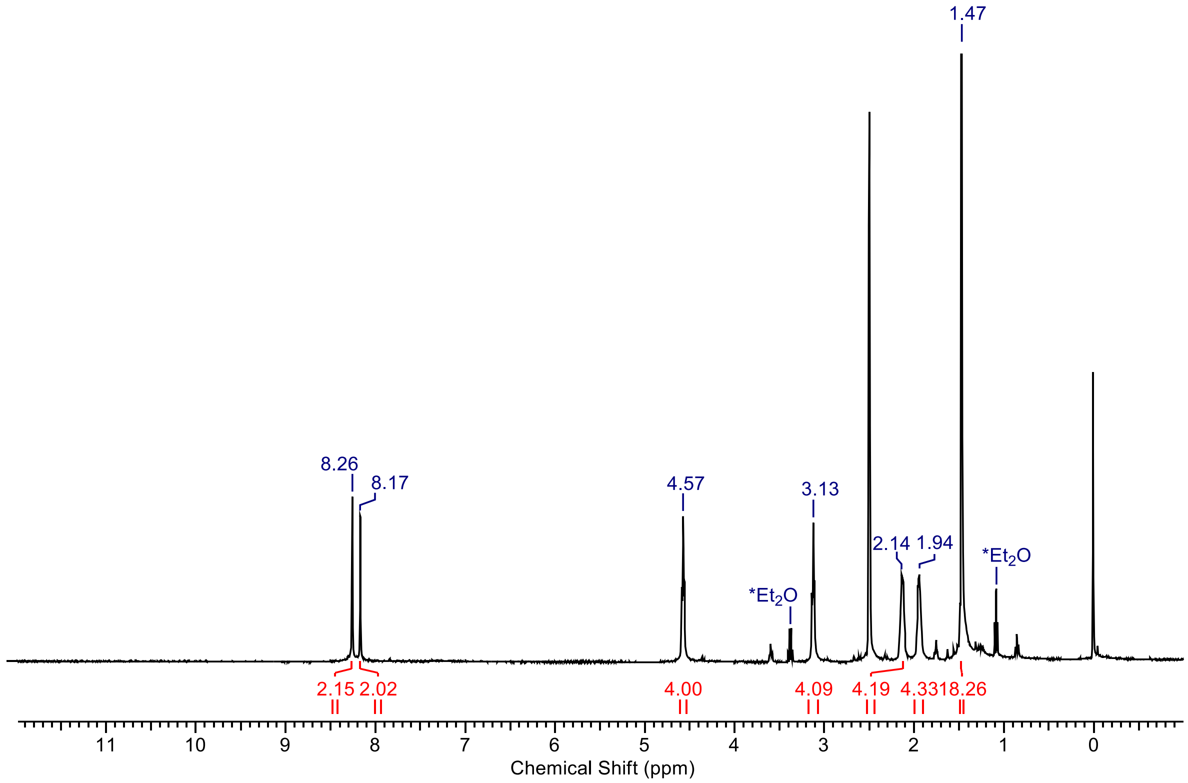

Fig. S17 ^{1}H NMR spectrum of compound **[MgBr]5**, measured in DMSO[d6].

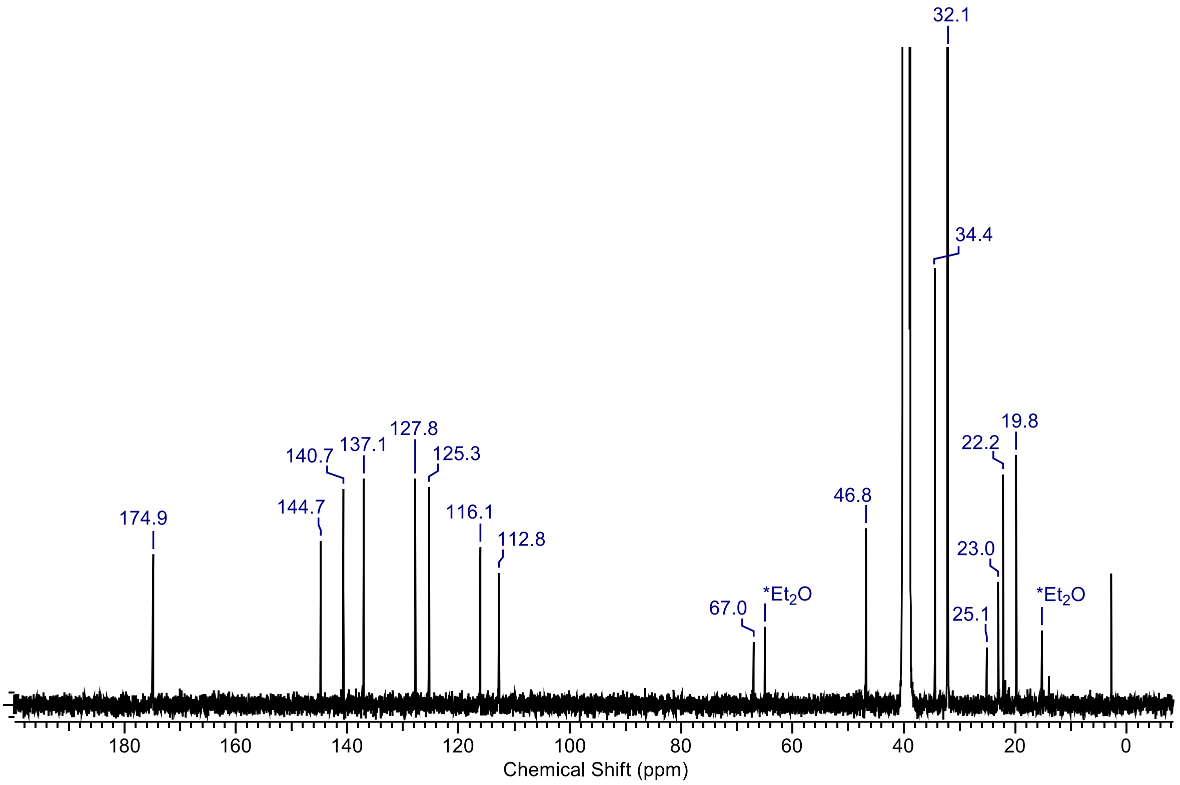

Fig. S18 ^{13}C NMR spectrum of compound $^{\mathbf{MgBr}}\mathbf{5}$, measured in $DMSO_{d6}$.

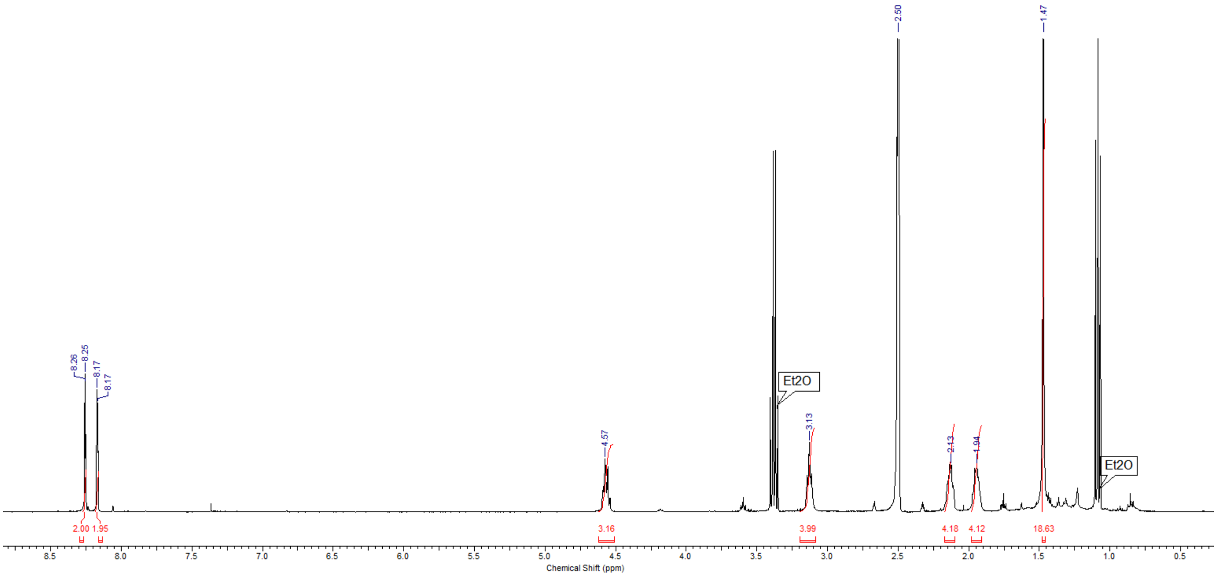

Fig. S19 *In situ* ^{1}H NMR spectrum of compound $^{\mathbf{MgBr}}\mathbf{5}$ obtained *via* the MeMgBr route, measured in $DMSO_{d6}$.

2. Photospectroscopic Investigations

UV/vis absorption spectra between 300 and 1000 nm were recorded on a Lambda2 (PerkinElmer) dual beam absorption spectrometer with a scan rate of 480 nm/min and a resolution of 1 nm. Luminescence spectra were recorded on a Fluoromax-3 spectrometer (Horiba Yobin Yvon) with 1 nm spectral bandwidth and 0.1 s integration time, if not stated otherwise. Quantum yields (Φ^{em}) were obtained using Coumarin-102 (Φ^{em} = 76% in EtOH[5]) and Coumarin-151 (Φ^{em} = 49% in EtOH[6]). TCSPC experiments were measured with a FS5-TCSPC spectrofluorometer from Edinburgh Instruments equipped with a photomultiplier R928P emission detector. Samples were excited by a VISUV versatile picosecond laser module from Picoquant. The repetition rate was 8 MHz. All samples for photo spectroscopic investigations of the air- and moisture sensitive compounds **^{Li}5**, **^{MgBr}5**, and **4** were prepared in an argon-filled glovebox and hermetically sealed prior to the measurement. All glass containers (including vials and quartz glass cuvettes) were silylated prior to the transfer into the glovebox using standard procedures with dimethyldichlorosilane (DMDCS). Thereby, all glassware was soaked in a 10% DMDCS solution in toluene for 30 minutes and then rinsed twice with toluene. Subsequently, it was soaked in methanol for 15 minutes, rinsed twice with methanol and then finally dried in an oven at 160 °C for at least two hours before transferring them into the glovebox. Benzene for photo spectroscopic investigation was distilled over a NaK alloy and stored over a potassium mirror.

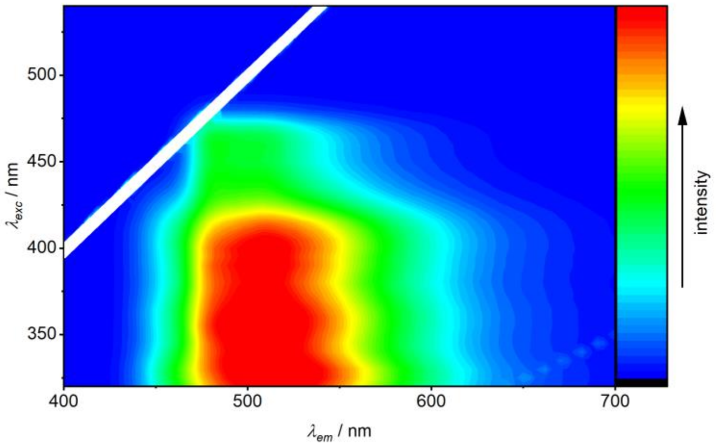

Fig. S20 3D steady-state fluorescence spectroscopy of **^{Li}5** in benzene. The fluorescence intensity is depicted as color code. The white bar masks signals stemming from the scattered excitation light.

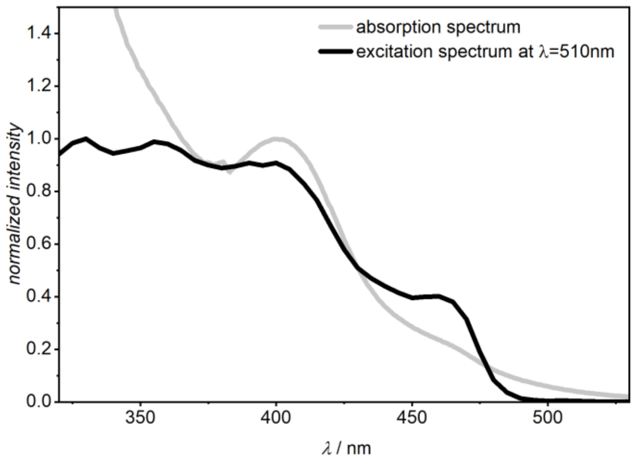

Fig. S21 Comparison between the absorption spectrum of ^{Li}**5** (grey) and the excitation spectrum that leads to photoluminescence at 510 nm (black). The last absorption that leads to photoluminescence is located at the 460 nm shoulder and was used to determine the Stokes-shift of ^{Li}**5**. The spectra were measured in benzene.

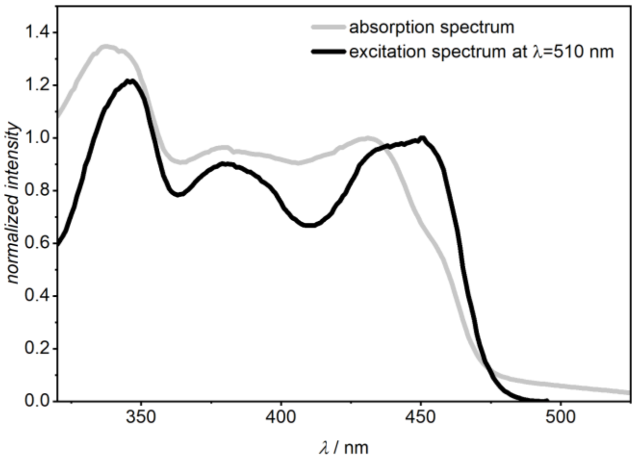

Fig. S22 Comparison between the absorption spectrum of ^{MgBr}**5** (grey) and the excitation spectrum that leads to photoluminescence at 510 nm (black). The spectra were measured in benzene.

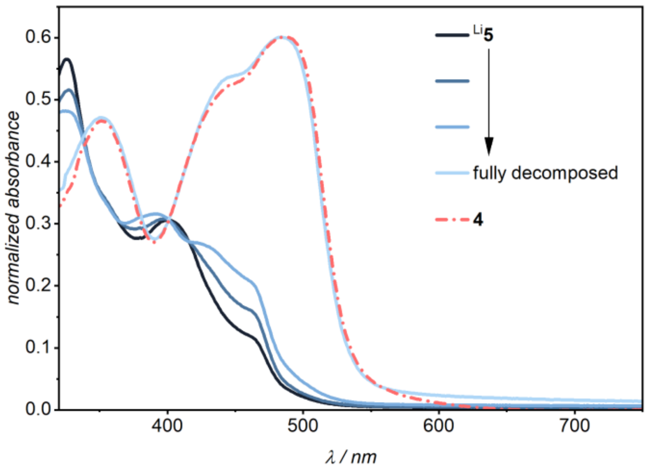

Fig. S23 Normalized absorption spectra of ^{Li}**5** at different stages of decomposition compared to an absorption spectrum of **4** (light red). As dissolved (black), slightly decomposed (dark blue and blue), and fully decomposed (light blue) after exposure to air.

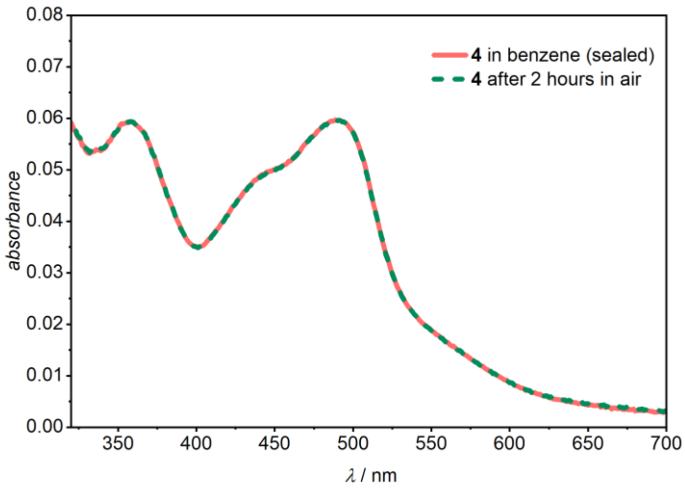

Fig. S24 Comparison of absorption spectra of **4** in benzene in a sealed container (light red) and after 2 hours of exposure to air (green, dashed). Clearly, no spectral changes are discernible.

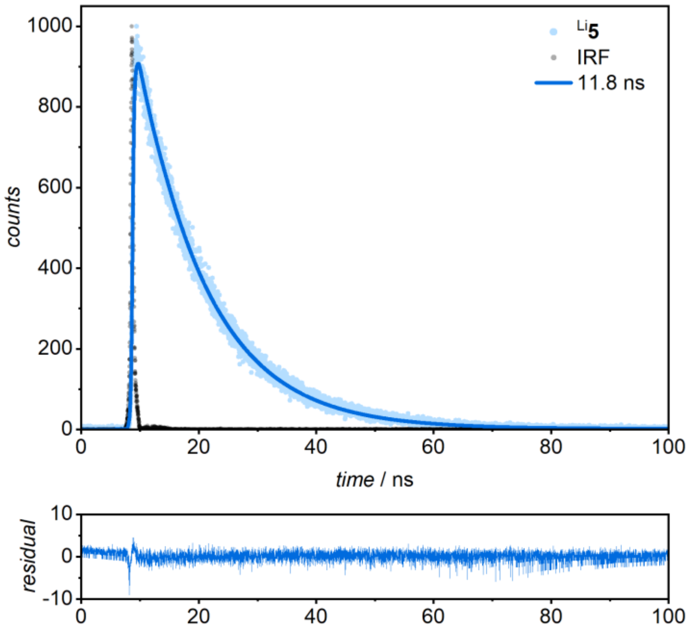

Fig. S25 Top: Luminescence decay (bright blue dots) and fit (blue line) of ^{Li}**5** in benzene (λ_{em} = 500 nm) obtained by time-correlated single-photon counting upon excitation at 355 nm, including the instrument response function (IRF, black dots). A mono-exponential fit was sufficient to fit the dataset (lifetime given in legend). Bottom: Corresponding residual to the TCSPC fit.

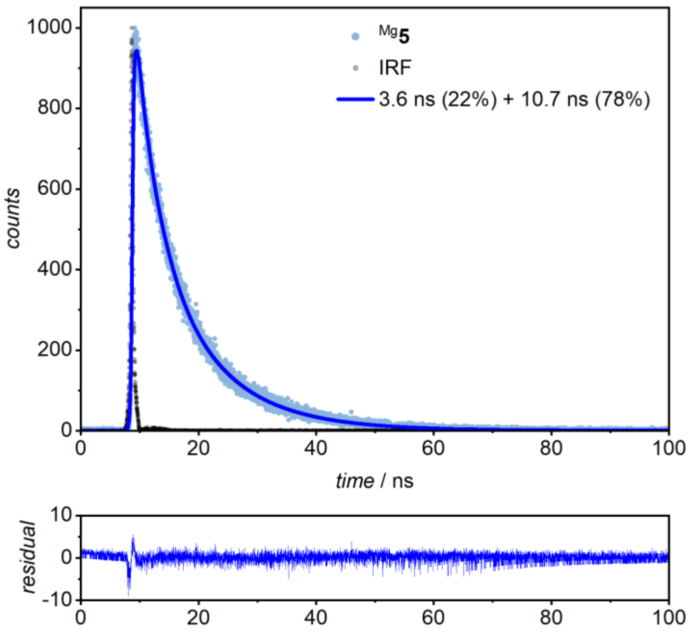

Fig. S26 Top: Luminescence decay (blue dots) and fit (blue line) of **^{MgBr}5** in benzene (λ_{em} = 500 nm) obtained by time-correlated single-photon counting upon excitation at 355 nm, including the instrument response function (IRF, black dots). A bi-exponential fit was sufficient to fit the dataset (lifetimes and contribution given in legend). The second luminescent species is due to slight decomposition of the highly reactive compound during the time of the experiment and corresponds to the decomposition product. Bottom: Corresponding residual to the TCSPC fit.

3. Diffusion NMR (DOSY)

The diffusion NMR experiments (Bruker pulse sequence "ledbpgp2s") were performed on a Bruker Avance III 300 instrument at a probe temperature of 22 °C. Deuterated benzene was used as solvent and an array of 16 runs (32 scans each; 1 s relaxation delay; 50 ms diffusion time) with a gradient pulse of 1.8 ms and the magnetic field gradient ranging from 10 mT m^{-1} – 508 mT m^{-1} was recorded. Each experiment was reproduced once and the values reported in Table S1 are averaged over these two runs.

<u>Sample preparation</u>:

- Complex **^{Li}5** was suspended in benzene and filtered into a J-Young NMR tube to obtain a saturated solution. Note, **^{Li}5** is only very moderately soluble in weakly-coordinating benzene; thus, only the singlet relating to the two *tert*-butyl substituents gives reliable integration.
- Complex **^{MgBr}5** was also suspended in benzene and filtered in a J-Young NMR tube. As for **^{Li}5,** the low solubility allowed for reliable integration of the *tert*-butyl signal only.
- As a reference for the monomer, we used the mono-deprotonated ligand **4**, which required the addition of a stoichiometric amount of $NaBAr^F$ to achieve sufficient solubility in benzene.

Further notes:

- Measuring ^{MgBr}**5** and ^{Li}**5** in one sample for direct comparison is not feasible due to anion scrambling.
- Measuring ^{MgBr}**5** together with mono-deprotonated ligand **4** in the presence of NaBAr^F also leads to shifts for ^{MgBr}**5,** which indicates exchange reactions; however both molecules showed the same drift properties which nevertheless suggests that both molecules are monomers.

Table S1. Diffusion coefficients *D* in [10^{-9} m^2 s^{-1}].

	D ^{Li}**5 (dimer)**	***D*** ^{MgBr}**5 (monomer)**	***D* 4 (monomer)**
**1. Measurement**	1.87 ± 0.30	2.18 ± 0.26	2.11 ± 0.35
**2. Measurement**	1.59 ± 0.40	2.31 ± 0.35	2.17 ± 0.3
**Mean:**	**1.73 ± 0.35**	**2.25 ± 0.31**	**2.14 ± 0.33**

The diffusion coefficient *D* is inversely related to the hydrodynamic radius R_0 according to the Stokes-Einstein equation (Eq. S1):

$$D = \frac{k_b\, T}{6\pi\, \eta\, R_0} \qquad \text{(Eq. S1)}$$

The volume of a sphere with the corresponding hydrodynamic radius is calculated according to Eq. S2.

$$V = \frac{4}{3}\, {R_0}^3\, \pi \qquad \text{(Eq. S2)}$$

This translates into the following volumes

Table S2. Calculated molecular volumes *V* (in 10^{-38} m^3).

	V ^{Li}**5 (dimer)**	***V*** ^{MgBr}**5 (monomer)**	***V* 4 (monomer)**
**Mean:**	**2.98**	**1.36**	**1.57**

The volumes of ^{MgBr}**5** and **4** are similar and the ratio between the volumes suggests that both are monomers (**4** seems to be a bit larger which is agreement with the presence of the bulky anion $^-$BAr^F). The relation of the volumes of ^{MgBr}**5** or and ^{Li}**5** confirms that ^{MgBr}**5** is a monomer and ^{Li}**5** a dimer in solution.

4. X-ray Single Crystal Structure Determinations

Intensity data of single crystals of the investigated compounds were collected using MoK_α radiation (λ = 0.71073 Å) either on a Bruker D8 Quest diffractometer (**3** and **4**) or Bruker AXS D8 Venture, Photon II ($^{\mathbf{Li}}$**5**). All hydrogen atoms were placed in positions of optimized geometry. The isotropic displacement parameters of all hydrogen atoms were tied to those of their corresponding carrier atoms by a factor of 1.2 or 1.5. All non-hydrogen atoms were refined anisotropically. Data were corrected for Lorentz and polarization effects; semiempirical absorption corrections were performed on the basis of multiple scans using *SADABS*.[7] The structures were solved by direct methods using SHELXT.[8] The structures were refined by full-matrix least-squares procedures on F^2 using *SHELXL* (compounds **3**, **4** and $^{\mathbf{Li}}$**5**) in the graphical user interface Shelxle (compound **3** $^{\mathbf{Li}}$**5**).[9] OLEX2 was used to prepare material for publication.[10]

Table S3: Crystallographic details of compounds **3**, **4** and complex $^{\mathbf{Li}}$**5**.

	**Compound 3**	**Compound 4**	**Complex $^{\mathbf{Li}}$5**
Chemical Formula	$C_{32}H_{41}N_7I_2$ 4(CHCl$_3$)	$C_{32}H_{40}N_7I_1$ 3(C_6H_6)	$C_{80}H_{116}I_4Li_6N_{14}O_4$
M_r	1254.99	883.93	1887.10
Crystal System	Orthorhombic	Triclinic	Monoclinic
Space Group	*P*bcm	P-1	P2$_1$/n
a (Å)	12.486(2)	10.5612(6)	14.6296(4)
b (Å)	27.199(3)	15.8193(9)	18.3265(4)
c (Å)	13.881(2)	15.8498(8)	16.3230(4)
α(°)	90	113.980(2)	90
β(°)	90	96.942(2)	93.4710(10)
γ(°)	90	105.305(2)	90
V (Å^3)	4714(1)	2254.1(2)	4368.32(19)
Z	4	2	2
Density (g cm^{-3})	1.768	1.302	1.435
F(000)	2480	920	1912
Radiation Type	MoK_α	MoK_α	MoK_α
μ (mm^{-1})	2.051	0.752	1.480
Crystal Size	0.12x0.09x0.05	0.05x0.04x0.03	0.17x0.14 x 0.10
Meas. Refl.	106086	54094	76358
Indep. Refl.	4390	8291	10413
Obsvd. [$I > 2\sigma(I)$]	3648	5776	9399
R_{int}	0.0597	0.1527	0.0328
Data / restraints / parameters	4390/1042/455	8291/529/14	10413/114/583
R1 [$F^2 > 2\sigma(F^2)$]	0.0462	0.0536	0.0202
wR2(F^2) [all data]	0.1350	0.0978	0.0478
S	1.052	1.113	1.038
$\Delta\rho_{max}$	1.608	0.511	0.491
$\Delta\rho_{min}$	-1.117	-0.472	-0.482
CCDC	1953709	2047516	2060428

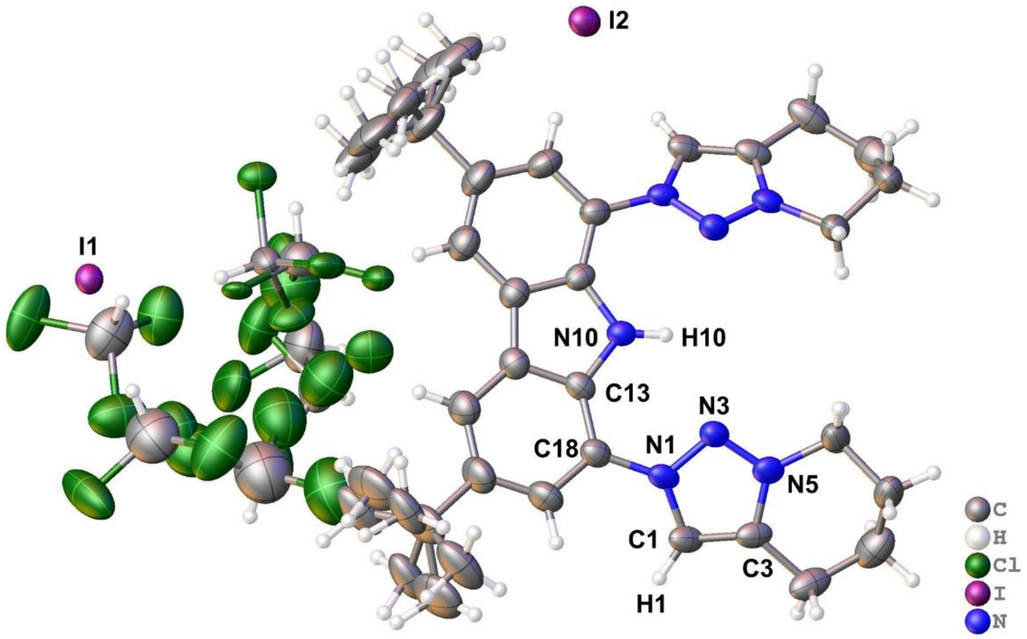

Fig. S27 Solid-state structure of **3,** including the solvent molecules and the disorders with the applied numbering scheme on relevant atoms. Thermal ellipsoids are shown at the 50% probability level.

The tert-butyl group is disordered over two positions. The occupancy of the minor position refined to 0.458(8). The disordered chloroform molecules were refined on seven positions. Their occupancies were refined and then fixed to 0.12, 0.37, 0.43, 0.17, 0.45, 0.28 and 0.18, respectively. All disordered groups were refined with distance restraints and restraints or constraints for the anisotropic displacement parameters.

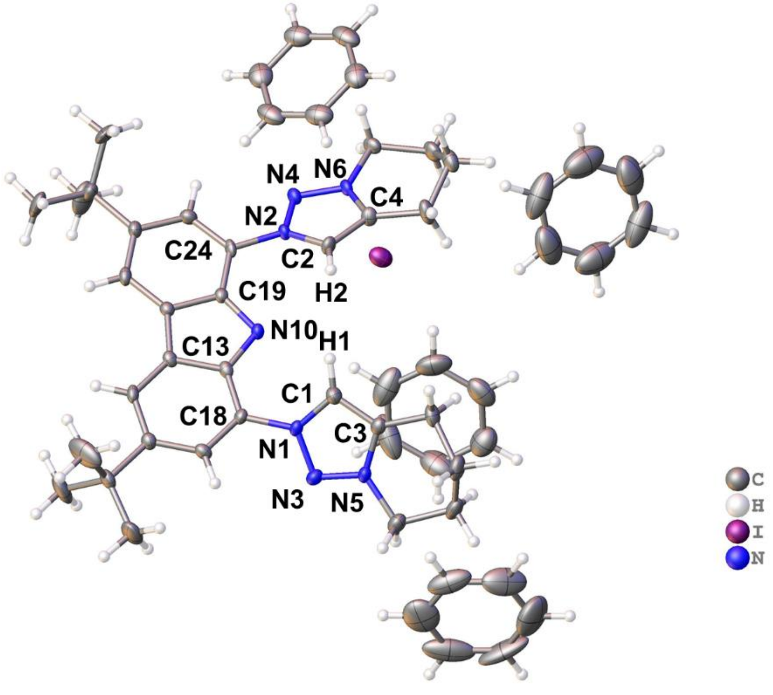

Fig. S28 Solid-state structure of **4,** including the solvent molecules with the applied numbering scheme on relevant atoms. Thermal ellipsoids are shown at the 50% probability level.

The lattice benzene molecules were found to be strongly disordered and needed to be modeled using DFIX restraints fixing the bond distances of the carbon atoms.

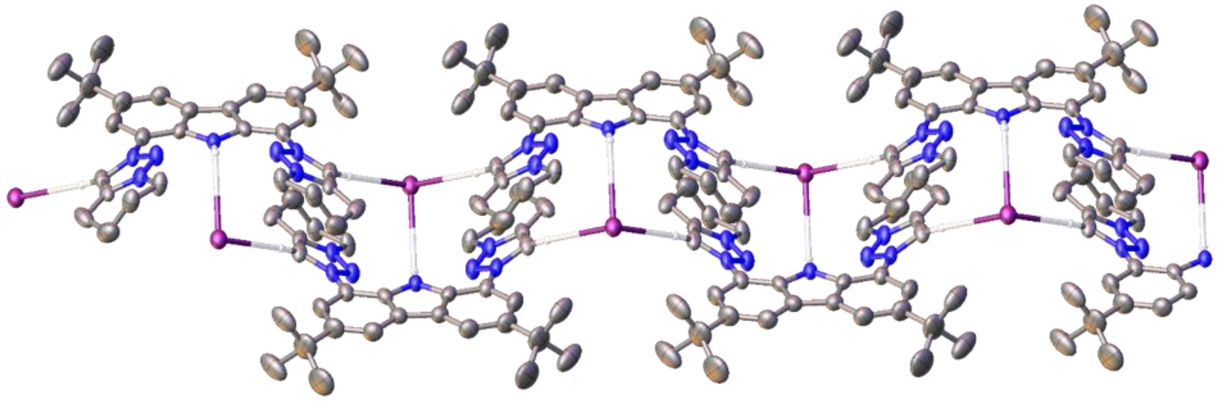

Fig. S29 In the solid-state structure of **3** there are hydrogen bond-type interactions between H10–N3 (2.351(19) Å), H10–I2 (3.08 (7) Å) and H1–I2 (2.83(1) Å).

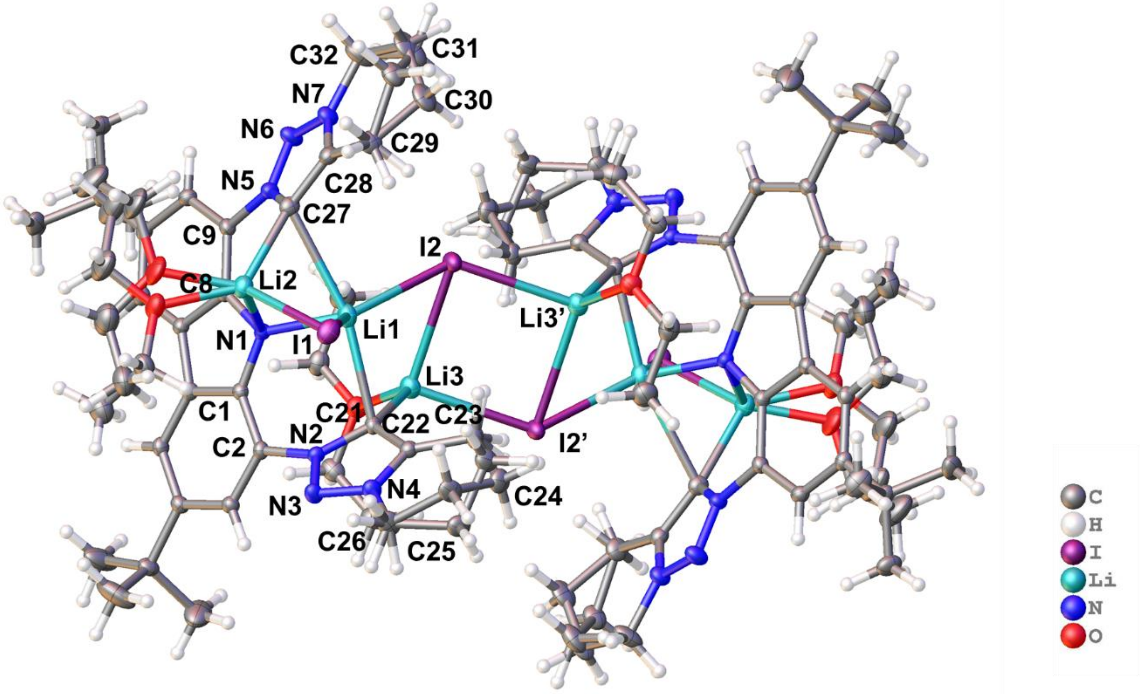

Fig. S30 Solid-state structure of ^{Li}**5** including structural disorders, with the applied numbering scheme on relevant atoms. Thermal ellipsoids are shown at the 50% probability level.

The coordinated diethyl ether on Li2 is disordered over two positions. Its occupancy factors refined to 88.8 % (A) and 11.2 % (B). Additionally, C24, C25 and C30, C31 of the six-membered rings were split over two positions. The occupancy factors of their major position refined to 69.7 % and 90.1 %, respectively. For the refinement of the disorder distance restraints and restraints for the anisotropic displacement parameters were applied.

5. Computational Details – General

The calculations were performed with ORCA 4.2.1.[11, 12] All calculated structures were verified as true minima by the absence of negative eigenvalues in the harmonic vibrational frequency analysis. Potential conformers were assessed for all structures, but only the most stable ones are given below. Tighter than default convergence criteria (tightopt), grid values (grid5, finalgrid6) were chosen for both the optimization of the structural parameters and the scf (tightscf). The geometry optimizations were performed at the B3LYP-D3(BJ)/def2-SVP level of theory.[13-18] The RIJCOSX approximation (gridx5) and the related auxiliary basis set def2/J were used to speed up the calculations.[19-21] The absorption spectra were initially modelled by time-dependent DFT (TD-DFT) as single point calculations at the triple-□ level of theory (BP86[22, 23], B3LYP[13-16], cam-B3LYP[24], M06-2X[25], TPSS[26], TPSSh[27], ωB97XD[28], Figs. S30-S32) using the structural parameters obtained from the B3LYP-D3(BJ)/def2-SVP calculations. The geometry optimizations of the excited S_1 states were performed at the TD-DFT B3LYP-D3(BJ)/def2-SVP level of theory.[13-18, 29] The vibronically resolved spectrum was obtained with the ESD[30, 31] module as implemented in ORCA 4.2.1 at the B3LYP-D3(BJ)/def2-TZVPP//B3LYP-D3(BJ)/def2-SVP level of theory with the adiabatic hessian model (hessflag AH).[13-18, 29] The DLPNO-STEOM-CCSD calculations were performed using the def2-TZVPP basis set and the def2-TZVPP/C auxiliary basis set using the structural parameters obtained from the B3LYP-D3(BJ)/def2-SVP calculations.[21, 29, 32] Ten (compounds **5**) to twenty (compounds **3**, **4**) roots were included in the calculations. For the DLPNO-STEOM-CCSD calculation, correction for solvation effects was performed using the SMD model (SMD true and smdsolvent "benzene").[33] Molecular orbitals and optimized geometries were visualized with Chemcraft, IBOView and Avogadro.[34, 35].

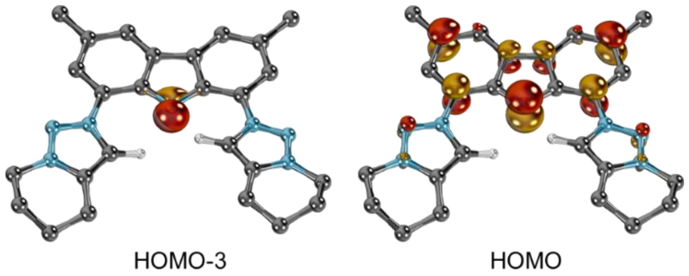

Fig. S31 Relevant molecular orbitals as obtained at DFT B3LYP-D3(BJ)/def2-TZVPP level of theory show that compound **4** contains a "naked" amide.

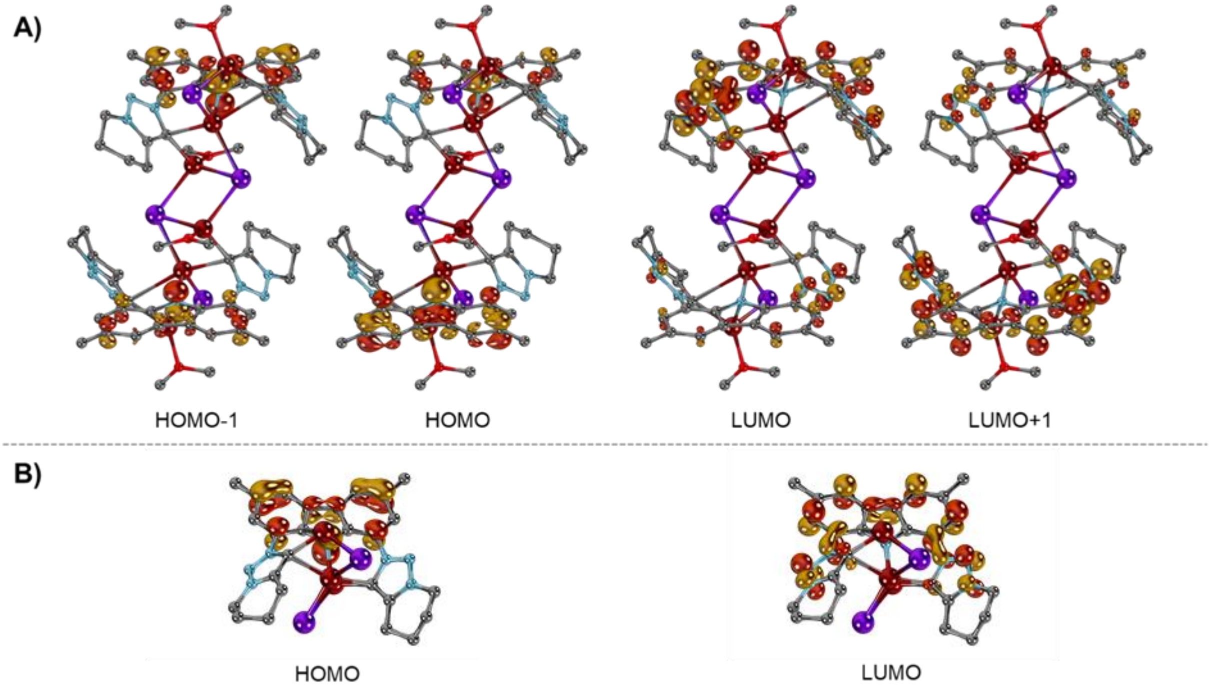

Fig. S32 **A**) The bright absorption to the S_1 state of **^{Li}5** is approximated as the HOMO → LUMO transition. Note that the HOMO and HOMO-1 are degenerate due to symmetry (top left), the same holds true for LUMO and LUMO+1(top right). The HOMO (HOMO-1) is located at the carbazole, whereas the LUMO (LUMO+1) is mainly localized on one of the two CNC ligands in the triazolium groups. **B**) The electronic structure of **^{Li}5^{truncated}** is equivalent to **^{Li}5**. Hydrogen atoms are omitted for clarity. Results obtained at the TD-DFT B3LYP-D3(BJ)(SMD)/def2-TZVPP//B3LYP-D3(BJ)/def2-SVP level of theory.

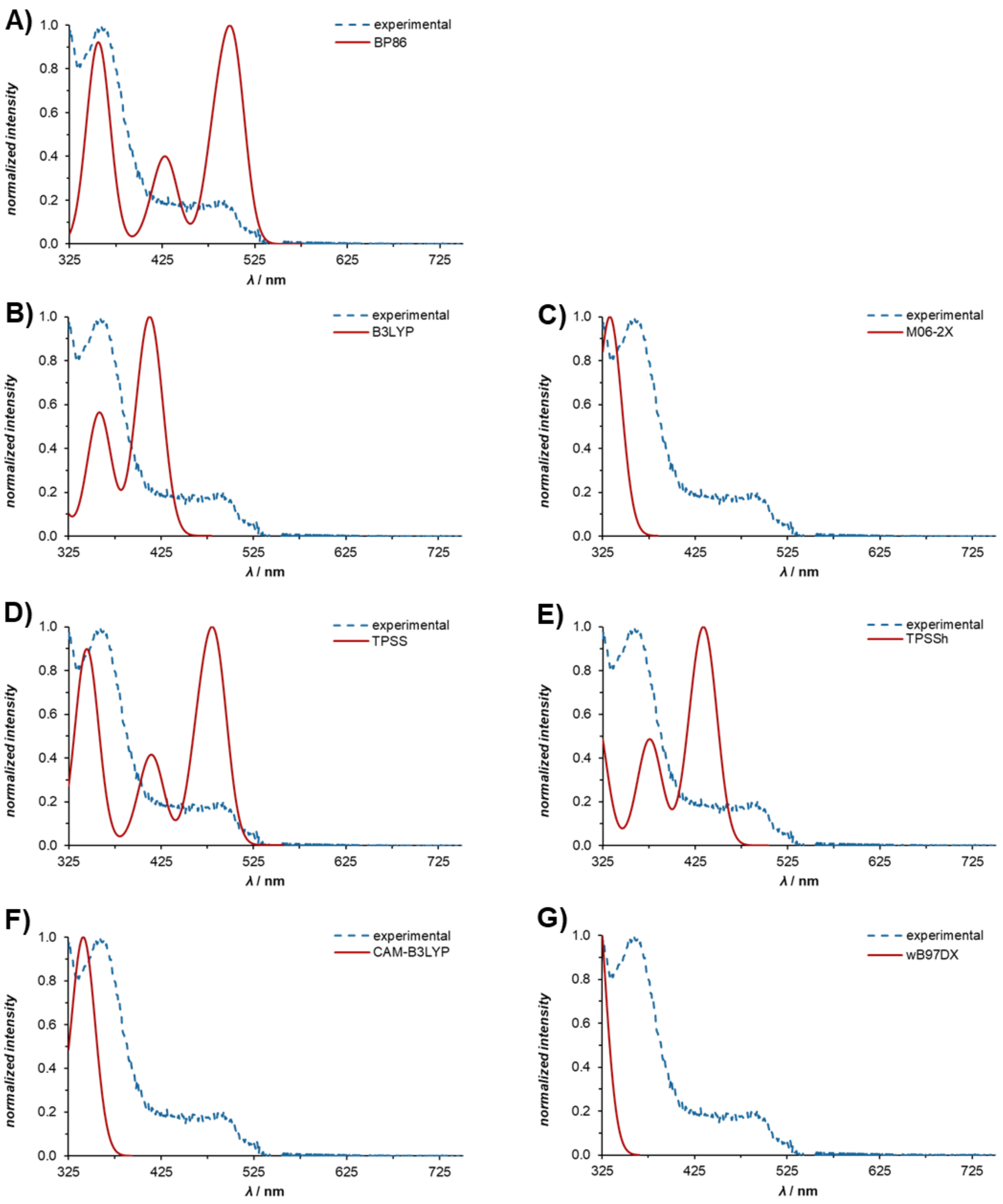

Fig. S33 Predicted absorption spectrum of compound **3** obtained with different DFT functionals **A**) BP86, **B**) B3LYP, **C**) M06-2X, **D**) TPSS, **E**) TPSSh, **F**) cam-B3LYP, **G**) ωB97XD. Results obtained at the TD-DFT (SMD)/def2-TZVPP//B3LYP-D3(BJ)/def2-SVP level of theory.

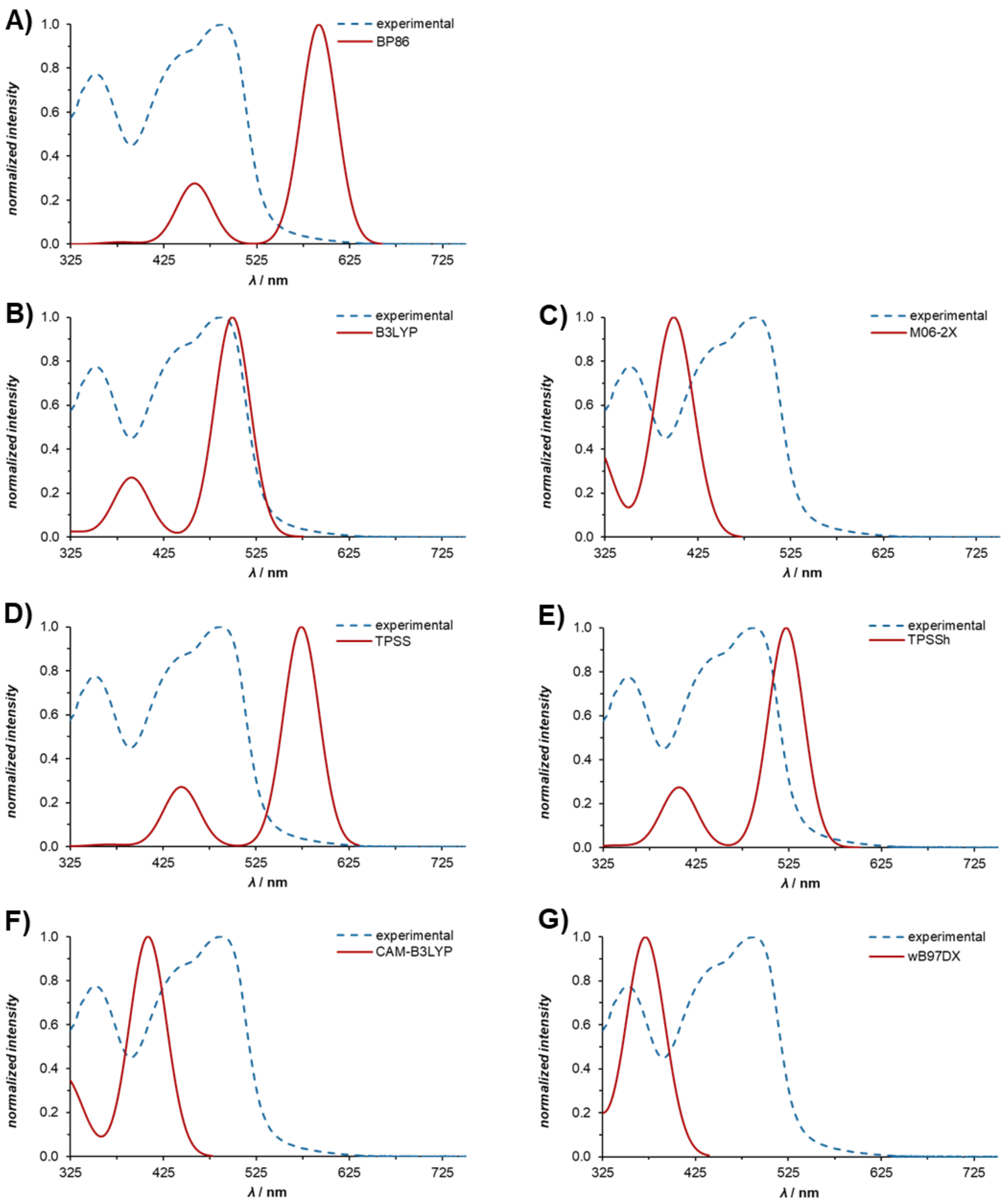

Fig. S34 Predicted absorption spectrum of compound **4** obtained with different DFT functionals **A**) BP86, **B**) B3LYP, **C**) M06-2X, **D**) TPSS, **E**) TPSSh, **F**) cam-B3LYP, **G**) ωB97XD. Results obtained at the TD-DFT (SMD)/def2-TZVPP//B3LYP-D3(BJ)/def2-SVP level of theory.

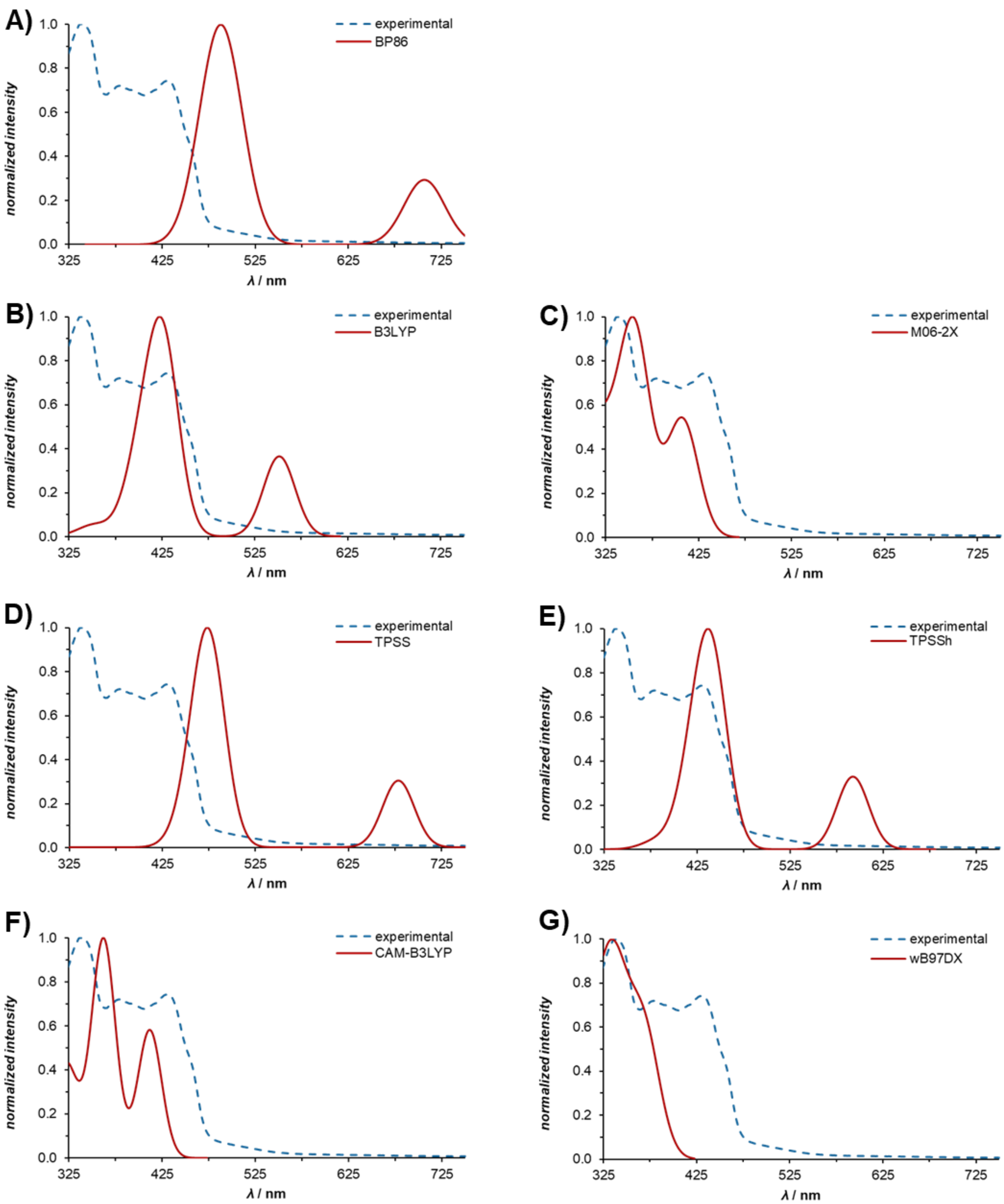

Fig. S35 Predicted absorption spectrum of compound ^{MgBr}**5** obtained with different DFT functionals **A**) BP86, **B**) B3LYP, **C**) M06-2X, **D**) TPSS, **E**) TPSSh, **F**) cam-B3LYP, **G**) ωB97XD. Results obtained at the TD-DFT (SMD)/def2-TZVPP//B3LYP-D3(BJ)/def2-SVP level of theory.

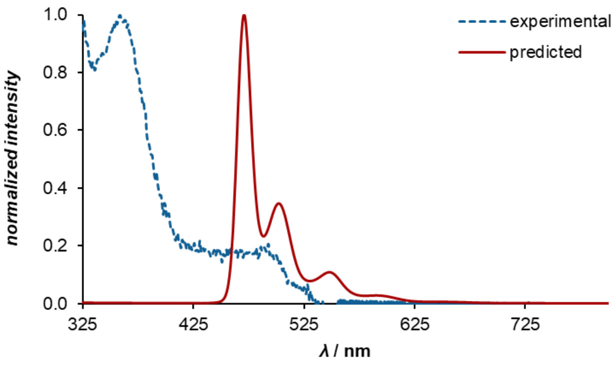

Fig. S36 Molecular vibrations are responsible for the plateau in the absorption spec¬trum of **3**. The vibronically resolved spectrum has been obtained at the B3LYP Def2-TZVPP level of theory.

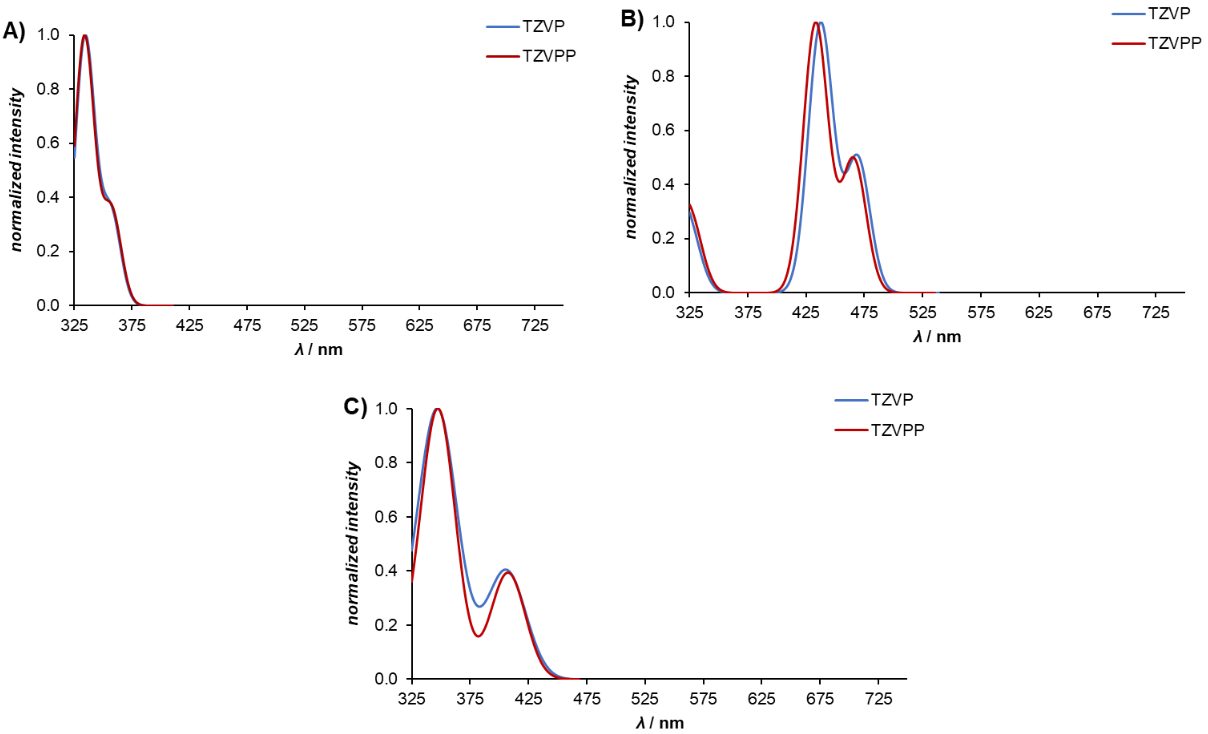

Fig. S37 The basis set def2-TZVP (blue) and def2-TZVPP (red) give comparable results for compounds **3** (**A**), **4** (**B**) and ^{MgBr}**5** (**C**). Results obtained at the DLPNO-STEOM-CCSD(SMD=C_6H_6)/def2-TZVP (or def2-TZVP) //B3LYP-D3(BJ)/def2-SVP level of theory.

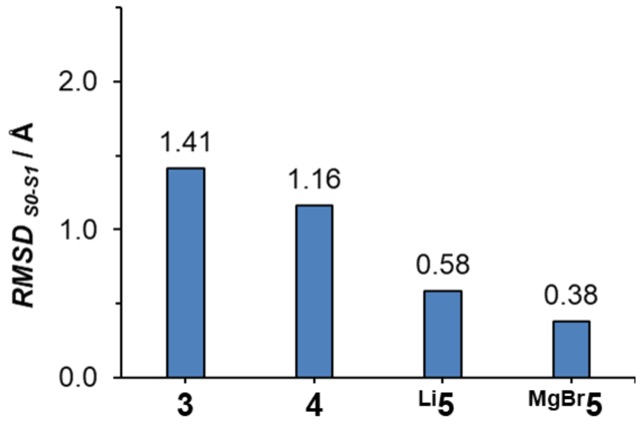

Fig. S38 Structural distortion arising from the excitation process is quantified by the root-mean-square deviation for the change of all the atomic positions. Results obtained at the TD-DFT B3LYP-D3(BJ)(SMD)/def2-TZVPP//B3LYP-D3(BJ)/def2-SVP level of theory.

6. XYZ Coordinates

Compound 3 Ground State (S_0)

C	-6.69740	1.79207	5.09392
C	-5.77400	2.69552	5.63615
C	-4.44385	2.76991	5.19111
C	-4.01420	1.89356	4.18067
C	-4.94962	0.97411	3.62214
C	-6.26839	0.92776	4.07068
H	-6.11841	3.35220	6.43389
H	-6.97356	0.21956	3.62887
C	-2.89126	0.71336	2.61921
C	-1.94724	0.13245	1.75600
C	-2.35995	-0.87571	0.86922
C	-3.68224	-1.34103	0.83349
C	-4.61230	-0.78983	1.73084
C	-4.22806	0.22247	2.60834
H	-1.64396	-1.30892	0.17007
H	-5.64139	-1.15718	1.73135
C	-8.12114	1.75983	5.58625
H	-8.81692	2.06753	4.78865
H	-8.41249	0.74504	5.90034
H	-8.27431	2.43503	6.43948
C	-4.11216	-2.39097	-0.15762
H	-4.72487	-3.16522	0.32913
H	-4.72678	-1.93988	-0.95453
H	-3.25486	-2.88467	-0.63715
C	-3.88435	4.68599	6.70613
C	-2.71113	5.36054	6.97353
C	0.49185	-0.14925	1.24335
C	1.60790	0.60449	1.54864
N	-3.56805	3.73388	5.77376
N	-2.28996	3.78196	5.44353
N	-1.78581	4.76123	6.17113
N	-0.57338	0.51903	1.78565
N	-0.19394	1.62281	2.40167
N	1.11648	1.66189	2.25767
C	3.07089	0.43272	1.28816
H	3.44604	-0.35523	1.96451
H	3.22005	0.05576	0.26501
C	3.83049	1.74436	1.53164
H	3.65598	2.44165	0.69461
H	4.90968	1.53852	1.55201
C	3.38209	2.39284	2.84674
H	3.97128	3.29602	3.06342
H	3.54683	1.69860	3.68836
C	1.90786	2.79140	2.78069
H	1.74930	3.64101	2.09761
H	1.49070	3.05536	3.76250
C	-2.35794	6.45376	7.92987
H	-3.13437	7.23324	7.89428
H	-2.39456	6.02550	8.94756
C	-0.96451	7.03206	7.63751
H	-0.62492	7.61535	8.50460
H	-1.02151	7.73463	6.78902
C	0.04138	5.92172	7.31694
H	0.12709	5.22228	8.16582
H	1.04248	6.34615	7.14922
C	-0.36941	5.15307	6.06045
H	0.20963	4.23044	5.91581
H	-0.26755	5.77584	5.15728
N	-2.78980	1.71764	3.56431
H	-4.87923	4.82348	7.11336
H	0.40018	-1.09431	0.71959
H	-1.95965	2.26538	3.74667

Compound 3 Excited State (S_1)

C	-7.29760	3.21954	3.39760
C	-6.36739	4.25696	3.55976
C	-4.99546	3.99512	3.67940
C	-4.54284	2.67320	3.65499
C	-5.47623	1.60895	3.48998
C	-6.83016	1.87849	3.36205
H	-6.70674	5.29333	3.58412
H	-7.54726	1.06353	3.23822
C	-3.33721	0.77288	3.69249
C	-2.26763	-0.14641	3.80566
C	-2.59300	-1.47494	3.80792
C	-3.95177	-1.92382	3.60608
C	-5.00039	-0.98627	3.48338
C	-4.69637	0.36805	3.52982
H	-1.80682	-2.22318	3.92002
H	-6.02601	-1.33633	3.35424
C	-8.76225	3.50482	3.24058
H	-9.10826	3.19841	2.23848
H	-9.35338	2.92819	3.97042
H	-8.99286	4.57037	3.37035
C	-4.20858	-3.38716	3.50338
H	-5.27422	-3.62288	3.38891
H	-3.66045	-3.81576	2.64394
H	-3.81730	-3.90782	4.39557
C	-2.92262	5.30826	3.11032
C	-2.43578	6.51912	3.56691
C	0.22312	-0.37268	3.83945
C	0.97866	-0.14008	4.94211
N	-4.07602	5.08737	3.81539
N	-4.33510	6.06113	4.66248
N	-3.35375	6.91293	4.50456
N	-0.95021	0.41299	3.95963
N	-0.93429	1.08489	5.20494
N	0.24331	0.71072	5.75083
C	2.32430	-0.63451	5.38240
H	2.21046	-1.66460	5.76213

H	2.99596	-0.69505	4.51359
C	2.90383	0.25444	6.49144
H	3.22891	1.22224	6.07088
H	3.79895	-0.22336	6.91424
C	1.85588	0.48961	7.58299
H	2.27901	1.05851	8.42386
H	1.52295	-0.48006	7.99112
C	0.65425	1.25941	7.03580
H	0.90810	2.32462	6.88963
H	-0.21038	1.21270	7.71505
C	-1.25928	7.37064	3.20567
H	-0.37958	6.72969	3.04411
H	-1.47707	7.84851	2.23407
C	-0.99874	8.44150	4.27655
H	-0.28597	9.17836	3.88183
H	-0.52238	7.98200	5.15945
C	-2.30488	9.12849	4.68847
H	-2.78122	9.60052	3.81237
H	-2.11452	9.93241	5.41440
C	-3.27131	8.13831	5.33344
H	-4.29288	8.52914	5.43015
H	-2.92547	7.82013	6.32949
N	-3.26728	2.13011	3.76723
H	-2.56936	4.63620	2.33234
H	0.43362	-0.96434	2.95418
H	-2.41896	2.59119	4.09622

Compound 4 Ground State (S_0)

N	1.53514	0.75769	9.81420
N	3.53411	0.15550	13.62883
N	3.47360	1.30680	14.26996
N	-0.51965	3.37097	7.03148
N	2.89897	0.36807	12.49093
N	-0.31990	4.60385	7.45672
N	0.19268	2.61373	7.84445
C	0.94619	0.41179	8.62727
C	1.06142	-0.99483	8.33698
C	1.81473	-1.53542	9.44766
C	0.49089	-1.55851	7.20317
H	0.59444	-2.63032	7.01015
C	0.26459	1.20600	7.67513
C	2.04512	-0.40883	10.31749
C	-0.22952	-0.75654	6.29425
C	0.50697	4.66910	8.54009
C	-0.31992	0.61727	6.53939
H	-0.84656	1.25711	5.83116
C	0.84832	3.35643	8.79590
H	1.48013	2.84785	9.52071
C	2.43525	1.65705	12.39597
H	1.87102	1.95164	11.51364
C	2.72440	-0.66715	11.53273
C	2.28324	-2.80506	9.75439
H	2.09409	-3.63977	9.07360
C	3.66052	2.75973	16.23964
H	4.33187	2.96478	17.08627
H	2.64763	2.64147	16.66115
C	2.82134	2.27756	13.56785
C	4.10229	1.44965	15.58879
H	5.19339	1.41712	15.44088
H	3.81227	0.57261	16.18438
C	3.19832	-1.95925	11.82311
H	3.72364	-2.12184	12.76405
C	3.00468	-3.02986	10.94411
C	0.81531	5.97250	9.20918
H	1.85900	5.97237	9.55884
H	0.18410	6.05319	10.11201
C	3.66745	3.92248	15.24121
H	3.41384	4.86251	15.75225
H	4.67794	4.04942	14.81491
C	-0.84124	6.99461	7.60002
H	-1.63068	6.93711	8.36918
H	-1.07910	7.86297	6.96770
C	-0.88130	5.74463	6.72203
H	-1.89887	5.47015	6.41142
H	-0.27416	5.87818	5.81201
C	2.65979	3.66375	14.11249
H	1.63126	3.78271	14.49799
H	2.77848	4.38919	13.29299
C	0.52918	7.15608	8.27190
H	1.31450	7.21990	7.49904
H	0.57264	8.09387	8.84484
C	-0.88082	-1.37868	5.08345
H	-0.14503	-1.91957	4.46505
H	-1.64922	-2.11143	5.38126
H	-1.36773	-0.62420	4.44822
C	3.54411	-4.40513	11.24994
H	3.99242	-4.45510	12.25313
H	2.74982	-5.16713	11.19082
H	4.32189	-4.69651	10.52457

Compound 4 Excited State (S_1)

N	2.60440	0.68181	9.75647
N	3.14866	-0.06185	13.94640
N	3.33270	1.10574	14.51447
N	1.35158	3.52841	8.01205
N	2.95975	0.21308	12.66996
N	1.74843	4.81818	7.74624
N	2.48638	2.79293	7.68160
C	2.35178	0.45272	8.42105
C	2.11474	-0.92425	8.14357
C	2.24249	-1.59744	9.44326
C	1.81338	-1.34995	6.85466
H	1.63707	-2.40571	6.63410
C	2.27128	1.40807	7.39226

C	2.53500	-0.52407	10.36391
C	1.72829	-0.39820	5.82013
C	2.98930	4.87012	7.11043
C	1.94090	0.97048	6.11950
H	1.85334	1.71040	5.32129
C	3.44201	3.59598	7.05210
H	4.37929	3.19421	6.68125
C	3.02821	1.55858	12.41169
H	2.89067	1.92791	11.39410
C	2.69867	-0.83142	11.73424
C	2.13249	-2.89697	9.88448
H	1.90986	-3.71126	9.19038
C	3.39276	2.63560	16.42718
H	3.80119	2.72874	17.44413
H	2.30831	2.82587	16.50050
C	3.27445	2.15038	13.63341
C	3.63312	1.20435	15.95213
H	4.68585	0.90817	16.08496
H	3.00113	0.46990	16.46868
C	2.58530	-2.15600	12.17166
H	2.71105	-2.38179	13.22982
C	2.30658	-3.19148	11.26804
C	3.56599	6.19411	6.71669
H	4.30450	6.05754	5.91335
H	4.10752	6.62631	7.57795
C	4.04272	3.65245	15.48158
H	3.92048	4.67189	15.87411
H	5.12829	3.46368	15.42288
C	1.34289	7.19328	7.36421
H	1.76653	7.53273	8.32586
H	0.55565	7.91156	7.08982
C	0.71057	5.81435	7.54275
H	0.03277	5.78434	8.40874
H	0.11429	5.54384	6.64961
C	3.42149	3.57075	14.07891
H	2.41827	4.03166	14.08171
H	4.01444	4.12587	13.33692
C	2.44716	7.16160	6.30098
H	2.01694	6.83776	5.33684
H	2.86167	8.16902	6.14498
C	1.43135	-0.81364	4.40724
H	2.31487	-0.66625	3.76236
H	1.14237	-1.87214	4.34391
H	0.61884	-0.20519	3.97875
C	2.16713	-4.60776	11.74740
H	2.42028	-4.70876	12.81145
H	1.13164	-4.96312	11.60740
H	2.81580	-5.28527	11.16869

Compound ^{Li}5 Ground State (S_0)

I	2.19194	5.79346	7.68528
I	6.92115	7.22154	6.87443
Li	4.88384	6.22940	8.49280
Li	3.69477	3.91590	9.02966
Li	6.48235	8.26720	9.48680
O	2.78157	3.08466	10.55556
O	7.81351	7.59993	10.73573
N	5.34205	5.10123	10.17886
N	3.72608	7.52814	10.85423
N	2.44868	7.88229	11.00262
N	2.25540	8.66974	9.96182
N	6.51802	3.37545	8.07184
N	7.49237	3.43140	7.15685
N	6.80936	3.60804	6.04397
C	5.10974	5.60191	11.43837
C	4.32897	6.69477	11.83462
C	4.18561	6.99671	13.17598
H	3.57628	7.84835	13.44693
C	4.82561	6.25123	14.17711
C	5.64617	5.19717	13.80059
H	6.17011	4.62803	14.56005
C	5.79791	4.87336	12.45513
C	6.54907	3.87706	11.75724
C	6.22574	4.06434	10.38153
C	6.85542	3.23728	9.44269
C	7.77655	2.29424	9.85402
H	8.24829	1.67511	9.10269
C	8.09966	2.11613	11.20802
C	7.46691	2.90873	12.15464
H	7.70461	2.78189	13.20469
C	4.36874	8.03798	9.75414
C	3.35559	8.78645	9.17363
C	3.33270	9.60894	7.94032
H	3.92374	9.10497	7.17819
H	3.84668	10.54916	8.15000
C	0.91524	9.14368	9.63935
H	0.43414	9.45400	10.56349
H	0.37101	8.29180	9.23110
C	5.23118	3.50664	7.60898
C	5.46066	3.66527	6.24760
C	4.53390	3.94319	5.12062
H	4.16741	4.96549	5.23592
H	3.65292	3.31112	5.21380
C	5.21489	3.77322	3.76822
H	4.58916	4.20237	2.98699
H	5.32667	2.71127	3.53373
C	6.58307	4.44081	3.77357
H	7.05088	4.39222	2.79074
H	6.47630	5.49466	4.03284
C	7.50989	3.78025	4.77593
H	7.83174	2.79754	4.42876
H	8.39263	4.38243	4.97198
C	1.90945	9.86520	7.46025
H	1.91093	10.64639	6.70122
H	1.52078	8.95907	6.99452

C	1.00782	10.26327	8.62078
H	1.39145	11.16755	9.09833
H	0.00041	10.48917	8.27270
I	11.44075	12.55526	8.61954
I	6.72719	11.07797	9.40104
Li	8.75177	12.09560	7.79217
Li	9.91293	14.41873	7.32486
Li	7.18139	10.03062	6.78924
O	10.76588	15.40904	5.87119
O	5.87609	10.72176	5.52761
N	8.31553	13.24419	6.11467
N	9.94576	10.82026	5.44600
N	11.22844	10.47953	5.31287
N	11.41490	9.68461	6.34872
N	7.08629	14.95033	8.21361
N	6.08114	14.90124	9.09572
N	6.72433	14.69604	10.22769
C	8.57214	12.75383	4.85545
C	9.35491	11.66072	4.46431
C	9.52254	11.36982	3.12344
H	10.13301	10.51758	2.85710
C	8.90783	12.12814	2.11622
C	8.09123	13.18741	2.48585
H	7.58955	13.76972	1.72144
C	7.91532	13.50001	3.83103
C	7.16302	14.50058	4.52110
C	7.44824	14.29289	5.90267
C	6.79854	15.11390	6.83414
C	5.90339	16.07568	6.40880
H	5.41675	16.68979	7.15476
C	5.62302	16.27743	5.04879
C	6.26961	15.48616	4.11059
H	6.06268	15.62838	3.05600
C	9.29280	10.29511	6.53289
C	10.30519	9.55148	7.12087
C	10.32022	8.71784	8.34663
H	9.70701	9.20239	9.10379
H	9.82733	7.77065	8.11818
C	12.75562	9.22163	6.68511
H	13.25019	8.91911	5.76552
H	13.28676	10.07756	7.10206
C	8.35376	14.78828	8.71669
C	8.07720	14.61342	10.06672
C	8.95945	14.28977	11.21659
H	9.28504	13.25341	11.10208
H	9.86789	14.88540	11.15514
C	8.24540	14.47348	12.55004
H	8.83406	14.02015	13.34634
H	8.16071	15.53707	12.78829
C	6.85777	13.84873	12.50132
H	6.36218	13.91055	13.46971
H	6.94040	12.79260	12.24344
C	5.98145	14.53813	11.47311

H	5.67722	15.52856	11.81430
H	5.08747	13.96306	11.24844
C	11.73956	8.48124	8.84757
H	11.73813	7.69871	9.60527
H	12.10865	9.39224	9.31963
C	12.66227	8.09767	7.69878
H	12.29580	7.19023	7.21382
H	13.66742	7.88281	8.06009
C	9.12961	11.77680	0.67608
H	10.16618	11.94235	0.37744
H	8.90646	10.72796	0.47739
H	8.50123	12.37767	0.02207
C	4.64252	17.33392	4.63760
H	4.56075	17.39623	3.55451
H	3.64535	17.13115	5.03154
H	4.93311	18.31962	5.00360
C	4.62711	6.61151	15.61845
H	3.60172	6.42323	15.94144
H	5.28424	6.03281	16.26416
H	4.82817	7.66747	15.80212
C	9.11058	1.08281	11.60416
H	9.20372	1.01631	12.68610
H	8.84236	0.09229	11.23450
H	10.09880	1.31392	11.20303
C	5.05893	11.87276	5.69849
H	5.35892	12.66671	5.01651
H	4.00943	11.62192	5.52349
H	5.19220	12.21136	6.72094
C	5.88751	10.24041	4.19042
H	6.50400	9.34652	4.17947
H	4.87683	9.97755	3.86810
H	6.30779	10.98593	3.51406
C	10.14321	16.52431	5.24958
H	10.89389	17.20795	4.84435
H	9.47246	16.20457	4.45023
H	9.56319	17.03647	6.01198
C	11.54082	14.62766	4.96571
H	12.38103	15.21112	4.58141
H	11.91327	13.77353	5.52405
H	10.92749	14.27703	4.13426
C	3.44794	2.14519	11.38862
H	4.00560	2.65338	12.17681
H	2.73089	1.45256	11.83727
H	4.14304	1.59175	10.76417
C	1.89324	3.92873	11.28511
H	1.48632	4.64549	10.57858
H	1.08503	3.34008	11.72610
H	2.42886	4.46146	12.07273
C	7.81562	8.08753	12.07085
H	7.18497	8.97124	12.08768
H	7.41798	7.33869	12.75712
H	8.82695	8.36824	12.37544
C	8.64737	6.46187	10.55770

H	9.69460	6.73049	10.71888
H	8.36811	5.66491	11.24483
H	8.50768	6.11873	9.53763

Compound ^{Li}5^{truncated} Ground State (S_0)

I	2.27905	6.42976	7.58815
I	7.15066	7.64784	7.07711
Li	4.97474	6.48378	8.11400
Li	3.81092	4.49883	8.80338
Li	6.54307	7.70267	9.72078
O	3.05489	3.71451	10.36342
O	7.89799	7.51870	11.04156
N	5.49250	5.61407	9.95023
N	3.81536	7.90320	10.86850
N	2.54222	8.27962	10.88559
N	2.49597	9.18200	9.93245
N	6.60175	4.00087	7.69149
N	7.50177	3.95202	6.72410
N	6.79120	4.19472	5.64773
C	5.12925	5.88604	11.25802
C	4.28467	6.89789	11.76127
C	3.99508	6.96010	13.12157
H	3.33021	7.75377	13.46925
C	4.55868	6.04844	14.04291
C	5.45457	5.08574	13.56843
H	5.93438	4.39685	14.27008
C	5.74282	5.00402	12.20085
C	6.59518	4.14660	11.41489
C	6.36228	4.53974	10.05828
C	6.98424	3.78340	9.04449
C	7.88435	2.77100	9.37473
H	8.35431	2.21406	8.56185
C	8.16839	2.43720	10.71475
C	7.49385	3.12138	11.73241
H	7.67163	2.84450	12.77650
C	4.62713	8.52365	9.94307
C	3.70847	9.37477	9.32367
C	3.83755	10.27229	8.13377
H	3.94294	9.62526	7.24497
H	4.77516	10.84419	8.20184
C	1.22359	9.81363	9.57712
H	1.01108	10.59961	10.32105
H	0.44748	9.04055	9.65722
C	5.30327	4.26395	7.30285
C	5.46415	4.39365	5.92140
C	4.50595	4.77089	4.83718
H	4.27283	5.84421	4.95245
H	3.55059	4.25073	4.99587
C	5.08685	4.49935	3.44307
H	5.05176	3.41731	3.21992
H	4.46703	4.99770	2.68261
C	6.53381	4.99857	3.35062
H	6.94147	4.84534	2.33904
H	6.56411	6.08167	3.55871
C	7.44596	4.27986	4.34293
H	7.67489	3.25445	4.00820
H	8.39331	4.81594	4.49058
C	2.61730	11.18904	7.98058
H	2.66329	12.00549	8.72477
H	2.63262	11.66730	6.98951
C	1.31841	10.39498	8.16706
H	0.43688	11.03100	7.99031
H	1.28352	9.56709	7.43996
C	4.21854	6.14125	15.50928
H	3.15839	5.89721	15.69738
H	4.82688	5.44357	16.10350
H	4.39492	7.15646	15.90287
C	9.14950	1.33957	11.05221
H	8.67295	0.54564	11.65195
H	9.56479	0.86994	10.14750
H	9.99636	1.72697	11.64362
C	3.48858	2.51318	10.96830
H	3.80776	2.69592	12.00961
H	2.68581	1.75209	10.95362
H	4.35354	2.14481	10.39908
C	2.00544	4.38297	11.04772
H	1.80493	5.31599	10.50414
H	1.09151	3.76128	11.05871
H	2.31128	4.62220	12.08106
C	7.76632	7.68498	12.43974
H	6.73272	7.99039	12.64599
H	7.96672	6.73759	12.96831
H	8.46261	8.46139	12.80617
C	9.17548	7.05396	10.63109
H	9.97123	7.71710	11.01616
H	9.34818	6.02416	10.99321
H	9.19079	7.06611	9.53210

Compound ^{Li}5^{truncated} Excited State (S_1)

I	2.30741	4.28027	7.67331
I	6.71973	7.01028	6.71811
Li	4.72080	6.11796	8.42932
Li	3.99286	3.72471	9.58353
Li	6.24411	7.80603	9.23505
O	3.26776	2.98937	11.11718
O	7.50820	7.73612	10.61442
N	5.34332	5.13556	10.06727
N	3.62560	7.52244	10.77445
N	2.53543	8.26931	11.14185
N	2.40574	9.12646	10.09130
N	6.44691	3.31634	7.95248
N	7.43708	3.32125	7.07348
N	6.83297	3.48746	5.92461
C	5.09343	5.63738	11.35106

C	4.23520	6.70683	11.72880
C	4.02468	6.92751	13.09545
H	3.34925	7.73786	13.37137
C	4.67196	6.16781	14.10164
C	5.56898	5.17056	13.73464
H	6.08486	4.57891	14.49531
C	5.77382	4.90873	12.35906
C	6.53783	3.90472	11.65995
C	6.22673	4.09937	10.27253
C	6.77454	3.19153	9.34312
C	7.62607	2.17253	9.76657
H	8.03879	1.49695	9.01492
C	7.95946	2.00645	11.12656
C	7.39767	2.87935	12.06692
H	7.62702	2.74954	13.12858
C	4.16764	7.85305	9.50601
C	3.33706	8.86147	9.09020
C	3.31717	9.69389	7.84281
H	3.77399	9.12734	7.01815
H	3.94452	10.59416	7.98408
C	1.14241	9.80273	9.90425
H	0.86735	10.30371	10.84664
H	0.35259	9.05181	9.69596
C	5.19267	3.47486	7.40551
C	5.47436	3.59441	6.04735
C	4.62216	3.90494	4.85728
H	4.31014	4.95983	4.94811
H	3.70209	3.30501	4.89219
C	5.38168	3.69934	3.53978
H	4.81585	4.15850	2.71561
H	5.45823	2.62103	3.31197
C	6.78443	4.30973	3.62272
H	7.31453	4.22416	2.66155
H	6.71054	5.38198	3.86723
C	7.61979	3.61660	4.69546
H	7.92422	2.60527	4.38028
H	8.51885	4.19142	4.95358
C	1.88671	10.14664	7.51449
H	1.89482	10.85136	6.66764
H	1.28177	9.27595	7.20355
C	1.24303	10.79577	8.74598
H	1.84798	11.66610	9.05565
H	0.23408	11.17189	8.51458
C	4.38020	6.46969	15.55153
H	3.31485	6.30569	15.78857
H	4.97715	5.83644	16.22443
H	4.59865	7.52311	15.79612
C	8.91769	0.91823	11.54523
H	8.78464	0.65301	12.60531
H	8.78011	0.00330	10.94744
H	9.96868	1.23342	11.41562
C	3.84878	1.94104	11.87621
H	4.31147	2.34020	12.79315
H	3.08517	1.18829	12.14310
H	4.63077	1.47185	11.26372
C	2.33630	3.77836	11.85071
H	1.98871	4.58676	11.19524
H	1.47383	3.16292	12.16165
H	2.82144	4.22462	12.73296
C	7.26671	8.23981	11.91960
H	6.39594	8.90624	11.86418
H	7.03771	7.41956	12.61929
H	8.14520	8.80458	12.28301
C	8.54665	6.76986	10.54871
H	9.51115	7.21681	10.85252
H	8.31794	5.91140	11.20143
H	8.61191	6.42794	9.50670

Compound ^{MgBr}5 Ground State (S_0)

C	-3.07991	-3.37868	0.26122
C	-1.68272	-3.26080	0.36590
C	-1.04134	-2.02062	0.47828
C	-1.82180	-0.84194	0.49264
C	-3.24816	-0.96541	0.37599
C	-3.86082	-2.21413	0.26792
H	-1.06735	-4.15989	0.34720
H	-4.94823	-2.28693	0.17093
C	-2.58061	1.22314	0.45839
C	-2.74506	2.62655	0.38949
C	-4.03678	3.15655	0.30727
C	-5.18333	2.34168	0.26993
C	-5.02634	0.95011	0.28686
C	-3.74776	0.39392	0.36687
H	-4.14856	4.23971	0.26326
H	-5.90723	0.30396	0.23599
C	-3.72290	-4.74098	0.14702
H	-4.59403	-4.71921	-0.52651
H	-3.01530	-5.48920	-0.24228
H	-4.08297	-5.10701	1.12551
C	-6.55042	2.97672	0.18204
H	-6.73042	3.42153	-0.81297
H	-7.34600	2.23879	0.36606
H	-6.66988	3.78739	0.91882
C	1.18750	-0.83815	0.56379
C	2.46568	-1.38771	0.64899
C	-0.27957	3.16833	0.45398
C	0.34175	4.41457	0.39974
N	0.38965	-1.96989	0.54867
N	1.05751	-3.11814	0.61996
N	2.31612	-2.74487	0.67475
N	-1.61771	3.51182	0.37095
N	-1.84853	4.81823	0.26646
N	-0.64828	5.34949	0.28997
C	1.77659	4.82162	0.52360
H	2.07407	4.69260	1.57935

H	2.40630	4.11360	-0.03429
C	2.00340	6.26672	0.05610
H	2.00420	6.31293	-1.04790
H	2.99357	6.61345	0.38905
C	0.91731	7.20339	0.59805
H	1.10123	8.24053	0.27633
H	0.94121	7.19689	1.70134
C	-0.47456	6.79125	0.11850
H	-0.61330	7.02335	-0.95099
H	-1.27606	7.29488	0.67616
C	3.81998	-0.75515	0.71209
H	4.10129	-0.40941	-0.29962
H	3.74674	0.14503	1.34317
C	4.87691	-1.73381	1.24321
H	5.88312	-1.33069	1.05177
H	4.77392	-1.82494	2.33896
C	4.73877	-3.12027	0.60193
H	5.50806	-3.80835	0.98610
H	4.89250	-3.04618	-0.48878
C	3.36930	-3.73817	0.88417
H	3.29947	-4.07975	1.93028
H	3.15660	-4.59978	0.23644
N	-1.43533	0.47401	0.57030
Br	2.06224	1.84203	2.80523
Mg	0.44856	1.17753	0.98424

Compound ^{MgBr}5 Excited State (S$_1$)

C	-3.53182	-3.54608	0.22377
C	-2.11484	-3.47144	0.25255
C	-1.42359	-2.25304	0.26370
C	-2.20819	-1.08122	0.22044
C	-3.60923	-1.14742	0.22728
C	-4.29306	-2.38140	0.22809
H	-1.52195	-4.38447	0.26692
H	-5.38450	-2.42337	0.21674
C	-2.84656	1.03980	0.20890
C	-2.95334	2.46705	0.23137
C	-4.21959	3.04587	0.26390
C	-5.39246	2.26398	0.27347
C	-5.30068	0.84444	0.25721
C	-4.06466	0.23972	0.23108
H	-4.29616	4.13170	0.28860
H	-6.21576	0.24715	0.26964
C	-4.18299	-4.90386	0.17058
H	-5.27163	-4.84016	0.31557
H	-3.99896	-5.38828	-0.80432
H	-3.76958	-5.57496	0.94121
C	-6.73966	2.92495	0.27708
H	-7.16191	2.94788	-0.74448
H	-7.45561	2.37206	0.90501
H	-6.68555	3.96358	0.63391
C	0.85390	-1.16100	0.54118
C	2.09441	-1.73938	0.44011
C	-0.48703	2.91836	0.46446
C	0.18271	4.13468	0.31227
N	-0.01383	-2.23797	0.30449
N	0.61798	-3.43388	0.08291
N	1.92112	-3.09928	0.19492
N	-1.79737	3.29430	0.22384
N	-1.97167	4.58808	-0.06057
N	-0.75959	5.07594	0.00204
C	1.63091	4.49723	0.39931
H	1.91081	4.61199	1.46211
H	2.20959	3.65395	-0.00856
C	1.92170	5.79489	-0.36533
H	1.88381	5.59302	-1.45003
H	2.94230	6.14049	-0.14292
C	0.90474	6.88261	-0.00192
H	1.13280	7.83056	-0.51355
H	0.95009	7.08552	1.08213
C	-0.51414	6.46872	-0.38735
H	-0.66141	6.52777	-1.47776
H	-1.28215	7.09180	0.09206
C	3.47713	-1.17019	0.50985
H	3.44028	-0.11145	0.21550
H	3.85769	-1.21532	1.54833
C	4.42796	-1.95855	-0.40154
H	5.46431	-1.61065	-0.26388
H	4.16206	-1.76705	-1.45624
C	4.32456	-3.45804	-0.10189
H	4.58683	-3.63587	0.95631
H	5.03753	-4.03685	-0.71047
C	2.91533	-3.98329	-0.37878
H	2.76914	-4.99598	0.03118
H	2.74106	-4.04777	-1.47191
N	-1.76105	0.27035	0.17310
Br	1.90412	1.49708	-1.62519
Mg	0.26341	0.85052	0.14510

7. Supplementary References

1. T. R. Chan, R. Hilgraf, K. B. Sharpless and V. V. Fokin, *Org. Lett.*, 2004, **6**, 2853-2855.
2. I. Pryjomska-Ray, D. Zornik, M. Pätzel, K. B. Krause, L. Grubert, B. Braun-Cula, S. Hecht and C. Limberg, *Chem. Eur. J.*, 2018, **24**, 5341-5349.
3. I. Pryjomska-Ray, D. Zornik, M. Patzel, K. B. Krause, L. Grubert, B. Braun-Cula, S. Hecht and C. Limberg, *Chem. Eur. J.*, 2018, **24**, 5341-5349.
4. M. C. Tseng, H. T. Cheng, M. J. Shen and Y. H. Chu, *Org. Lett.*, 2011, **13**, 4434-4437.
5. K. Rurack and M. Spieles, *Anal. Chem*, 2011, **83**, 1232-1242.
6. S. Nad and H. Pal, *J. Phys. Chem. A*, 2001, **105**, 1097-1106.
7. L. Krause, R. Herbst-Irmer, G. M. Sheldrick and D. Stalke, *J. Appl. Crystallogr.*, 2015, **48**, 3-10.
8. G. Sheldrick, *Acta Crystallogr., Sect. A: Found. Crystallogr.*, 2015, **71**, 3-8.
9. C. B. Hubschle, G. M. Sheldrick and B. Dittrich, *J. Appl. Crystallogr.*, 2011, **44**, 1281-1284.
10. O. V. Dolomanov, L. J. Bourhis, R. J. Gildea, J. A. K. Howard and H. Puschmann, *J. Appl. Crystallogr.*, 2009, **42**, 339-341.
11. F. Neese, *Wiley Interdiscip. Rev. Comput. Mol. Sci.*, 2012, **2**, 73-78.
12. F. Neese, *Wiley Interdiscip. Rev. Comput. Mol. Sci.*, 2018, **8**, e1327.
13. S. H. Vosko, L. Wilk and M. Nusair, *Can. .J Phys.*, 1980, **58**, 1200-1211.
14. C. Lee, W. Yang and R. G. Parr, *Phys. Rev. B Condens. Matter*, 1988, **37**, 785-789.
15. A. D. Becke, *J. Chem. Phys.*, 1993, **98**, 5648-5652.
16. P. J. Stephens, F. J. Devlin, C. F. Chabalowski and M. J. Frisch, *J. Phys. Chem. A*, 1994, **98**, 11623-11627.
17. S. Grimme, J. Antony, S. Ehrlich and H. Krieg, *J. Chem. Phys.*, 2010, **132**, 154104.
18. S. Grimme, S. Ehrlich and L. Goerigk, *J. Comput. Chem.*, 2011, **32**, 1456-1465.
19. F. Neese, F. Wennmohs, A. Hansen and U. Becker, *Chem. Phys.*, 2009, **356**, 98-109.
20. R. Izsak and F. Neese, *J. Chem. Phys.*, 2011, **135**, 144105.
21. F. Weigend, *Phys. Chem. Chem. Phys.*, 2006, **8**, 1057-1065.
22. J. P. Perdew, *Phys. Rev. B*, 1986, **33**, 8822-8824.
23. A. D. Becke, *Phys. Rev. A*, 1988, **38**, 3098-3100.
24. T. Yanai, D. P. Tew and N. C. Handy, *Chem. Phys. Lett.*, 2004, **393**, 51-57.
25. Y. Zhao and D. G. Truhlar, *Theor. Chem. Acc.*, 2008, **120**, 215-241.
26. J. Tao, J. P. Perdew, V. N. Staroverov and G. E. Scuseria, *Phys. Rev. Lett.*, 2003, **91**, 146401.
27. V. N. Staroverov, G. E. Scuseria, J. Tao and J. P. Perdew, *J. Chem. Phys.*, 2003, **119**, 12129-12137.
28. J.-D. Chai and M. Head-Gordon, *Phys. Chem. Chem. Phys.*, 2008, **10**, 6615-6620.
29. A. Hellweg, C. Hättig, S. Höfener and W. Klopper, *Theor. Chem. Acc*, 2007, **117**, 587-597.
30. B. de Souza, F. Neese and R. Izsak, *J. Chem. Phys.*, 2018, **148**, 034104.
31. B. de Souza, G. Farias, F. Neese and R. Izsak, *J. Chem. Theory Comput.*, 2019, **15**, 1896-1904.
32. F. Weigend and R. Ahlrichs, *Phys. Chem. Chem. Phys.*, 2005, **7**, 3297-3305.
33. C. J. Cramer and D. G. Truhlar, *Acc. Chem. Res.*, 2008, **41**, 760-768.
34. M. D. Hanwell, D. E. Curtis, D. C. Lonie, T. Vandermeersch, E. Zurek and G. R. Hutchison, *J. Cheminformatics*, 2012, **4**, 17.
35. G. Knizia, *J. Chem. Theory Comput.*, 2013, **9**, 4834-4843.
